# RTS,S/AS01E immunization increases antibody responses to vaccine-unrelated *Plasmodium falciparum* antigens associated with protection against clinical malaria in African children: a case-control study

**DOI:** 10.1186/s12916-019-1378-6

**Published:** 2019-08-14

**Authors:** Carlota Dobaño, Itziar Ubillos, Chenjerai Jairoce, Ben Gyan, Marta Vidal, Alfons Jiménez, Rebeca Santano, David Dosoo, Augusto J. Nhabomba, Aintzane Ayestaran, Ruth Aguilar, Nana Aba Williams, Núria Díez-Padrisa, David Lanar, Virander Chauhan, Chetan Chitnis, Sheetij Dutta, Deepak Gaur, Evelina Angov, Kwaku Poku Asante, Seth Owusu-Agyei, Clarissa Valim, Benoit Gamain, Ross L. Coppel, David Cavanagh, James G. Beeson, Joseph J. Campo, Gemma Moncunill

**Affiliations:** 1 ISGlobal, Hospital Clínic - Universitat de Barcelona, Carrer Rosselló 153, E-08036 Barcelona, Catalonia Spain; 2 Centro de Investigação em Saúde de Manhiça (CISM), Rua 12, Cambeve, Vila de Manhiça, CP 1929 Maputo, Mozambique; 3 Noguchi Memorial Institute for Medical Research, University of Ghana, Accra, Ghana; 4 Kintampo Health Research Centre, Kintampo, Ghana; 5 Spanish Consortium for Research in Epidemiology and Public Health (CIBERESP), Madrid, Spain; 6 Malaria Vaccine Branch, Walter Reed Army Institute of Research, Silver Spring, MD USA; 7 Malaria Group, International Centre for Genetic Engineering and Biotechnology (ICGEB), New Delhi, India; 8 Malaria Parasite Biology and Vaccines Unit, Institut Pasteur, Paris, France; 9 Laboratory of Malaria and Vaccine Research, School of Biotechnology, Jawaharlal Nehru University, New Delhi, India; 10 Disease Control Department, London School of Hygiene and Tropical Medicine, London, UK; 11 Department of Osteopathic Medical Specialties, Michigan State University, 909 Fee Road, Room B 309 West Fee Hall, East Lansing, MI 48824 USA; 12 Department of Immunology and Infectious Diseases, Harvard T.H. Chen School of Public Health, 675 Huntington Ave., Boston, MA 02115 USA; 13 Université Sorbonne Paris Cité, Université Paris Diderot, Inserm, INTS, Unité Biologie Intégrée du Globule Rouge UMR_S1134, Laboratoire d’Excellence GR-Ex, Paris, France; 14 Infection and Immunity Program, Monash Biomedicine Discovery Institute and Department of Microbiology, Monash University, Melbourne, VIC Australia; 15 Institute of Immunology & Infection Research and Centre for Immunity, Infection & Evolution, Ashworth Laboratories, School of Biological Sciences, University of Edinburgh, King’s Buildings, Charlotte Auerbach Rd, Edinburgh, EH9 3FL UK; 16 Macfarlane Burnet Institute for Medical Research and Public Health, Melbourne, VIC Australia

**Keywords:** Malaria, *Plasmodium falciparum*, Vaccine, RTS,S, Antibody, Pre-erythrocytic antigens, Blood-stage antigens, Naturally acquired immunity, Protection, Maternal antibodies

## Abstract

**Background:**

Vaccination and naturally acquired immunity against microbial pathogens may have complex interactions that influence disease outcomes. To date, only vaccine-specific immune responses have routinely been investigated in malaria vaccine trials conducted in endemic areas. We hypothesized that RTS,S/A01E immunization affects acquisition of antibodies to *Plasmodium falciparum* antigens not included in the vaccine and that such responses have an impact on overall malaria protective immunity.

**Methods:**

We evaluated IgM and IgG responses to 38 *P. falciparum* proteins putatively involved in naturally acquired immunity to malaria in 195 young children participating in a case-control study nested within the African phase 3 clinical trial of RTS,S/AS01E (MAL055 NCT00866619) in two sites of different transmission intensity (Kintampo high and Manhiça moderate/low). We measured antibody levels by quantitative suspension array technology and applied regression models, multimarker analysis, and machine learning techniques to analyze factors affecting their levels and correlates of protection.

**Results:**

RTS,S/AS01E immunization decreased antibody responses to parasite antigens considered as markers of exposure (MSP1_42_, AMA1) and levels correlated with risk of clinical malaria over 1-year follow-up. In addition, we show for the first time that RTS,S vaccination increased IgG levels to a specific group of pre-erythrocytic and blood-stage antigens (MSP5, MSP1 block 2, RH4.2, EBA140, and SSP2/TRAP) which levels correlated with protection against clinical malaria (odds ratio [95% confidence interval] 0.53 [0.3–0.93], *p* = 0.03, for MSP1; 0.52 [0.26–0.98], *p* = 0.05, for SSP2) in multivariable logistic regression analyses.

**Conclusions:**

Increased antibody responses to specific *P. falciparum* antigens in subjects immunized with this partially efficacious vaccine upon natural infection may contribute to overall protective immunity against malaria. Inclusion of such antigens in multivalent constructs could result in more efficacious second-generation multistage vaccines.

**Electronic supplementary material:**

The online version of this article (10.1186/s12916-019-1378-6) contains supplementary material, which is available to authorized users.

## Background

Immunity against infectious diseases can be acquired by natural exposure to the microbe, or through intervention by vaccination, although in neither case is immunity necessarily sterile. Naturally acquired and experimentally induced immunity to infectious diseases are not necessarily mediated by the same host mechanisms, particularly in the case of pathogens that may have multiple and complex life cycle stages and where several potential immune effectors such as antibody vs cellular may act on distinct phases of infection. The way naturally acquired immunity (NAI) interacts with active immunization in clearing microbes in vaccinated subjects is multifaceted and not well understood. First, pre-existing immunity, as a result of prior exposure to infection and/or passively transferred maternal IgGs in the case of neonates and infants [1], may have an impact on response to vaccines and induction of protective efficacy. Second, immune responses induced by continuous exposure to pathogens before, during, and right after vaccination may also affect the type and magnitude of vaccine-induced immune responses and impact protective immunity against the disease and thus alter overall vaccine efficacy. Third, vaccination with partially or fully effective vaccines inducing moderate or strong immunity could decrease microbe exposure that is required for the induction and/or maintenance of NAI and this may result in a “rebound” in the incidence of disease if the vaccine is moderately efficacious and short-lived.

In the case of malaria caused by *Plasmodium falciparum*, in areas of heavy and continuous transmission, NAI is acquired with age and exposure and consequently the burden of disease is concentrated in children [2]. NAI is mediated mainly by IgG antibodies to antigens of the parasite asexual blood stage (BS) [3], but the specific epitope targets have not been unequivocally defined. The most advanced malaria vaccine globally, RTS,S/AS01E, has been tested in African subjects in phase 2 and 3 trials, showing consistent though moderate and waning efficacy against clinical malaria (range 55.8% in children to 31.3% in infants after 1 year of follow-up) [4, 5]. RTS,S elicits strong IgG antibodies to the circumsporozoite protein (CSP), the predominant protein of the *P. falciparum* pre-erythrocytic (PE) stage sporozoite, a response that has been implicated in vaccine-induced protection against malaria [6, 7], albeit inconsistently. Neither the effect that natural exposure and/or pre-existing immunity could have on RTS,S efficacy and longevity nor how vaccination affects NAI has been investigated in sufficient depth.

Based on clinical and immunogenicity data from previous phase 2b trials in Manhiça, Mozambique [8, 9], we proposed a model of development of protection to RTS,S that was dependent on the intricate interaction between vaccination and NAI, which might influence duration of vaccine efficacy [10]. We postulated that duration of vaccine efficacy depends on two distinct, but related, mechanisms: (1) initial partial PE protection via induction of vaccine-specific immune responses, which reduces the release of merozoites from the liver into the bloodstream, and (2) long-term protection resulting from enhancement of BS immunity facilitated through subclinical BS infection due to partial RTS,S protection (the “leaky vaccine” hypothesis). This represents a fourth, unexplored mechanism of vaccine interaction with NAI whereby reduced microbial burden resulting from partial vaccine efficacy may enhance NAI through a low-dose stimulus to the immune system [11]. Our previous analyses of Mozambican phase 2b trial samples measuring antibodies to a panel of *P. falciparum* antigens revealed that RTS,S vaccinees had similar or significantly lower IgG responses than comparator vaccines at 6 months after vaccination, particularly in younger children < 2 years of age [12]. Thus, there was no evidence of an enhancement of BS immunity through RTS,S vaccination. Rather, measured antibodies represented markers of exposure and reduction of antibody breadth and magnitude reflected vaccine efficacy [13].

In this study, using multiplex quantitative suspension array assays, we evaluated within the pediatric multicenter African RTS,S phase 3 clinical trial the impact of vaccination on NAI using an expanded panel of antigens that are putatively associated with malaria immunity and exposure. As NAI is dependent upon age and exposure, and may significantly affect vaccine efficacy, we included infant and children cohorts from two sites of different malaria transmission intensity (MTI). We investigated whether antibody responses 1 month post-immunization were modified by RTS,S and whether these responses contributed to malaria protective immunity.

## Methods

### Design

This study was carried out in two of the seven sites included in the multicenter immunology study MAL067, ancillary to the phase 3 randomized clinical trial MAL055 (NCT00866619): Kintampo in Ghana (moderate-high MTI) and Manhiça in Mozambique (low MTI) [6]. Briefly, our study included 109 infants aged 6–12 weeks and 86 children aged 5–17 months from the phase 3 trial who were randomly assigned to receive 3 doses of either the RTS,S/AS01E vaccine or a comparator vaccine (the meningococcal C conjugate in infants or rabies vaccines in children) during month (M) 0, 1, and 2. Subjects were followed up by passive case detection (PCD) starting at M0 and during the subsequent study months. For correlates of protection analyses in our study, we used a follow-up time of 12 months, when subjects were censored, and starting 14 days after sample collection at M3 (approximately 44 days after the third dose at M2). Subjects with ≥ 150 μL plasma/serum samples available at M0 (baseline) and M3 were selected. We included 129 RTS,S/AS01E- and 66 comparator-vaccinated children and infants from both sites. For the correlates of malaria protection/risk analysis, 78 children and infants were randomly selected from Kintampo, and 117 participants were selected from Manhiça according to a prior case-control study of cellular markers [14], and all were analyzed in a case-control design.

### Antibody assays

Quantitative suspension array technology (qSAT) applying the xMAP™ technology (Luminex Corp., Texas) was used to measure antibody responses to 38 *P. falciparum* antigens including three CSP constructs (Additional file 1: Table S1). Antigens were selected on the basis of profiling BS immunity, but also for the effect of vaccination on PE immune responses to sporozoite (SSP2/TRAP and CelTOS) and liver stages (LSA1). Although some of the BS antigens have been characterized as markers of exposure, such as AMA1 and MSP1 [15], antigen selection was primarily directed toward prominent targets of immunity, vaccine candidates, or prior association with protection in seroepidemiological studies or animal models. Additionally, several antigens were specifically included with said characteristics and limited polymorphism (e.g., RH2, RH4, RH5, and EBA140). VAR2CSA, a pregnancy-restricted variant of *P. falciparum* erythrocyte membrane proteins, was included as a representation of maternally derived antibodies.

qSAT assays contained bovine serum albumin (BSA) and glutathione S*-*transferase (GST)-coupled beads for background determination and as a control for signal from nonspecific binding of *P. falciparum* GST fusion proteins, respectively. *P. falciparum* proteins were covalently coupled directly to MagPlex beads and blocked with BSA. qSAT assays were previously standardized and optimized to control for sources of variability [16–18]. Briefly, antigen-coupled multiplex beads were mixed with 50 μL of test sample, negative or positive control [8, 19], at multiple dilutions (see Additional file 1). After incubation and washing, biotinylated secondary antibodies were added. Following streptavidin-R-phycoerythrin incubations, samples were acquired with a Luminex 100/200 analyzer and antibody levels measured as median fluorescence intensity (MFI). Data pre-processing is detailed in Additional file 1.

### Data analysis

Comparisons of crude Ig levels (log_10_ MFI) across antigens and isotypes were done through boxplots with geometric means, medians, and interquartile ranges (IQR), by *t* tests, and *p* values adjusted for multiple comparisons by the Holm approach [20]. Analyses included either all subjects or separately by visit and by vaccination, and in some cases stratifying by site, by age, and by age group within a site. To evaluate factors affecting M3 Ig levels to each antigen, we fitted first univariable and next multivariable linear regression models (coefficient, 95% confidence interval [CI], *p* values) including the following predictors: vaccination, sex, malaria transmission season at M3, having clinical malaria episodes between M0 and M3, and baseline variables like age cohort, antibody levels (using the same antigen/Ig as the outcome variable at M3), hemoglobin (Hb) concentrations, weight-for-age *Z*-score (WAZ), and height-for-age *Z*-score (HAZ). Models were also fitted separately at pre-vaccination (M0). Malaria transmission season was defined as high between April and October for Kintampo and November and April for Manhiça; the remaining months were defined as low transmission season. Linearity of the associations with continuous covariates was evaluated through penalized splines in generalized additive models (GAM); variables were modeled as linear. A combination of backward and forward stepwise algorithm was used in multivariable models.

Analysis of antibody correlates of protection against clinical malaria (fever > 37.5 °C with any parasitemia in the 12 months after M3.5) was based on a case-control design. Logistic regression models (odds ratio [OR], 95% CI, *p* values) were fitted first univariable and next multivariable to obtain the effect of different predictors in the odds of having malaria. Main predictors included levels (log_10_ MFI) of antibodies at M3 and change in antibody levels from M0 to M3. The change in antibody levels from M0 to M3 was calculated as the difference between log_10_ MFI levels at M3 and log_10_ MFI levels at M0 (log_10_ fold change [FC]) for each antibody-antigen pair. The impact of the other covariates (same as above) on the association between antibody responses and malaria risk/protection was also assessed. The linearity of the log_10_-transformed antibody levels was evaluated when the outcome was case-control. Multivariable models were obtained through the stepwise algorithm, R package MASS and function stepAIC. Both backwards and forward methods were combined to obtain the model with the minimum Akaike information criterion (AIC). All potential variables were proposed in the first step of the model, not only the significant ones.

Finally, we performed multimarker analysis by principal component analysis (PCA), correlation matrices, and machine learning partial least squares discriminant analysis (PLS-DA) using the R packages FactoMineR [21], Corrplot [22], and DiscriMiner [23], respectively. PLS-DA is a supervised method useful when there are many colinear variables. It is similar to PCA, but in addition it also takes into account a categorical response variable (clinical malaria or non-malaria in our case). Based on this, it creates new components with different loadings of the explanatory variables (antibody levels to the different antigens in our case) that better explain the response variable. For the PCA analysis, we included the log_10_-transformed levels of all antigen-isotype pairs at M3 to generate the principal components. We selected the first three principal components that best explained the variance of the data and tested these components on the variables of malaria, vaccination, age, and site. Correlations between IgG to all antigens were done by Spearman. For PLS-DA, the analysis was performed using the log_10_-transformed antibody levels of all antigen-isotype pairs at M3 for only RTS,S vaccinees. Then, we used the PLS-DA components to fit multivariable logistic regression models, including also age and site. Finally, we calculated the area under the curve (AUC) performance using the prediction of malaria outcome obtained with the PLS-DA and calculated overfitting parameters of the model (cumulative *R*^2^) to determine its quality. To check for areas of amino acid similarity in antigens with CSP that might cause cross-reactivity of RTS,S-induced antibodies, we aligned sequences using BLAST queries of each full-length antigen to the CSP. Additionally, we assessed the similarity of specific peptides by aligning 25 amino acid (a.a.) sequences overlapping by 7 a.a. against full-length CSP. We considered any hits with *E* value < 1 as significant, but also report the hits for the default threshold *E* value < 10 in Additional file 1.

## Results

### Baseline characteristics

We analyzed a total of 195 subjects (78 in Kintampo, Ghana, and 117 in Manhiça, Mozambique) with samples available at both pre-vaccination (M0) and post-vaccination (M3) [7]. RTS,S/AS01E (*n* = 129) and comparator (*n* = 66) vaccinees were similar with regard to baseline characteristics (age, sex, WAZ, HAZ, other vaccinations, previous malaria, season, distance to health center, Hb concentration), and most participants (93%) completed the 12-month post-vaccination follow-up [7]. The median time to drop-out of the study was 113 days (range 21–276), and most were early terminations due to loss to follow-up (7 subjects) or migration (4 subjects). A total of 89 malaria clinical events were recorded during the follow-up period: 60 in Kintampo (36 in RTS,S and 24 in comparators) and 29 in Manhiça (18 in RTS,S and 11 in comparators). Thirty-five clinical malaria events (39%) were registered in the children age cohort (48% in Kintampo, 21% in Manhiça), and the remaining in the infant age cohort. Parasitemia of subjects who had clinical malaria was comparable between RTS,S and comparator vaccinees. The Kaplan-Meier median follow-up time was 365 days (IQR = 128 to 365).

### *P. falciparum* antigens are grouped into decreasing, unaffected, and increasing antibody responses to RTS,S/AS01E vaccination

**Fig. 1:**
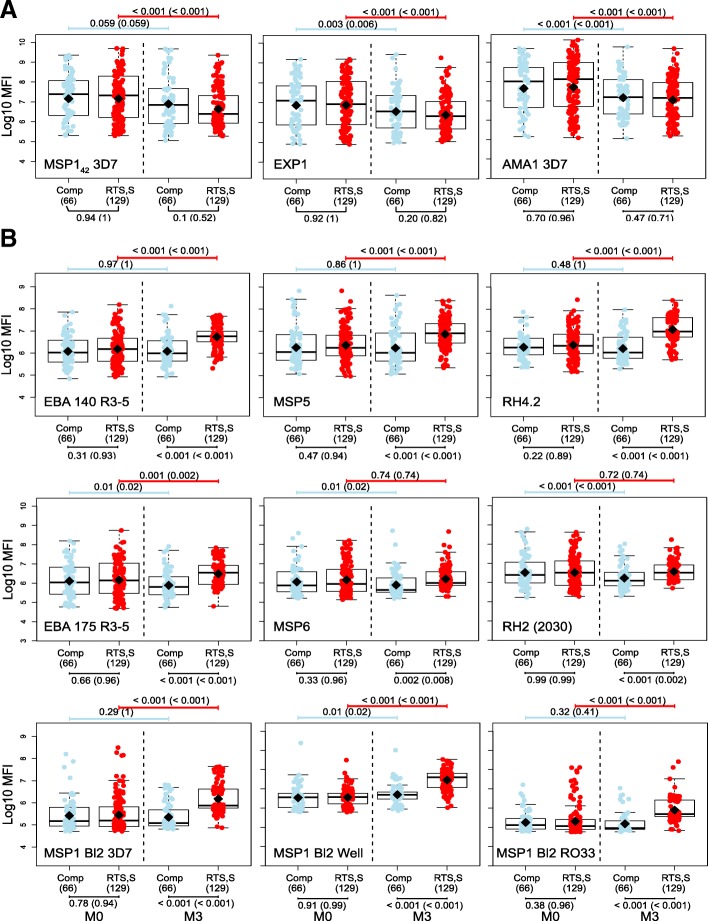
Effect of RTS,S/AS01E vaccination on IgG levels to non-RTS,S *P. falciparum* antigens at month 3. **a** Group i: Antibody levels at month 3 (M3) were lower in RTS,S than in comparator vaccinees. **b** Group iii: Antibody levels were higher at month 3 in RTS,S than in comparator vaccinees. Some representative examples are shown. Group ii antigens are illustrated in Additional file 1: Figure S1. Boxplots illustrate the medians and the 25th and 75th quartiles, whiskers display 1.5 times interquartile ranges, and diamonds show the geometric mean. Groups were compared through *t* tests and *p* values corrected for multiple comparisons using the Holm approach. Raw p values and adjusted p values in parenthesis are shown. log_10_ MFI, log_10_ of the median fluorescence intensity levels measured by quantitative suspension array technology

First, we aimed to assess the impact of RTS,S vaccination on the levels of IgG and IgM to vaccine-unrelated *P. falciparum* antigens 1 month post-vaccination (M3). We measured IgG and IgM levels (expressed as log_10_ MFI) (Additional file 1: Table S1) at M0 and M3 and analyzed the effect of vaccination in univariable and multivariable models adjusting for age, site, baseline levels at M0, and previous malaria episodes from M0 to M3. We defined three different groups of antibodies based on the three different patterns of antibody responses that emerged upon RTS,S immunization, primarily based on IgG levels (Additional file 1: Table S2 multivariable models and Table S3 univariable models): decrease in antibody levels (“group i” antigens, Fig. 1a); no change in antibody levels (“group ii” antigens, Additional file 1: Figure S1a); increase in antibody levels (“group iii” antigens, Fig. 1b). IgG levels to MSP1_42_, EXP1, and AMA1 were significantly (or borderline statistically significant) lower in RTS,S vs comparators vaccinees at M3 (Additional file 1: Table S2). In contrast, IgG levels to EBA140, EBA175 R3–5, MSP1 Block (Bl) 2 (3D7, Well, RO33, and MAD20 strains), MSP5, MSP6, RH2 2030, RH4.2, RH5, and SSP2 (TRAP) were significantly higher in RTS,S vs comparators vaccinees at M3 (Additional file 1: Table S2) and/or vs baseline levels in RTS,S vaccinees only (Fig. 1b). More specifically, RTS,S vaccination increased from 1.52 to 6.55 times the M3 IgG levels against group iii antigens compared to comparators. IgM levels remained largely unaffected by RTS,S vaccination (Additional file 1: Figure S1b) except for MSP1_42_ 3D7 that was significantly higher in comparators, as was seen for IgG to this antigen, and SSP2 that was significantly higher in RTS,S vaccinees (Additional file 1: Table S3a). To sum up, RTS,S vaccination differently affected the IgG levels 1 month post-vaccination, depending on the antigens.

### Effect of other variables on antibody responses to *P. falciparum* antigens

Next, we wanted to understand the effect of the study covariates on the M3 antibody levels measured, which could help explain the three patterns of responses that we observed upon vaccination. Comparing antibody levels over time, IgGs did not change markedly from M0 to M3. However, for some group i and ii antigens (AMA1, EXP1, MSP2, MSP3 3D7, EBA175 R2 F2, and the pregnancy-related VAR2CSA DBL1–2 and DBL3–4), IgG levels decreased with age from M0 to M3 (Fig. 1a) probably reflecting decay of maternal antibodies. Children had higher IgG levels than infants for most of the antigens at M3 in adjusted models (Additional file 1: Table S2), particularly for group ii (not affected by RTS,S) and group iii (increased by RTS,S) antigens. At M0, IgG levels to group i antigens were higher in infants than in children in adjusted models, and there was a mixed pattern for group ii and iii antigens (Additional file 1: Table S4b). IgM levels to most antigens were significantly higher in children than in infants at M0 and were similar between age groups or higher in children at M3 (Additional file 1: Tables S2 and S3).

Regarding the effect of geographical location (site), M0 IgG levels for all antigens and IgM levels for most antigens were significantly higher in Kintampo than in Manhiça in adjusted models (Additional file 1: Table S4b), reflecting the higher MTI in Kintampo. However, many antibodies were not significantly different at M3 by site in adjusted models, and an inverse pattern was seen for IgG to many group iii antigens, MSP3 and LSA1, whereby levels were higher in Manhiça than in Kintampo (Additional file 1: Table S2).

Higher baseline Hb concentrations and WAZ (and HAZ to a lesser extent) were significantly associated with lower M0 IgM levels to most antigens in adjusted models, and some similar significant associations were found for IgG (Additional file 1: Table S4b). Sex had no significant associations with baseline IgG or IgM levels. The strongest and most consistent associations were for previous episodes of clinical malaria and higher M0 antibody levels (to the homologous antigen), both predicting higher M3 antibody levels (Additional file 1: Table S4). The magnitude of the associations, particularly for prior malaria episodes, was higher with greater significance for IgG than for IgM. Group i and ii antigens (MSP1_42_, EXP1, MSP2) were more affected by previous malaria.

**Table 1:** Effect of IgG levels to *Plasmodium falciparum* antigens on malaria protection

Antibody	Month 3 IgG levels		Age (5–17 months)		Site (Manhiça)		Baseline IgG levels		WAZ		Vaccine (RTS,S)	
Antigen	OR	*p*	OR	*p*	OR	*p*	OR	*p*	OR	*p*	OR	*p*
Group i antigens												
MSP1_42_ 3D7	1.11 (0.7; 1.74)	0.65			*0.18 (0.08; 0.39)*	*< 0.001*	1.47 (0.96; 2.28)	0.08				
MSP1_42_ FVO	0.87 (0.57; 1.29)	0.48	0.5 (0.23; 1.04)	0.07	*0.13 (0.05; 0.29)*	*< 0.001*	*1.47 (1.02;2.15)*	*0.04*	0.78 (0.56; 1.08)	0.13	0.59 (0.28; 1.2)	0.15
EXP1	0.8 (0.47; 1.33)	0.39			*0.19 (0.08; 0.43)*	*< 0.001*	*1.94 (1.24; 3.12)*	*0.005*	0.75 (0.54; 1.05)	0.1	0.53 (0.25; 1.1)	0.09
AMA1 FVO	*1.6 (1.15; 2.27)*	*0.006*			*0.13 (0.06; 0.25)*	*< 0.001*						
AMA1 3D7	0.98 (0.51; 1.83)	0.94			*0.14 (0.07; 0.28)*	*< 0.001*	1.54 (0.93; 2.67)	0.1	0.78 (0.56; 1.08)	0.14	0.56 (0.27; 1.15)	0.12
Group ii antigens												
pTRAMP	1.01 (0.51; 2.03)	0.98	*0.42 (0.2; 0.85)*	*0.02*	*0.12 (0.06; 0.25)*	*< 0.001*	*2.18 (1.16; 4.21)*	*0.02*			*0.54 (0.26; 1.11)*	*0.1*
EBA175 R2(F2)	0.96 (0.56; 1.64)	0.89	*0.49 (0.24; 0.98)*	*0.047*	*0.09 (0.04; 0.18)*	*< 0.001*						
PfRH1	1.58 (0.79; 3.19)	0.2	*0.4 (0.18; 0.83)*	*0.02*	*0.11 (0.05; 0.23)*	*< 0.001*			0.79 (0.56; 1.09)	0.15		
MSP3 3D7	1.32 (0.77; 2.3)	0.32	0.51 (0.25; 1.02)	0.06	*0.1 (0.05; 0.2)*	*< 0.001*						
Var2csa DBL3–4	1.03 (0.5;2.1)	0.94	0.5 (0.21; 1.13)	0.1	*0.09 (0.04; 0.2)*	*< 0.001*						
p41	1.41 (0.64; 3.16)	0.4	*0.46 (0.22; 0.94)*	*0.04*	*0.1 (0.05; 0.19)*	*< 0.001*						
MSP2 FL Dd2	0.89 (0.47; 1.61)	0.71			*0.25 (0.11; 0.57)*	*0.001*	*2.15 (1.31; 3.67)*	*0.003*	0.76 (0.54; 1.06)	0.11	0.52 (0.25; 1.07)	0.08
MSP2 FL CH150	1.02 (0.57; 1.83)	0.96			*0.25 (0.11; 0.58)*	*0.001*	*2.05 (1.31; 3.29)*	*0.002*	0.76 (0.54; 1.06)	0.11	0.44 (0.2; 0.92)	0.03
MSP1 Bl2 hybrid	1.11 (0.68; 1.83)	0.67	*0.5 (0.24; 0.98)*	*0.049*	*0.09 (0.04; 0.19)*	*< 0.001*						
MSP3 3C	1.22 (0.74; 2.01)	0.43	*0.41 (0.19; 0.84)*	*0.02*	*0.1 (0.05; 0.22)*	*< 0.001*	1.67 (0.94; 2.99)	0.08			0.55 (0.27; 1.14)	0.11
PfRH2 b240	1.03 (0.49; 2.18)	0.94	*0.49 (0.24; 0.98)*	*0.049*	*0.09 (0.04; 0.19)*	*< 0.001*						
LSA1	0.88 (0.51; 1.49)	0.64	0.5 (0.24; 0.99)	0.051	*0.09 (0.04; 0.18)*	*< 0.001*						
MSP1 Bl2 PA17	0.96 (0.53; 1.7)	0.89	*0.5 (0.24; 0.98)*	*0.048*	*0.09 (0.04; 0.18)*	*< 0.001*						
Var2csa DBL1–2	0.9 (0.37; 2.27)	0.82			*0.14 (0.07; 0.29)*	*< 0.001*	*2.06 (1.01; 4.26)*	*0.047*			0.57 (0.28; 1.16)	0.12
CyRPA	1.81 (0.76; 4.43)	0.18	*0.4 (0.18; 0.82)*	*0.02*	*0.1 (0.04; 0.19)*	*< 0.001*			0.79 (0.57; 1.09)	0.15	0.57 (0.28; 1.17)	0.13
CelTOS	1.55 (0.77; 3.13)	0.22	*0.41 (0.19; 0.85)*	*0.02*	*0.1 (0.05; 0.2)*	*< 0.001*			0.79 (0.57; 1.09)	0.16	0.58 (0.28; 1.18)	0.13
RH4.9	0.62 (0.3; 1.26)	0.19	0.53 (0.26; 1.05)	0.07	*0.08 (0.04; 0.16)*	*< 0.001*						
Group iii antigens												
DBL-α	1.35 (0.62; 2.91)	0.44	*0.45 (0.21;0.92)*	*0.03*	*0.12 (0.05; 0.24)*	*< 0.001*	1.9 (0.88; 4.22)	0.11				
RH5	1.93 (0.98; 4.01)	0.07	*0.38 (0.17; 0.79)*	*0.01*	*0.11 (0.05; 0.21)*	*< 0.001*			0.78 (0.56; 1.08)	0.15	0.51 (0.24; 1.07)	0.08
SSP2 (TRAP)	*0.52 (0.26; 0.98)*	*0.05*			*0.11 (0.05; 0.23)*	*< 0.001*	*2.29 (1.14; 4.8)*	*0.02*				
MSP1 Bl2 Mad20	0.61 (0.13; 2.7)	0.52	0.55 (0.26; 1.13)	0.11	*0.14 (0.07; 0.3)*	*< 0.001*	*5.91 (1.5; 29.52)*	*0.02*	0.79 (0.57; 1.09)	0.15		
MSP6	1.19 (0.56; 2.61)	0.65	*0.47 (0.21; 0.98)*	*0.05*	*0.14 (0.06; 0.3)*	*< 0.001*	1.63 (0.89; 3.01)	0.12	0.78 (0.56; 1.07)	0.12	0.51 (0.23; 1.09)	0.08
RH2 (2030)	1.37 (0.72; 2.65)	0.35	0.51 (0.25; 1.01)	0.059	*0.1 (0.04; 0.19)*	*< 0.001*					0.55 (0.26; 1.16)	0.12
EBA175 R3–5	1.17 (0.71; 1.96)	0.54	*0.45 (0.21; 0.91)*	*0.03*	*0.1 (0.05; 0.2)*	*< 0.001*			0.79 (0.57; 1.09)	0.16	0.55 (0.25; 1.18)	0.13
MSP5	0.78 (0.48; 1.25)	0.3	0.49 (0.23; 1)	0.056	*0.12 (0.06; 0.24)*	*< 0.001*	*2.06 (1.26; 3.45)*	*0.005*				
EBA140 R3–5	1.21 (0.69; 2.14)	0.5	*0.47 (0.22; 0.94)*	*0.04*	*0.09 (0.04; 0.18)*	*< 0.001*					0.54 (0.24; 1.19)	0.13
MSP1 Bl2 RO33	*0.53 (0.3; 0.93)*	*0.03*	0.53 (0.25; 1.07)	0.08	*0.1 (0.04; 0.21)*	*< 0.001*	1.64 (0.93; 3.03)	0.1				
MSP1 Bl2 Well	*0.55 (0.3; 0.99)*	*0.05*	0.51 (0.23; 1.07)	0.08	*0.1 (0.05; 0.2)*	*< 0.001*	2.06 (0.95; 4.72)	0.08				
MSP1 Bl2 3D7	0.63 (0.39; 1)	0.054	*0.47 (0.22; 0.96)*	*0.042*	*0.08 (0.04; 0.17)*	*< 0.001*			0.79 (0.56; 1.09)	0.16		
RH4.2	0.85 (0.52; 1.38)	0.51	0.53 (0.25; 1.06)	0.08	*0.09 (0.04; 0.18)*	*< 0.001*						

Each line shows the multivariable logistic regression model’s results with IgG levels to each antigen (expressed as log_10_ of the median fluorescence intensity measured by quantitative suspension array technology) at month 3 as predictors and clinical malaria as outcome, adjusted by covariates. Columns show the odds ratio (OR) with 95% confidence intervals and *p* values (significant in italics) of the month 3 IgG levels and the covariables retained in the models for some antigens (in parenthesis the category that is compared to the reference). Variables that were not significant or did not improve the multivariable model are not shown. *WAZ* weight-for-age *Z*-score, *FL* full length, Bl2 block 2

Overall, these results suggest that antigens from groups i and ii are more immunogenic and reflect better malaria exposure than antigens from group iii.

### Correlations of RTS,S and non-vaccine-related antibody responses

**Fig. 2:**
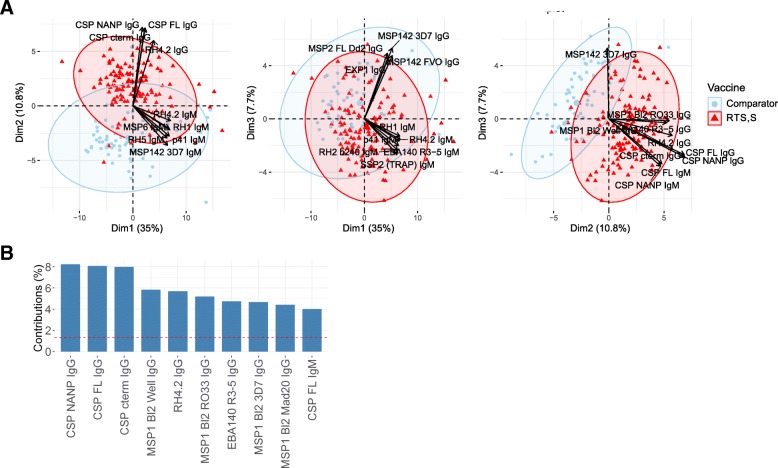
Relationship among antibody responses to RTS,S and non-RTS,S antigens after RTS,S/AS01E vaccination. **a** Principal component analysis (PCA) plots of individuals clustered by vaccine type. The first three principal components (Dim 1, Dim 2, and Dim 3) that explained the highest percentage of the variance of the IgG and IgM data at month 3 (percentage in parenthesis) were chosen for representation. **b** Contribution of the top 10 variables to the PCA Dim 2. The red dashed line indicates the expected average contribution. Any variable with a contribution above this cutoff can be considered as important in contributing to the component

We performed PCA of all M3 antibody responses, including IgG and IgM levels to RTS,S antigens (NANP, C-terminus, and CSP full length [7]), to reduce the dimensionality of the data and get insights into the relationship between vaccine and non-vaccine antibody responses. Study participants clustered by vaccine group (Fig. 2a) and principal component 2 (PC2 or dimension 2) separated both clusters. The 10 antibody responses contributing more to PC2 were, as expected, IgG against the three CSP constructs, IgM to CSP full length, but also group iii antigens: RH4.2 (that was highly correlated with CSP), four MSP1 Bl2 constructs, and EBA140 (Fig. 2a, b).

**Fig. 3:**
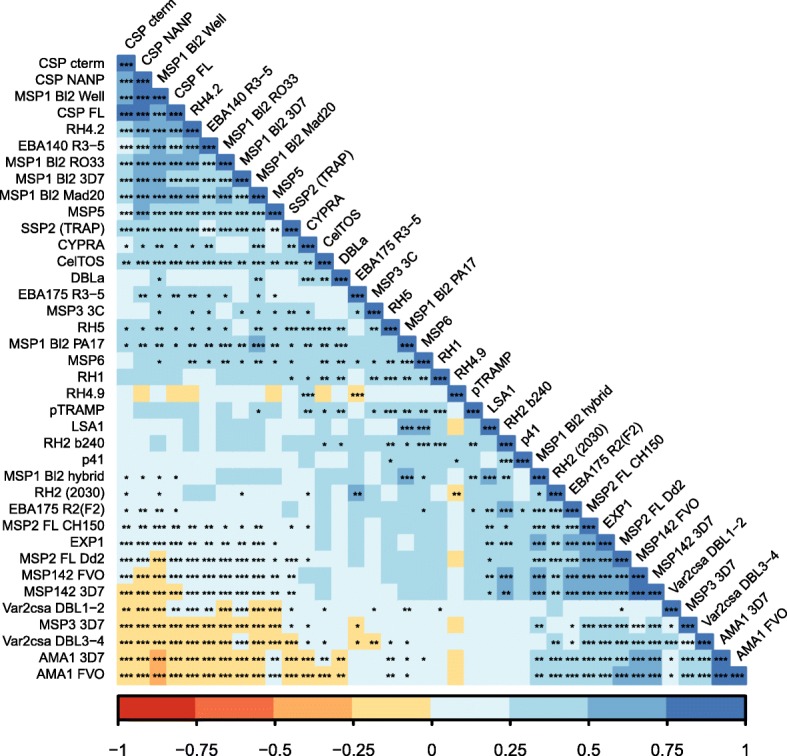
Correlation among IgG responses in RTS,S vaccinees. Colors indicate Spearman coefficients (rho). *p* values: *<  0.05, **<  0.01, ***<  0.001

Correlations among IgG responses to non-RTS,S antigens of groups i, ii, and iii, in addition to RTS,S vaccine antigens, in RTS,S vaccinees, are depicted in Fig. 3. Three main patterns were observed: (1) most group iii antigens and CSPs significantly correlated among themselves, although to varying degrees (rho between 0.25 and 1); (2) MSP1_42_, AMA1, MSP3 3D7, and VAR2CSA (DBL1–2 and DBL3–4) correlated negatively (rho between − 0.25 and 0) with CSPs, and together with MSP2 full-length Dd2 correlated negatively with MSP1 Bl2 Well (rho between − 0.5 and 0), and VAR2CSA (DBL1–2 and DBL3–4), MSP3 3D7, and AMA1 correlated negatively with most group iii antigens and some group ii antigens (rho between − 0.25 and 0); and (3) some group ii and all group i and iii antigens correlated positively among themselves (mainly with rho > 0.25) (Fig. 3). In addition, group ii antigen RH4.9 correlated negatively with CSP and some antigens of all three groups.

To assess whether antibody responses correlating with CSP could be an artifact of cross-reactive CSP antibodies, we aligned the *P. falciparum* 3D7 strain ortholog of each protein in this study with CSP to check for regions of local a.a. similarity. Among all proteins, including the GST tag, only SSP2 showed significant primary structure with *E* value < 1 for full-length queries (a.a. 249–286, % identity = 37.8, *E* value = 1.44E−05), aligning with the thrombospondin type 1 domain-containing CSP a.a. 330–374. At the peptide level (7 a.a. overlapping 25mers), two sequential SSP2 fragments had significant similarity corresponding to a.a. 235–259 (% identity = 90.9, *E* value = 0.002) and a.a. 253–277 (% identity = 66.7, *E* value = 0.17), the region of SSP2 containing a C-terminal thrombospondin type 1 domain. Additional hits using the default *E* value threshold of 10 are described in Additional file 1.

### Antibodies to group i and ii antigens had no effect or were associated with increased risk of clinical malaria

**Fig. 4:**
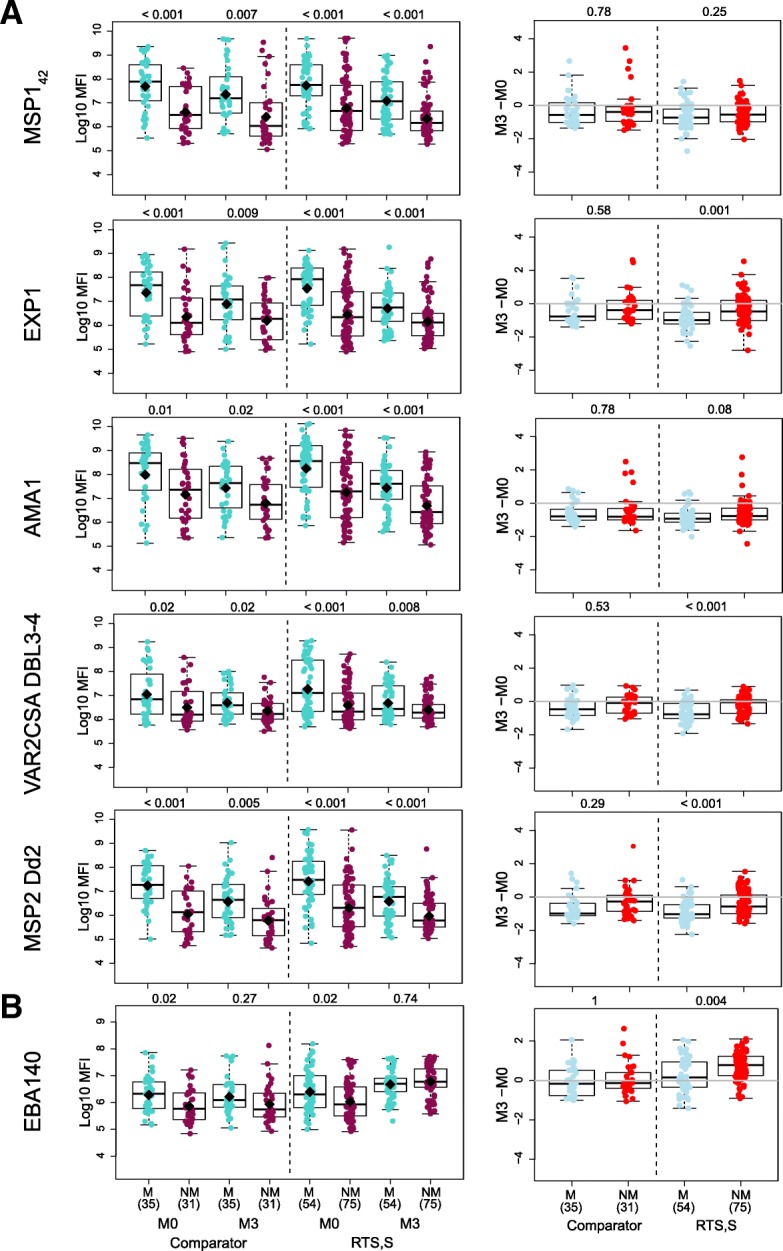
Association between IgG responses to non-RTS,S antigens and malaria protection. **a** Antigen groups i and ii. **b** Antigen group iii. Levels of IgG and change from pre- (M0) to post-vaccination (M3). Some representative examples of the distinct patterns observed in each group are shown. Boxplots illustrate the medians and the 25th and 75th quartiles, whiskers display 1.5 times interquartile ranges, and diamonds show the geometric mean. Groups were compared through *t* tests and *p* values corrected for multiple comparisons using the Holm approach are shown. M malaria, NM no malaria, log_10_ MFI log_10_ of the median fluorescence intensity (MFI) levels measured by quantitative suspension array technology, M3-M0 change between M0 and M3 antibody levels expressed as log_10_ MFI

We next wanted to decipher the role of the antibody responses measured in protection against clinical malaria. Having higher baseline IgM to most *P. falciparum* antigens, and higher baseline IgG to half of the antigens, was associated with occurrence of clinical malaria during the 12-month follow-up period after M3.5, as illustrated in Fig. 4 for IgG. In the univariable analysis, M3 antibodies to group i antigens and some group ii antigens that are considered markers of exposure (including maternal exposure) were higher in malaria cases than in controls (Fig. 4); stratified by age, this was more significant in children (Additional file 1: Figure S3). IgG levels to the above antigens that were significantly higher in infants than in children with malaria at baseline and in comparators were presumably derived from the mother (Additional file 1: Figure S3). In the multivariable analysis, these risk associations disappeared except for IgG to group i antigen AMA1 FVO (Table 1) and IgM to RH2 (2030) (OR = 3.66 [1.44; 10.03], *p* = 0.008).

**Fig. 5:**
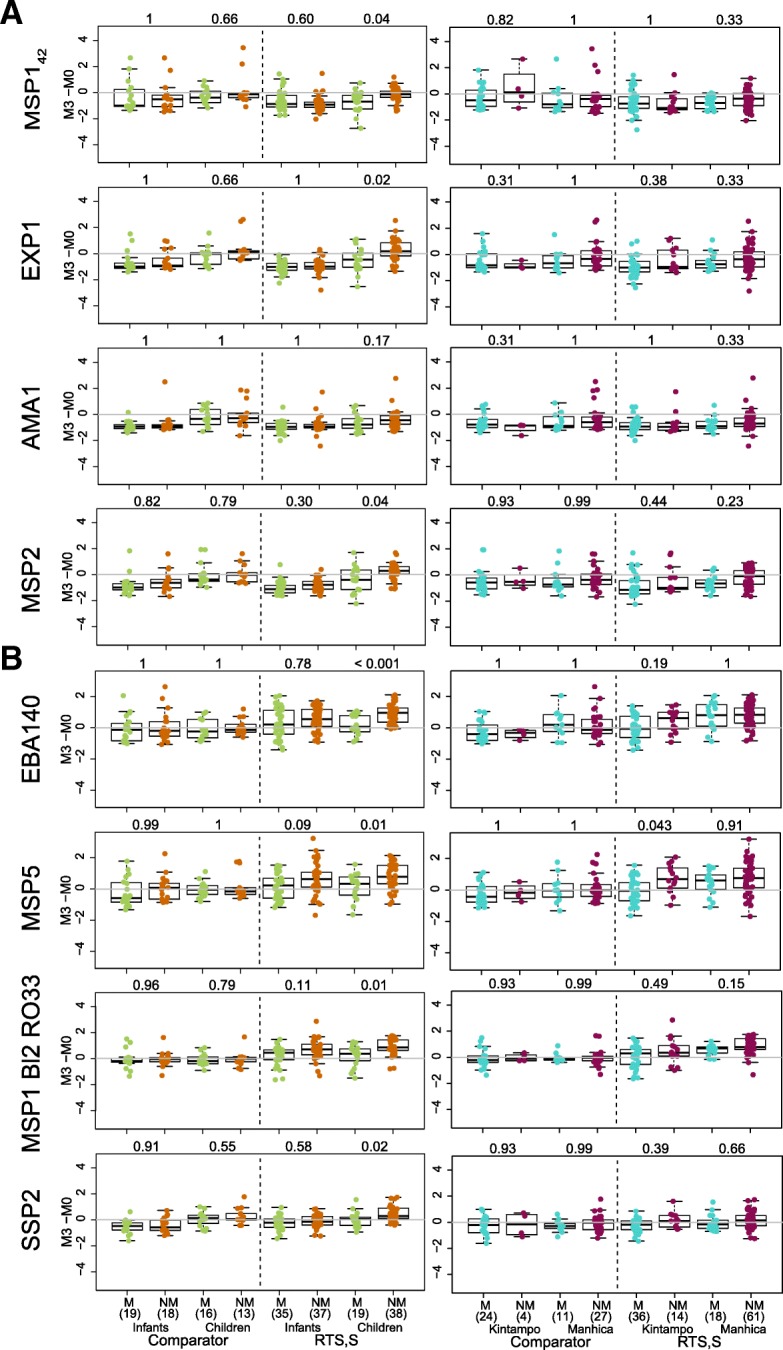
Association between IgG levels to non-RTS,S antigens and malaria protection stratified by age and site. **a** Antigen groups i and ii. **b** Antigen group iii. Change from pre- (M0) to post-vaccination (M3) antibody levels. Some representative examples are shown. Boxplots illustrate the medians and the 25th and 75th quartiles, whiskers display 1.5 times interquartile ranges, and diamonds show the geometric mean. Groups were compared through *t* tests and *p* values corrected for multiple comparisons using the Holm approach are shown. M malaria, NM no malaria, M3-M0 change between M0 and M3 antibody levels expressed as log_10_ of the median fluorescence intensity (MFI) measured by quantitative suspension array technology

Analysis of the change in IgG levels between M0 and M3 (log_10_ FC) showed that there were either no significant effects of log_10_ FC on malaria risk or, if there were, IgG levels declined from M0 to M3, and the decrease was lower in non-malaria controls than in malaria cases (Fig. 4). The drop in antibodies from M0 to M3, more pronounced in infants, and the lower decrease in protected subjects, could be due to more production and/or less antibody decay from M0 to M3. In fact, age-stratified analyses (Fig. 5 and Additional file 1: Figure S3) showed that malaria-protected children had an increase of IgG against EXP1 and MSP2 from M0 to M3, whereas children with malaria cases had a decrease and, therefore, significantly higher log_10_ FC in protected children. In multivariable analysis, IgG log_10_ FC was associated with malaria protection for EXP1 (OR = 0.6 [0.38; 0.91], *p* = 0.02), MSP2 Dd2 (OR = 0.54 [0.32; 0.87], *p* = 0.02), and MSP2 CH150 (OR = 0.58 [0.37; 0.88], *p* = 0.01, also for IgM).

### Antibodies to group iii antigens were associated with reduced risk of clinical malaria

In contrast, higher M3 IgG levels to three group iii antigens, SSP2 and MSP1 Bl2 (strains RO33 and Well, borderline for 3D7), were significantly associated with reduced risk of clinical malaria (Table 1). In these multivariable analyses, RTS,S vaccination, children age cohort, Manhiça site, low baseline antibody levels, and high WAZ were associated (statistically significantly or not) with lower risk of malaria (Table 1). Sex, prior malaria episodes, season, HAZ, or Hb concentrations did not significantly contribute to the models for any antigen. In addition, non-malaria controls had significantly higher log_10_ FC than malaria cases (Fig. 4), unlike the pattern seen for some group i and ii antigens. Stratifying by age group and site, the protective association was seen only for children and mainly in samples from Kintampo (Fig. 5b). In the multivariable analysis, an association with protection between higher IgG log_10_ FC and malaria was significant for MSP5 (OR = 0.62 [0.41; 0.91], *p* = 0.02) in addition to SSP2 and MSP1 Bl2 RO33 and Well strains, for which M3 levels were also significantly associated with protection (Table 1), and additionally for MSP1 Bl2 Mad20 (OR = 0.3 [0.09; 0.88], *p* = 0.03).

### Multimarker analyses of antibody levels and malaria protection

**Fig. 6:**
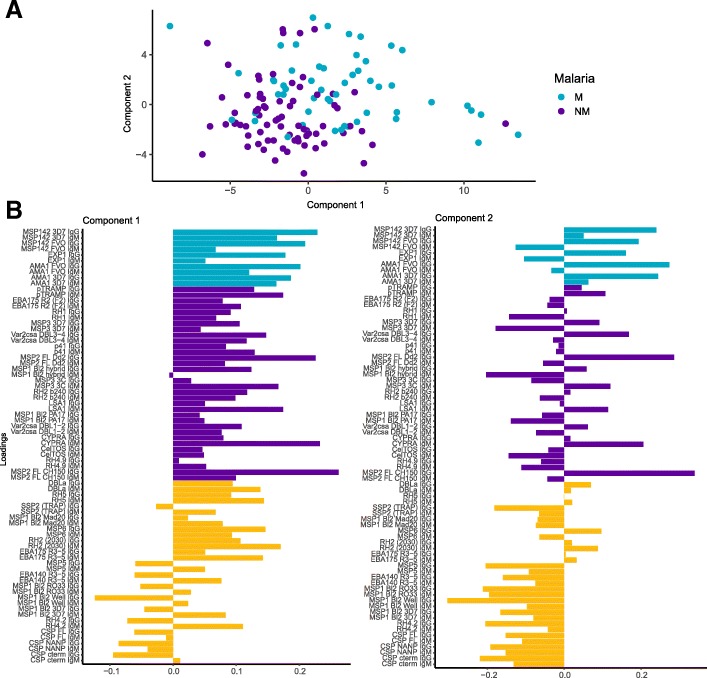
Multimarker analysis of antibody responses to RTS,S and non-RTS,S antigens after vaccination and malaria protection. **a** Partial least squares discriminant analysis (PLS-DA) scatter-plot with the two PLS-DA components that best enable discrimination between malaria cases (blue) and non-malaria controls (pink). **b** Loadings of antibody response for each PLS-DA component colored by antigen group: group i (light blue), group ii (dark purple), group iii plus vaccine antigens (yellow)

To perform multimarker analysis accounting for colinearity in antibody responses, we performed PLS-DA of antibodies at M3 among RTS,S vaccinees, with malaria as a response variable (Fig. 6a, b), and identified two components associated with the outcome. Interestingly, the loadings of component 1 (Fig. 6b) showed a pattern in which roughly all group i and ii antigens and almost half of group iii antigens were associated with increased malaria risk. IgG levels to the other half of group iii antigens (SSP2, MSP5, EBA140, MSP1 Bl2 [RO33, Well, 3D7], RH4.2) and CSP (C-terminus, NANP, and full length) were associated with protection, whereas almost all IgM levels to the same antigens were associated with increased risk. In component 2, there was a mixed pattern for groups i and ii antigens, while IgG and IgM to exactly the same group iii antigens as component 1 were associated with protection. In a multivariable logistic model including these two PLS-DA components and adjusted by age and site, the PLS-DA components were independently associated with malaria (component 1: OR = 1.26 [1.09; 1.48], *p* = 0.002 and component 2: OR = 1.31 [1.06; 1.66], *p* = 0.016), while age was not (OR = 1.22 [0.38; 3.93], *p* = 0.73) and site was borderline (OR = 0.33 [0.11; 1.01], *p* = 0.05). The predictive ability of the model was moderate (33 out of 54 malaria cases and 64 out of 75 controls correctly predicted) with an AUC of 0.732 and a cumulative *R*^2^ of 0.397.

**Fig. 7:**
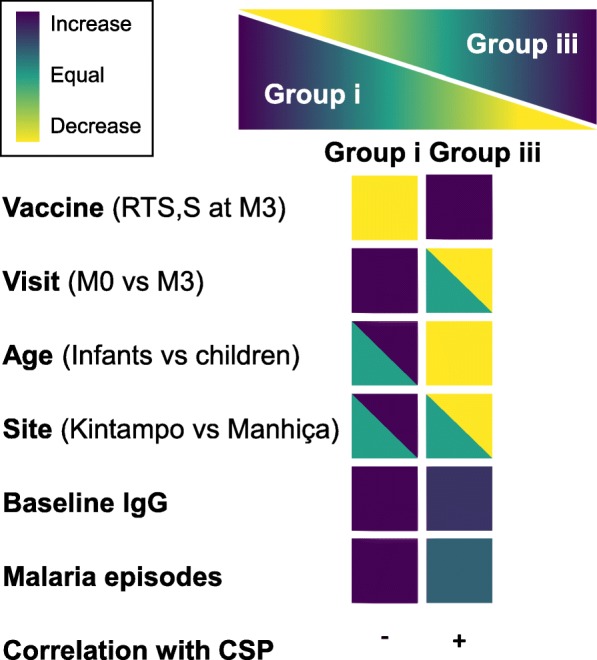
Summary chart of antibody responses to group i and group iii antigens

A schematic summary of the characteristics of antibody responses to group i vs iii antigens is shown in Fig. 7. Antigens to which there were more maternally derived antibodies at M0 and increased with previous malaria at M3 (and by M0 levels), and for which levels were higher in Kintampo than in Manhiça (group i), were the ones that did not increase with RTS,S vaccination at M3, and vice versa. Table 2 shows a summary of the analyses of correlates with malaria risk and protection.

**Table 2:**
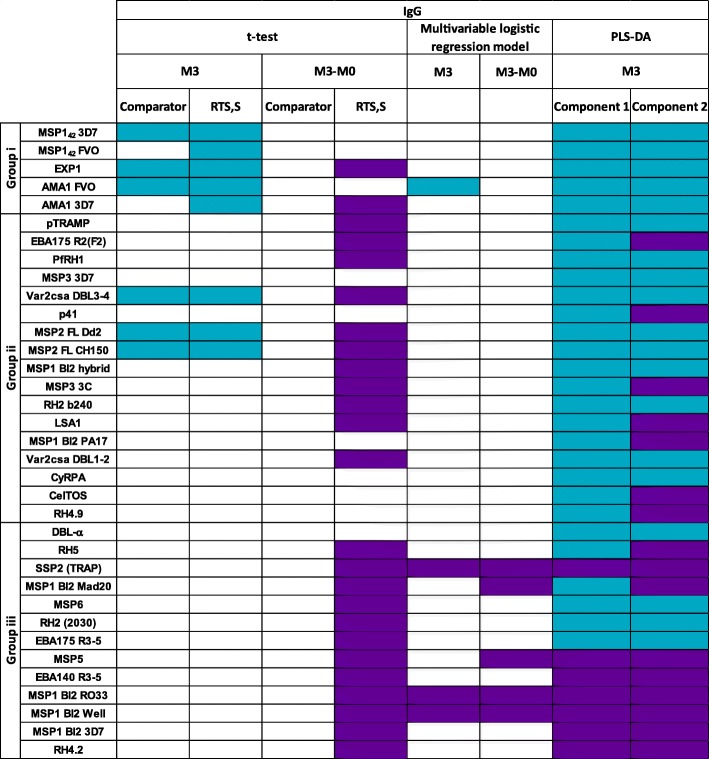
Summary table of associations between IgG responses and malaria protection. Dark purple represents association with protection and light blue with risk

## Discussion

Immunization with a partially effective PE malaria vaccine, RTS,S/AS01E, may affect the acquisition of IgG and IgM responses to multiple PE and BS *P. falciparum* antigens upon natural microbial exposure in young African hosts, some of whom have maternal IgGs. Our results reveal an association between these antibody levels and protection against clinical malaria, in two areas of different endemicity. Data from a phase 3 clinical trial showed that RTS,S vaccination can alter NAI antibodies to *P. falciparum* in two different ways: by decreasing antibody levels (“group i” antigens), as seen in prior phase 2b trials [12, 13, 24], or by increasing IgG levels (“group iii” antigens), which to our knowledge has not been reported before, likely due to the limitations of previous studies in their breadth of antibody screen and time points available for study. This study had the additional advantage of being able to draw on the association between antibodies to NAI antigenic targets and protection from, or risk of, clinical malaria measured prospective in a controlled multicenter trial.

On the one hand, RTS,S vaccination decreases exposure to the parasite by providing protection against malaria infection and this would be reflected in the reduction of antibody levels to *P. falciparum* antigens that are primarily markers of microbial exposure, even if functional antibody activity exists. This effect would be more distinct with time, as seen in prior phase 2b studies by Campo et al. 6 months after vaccination (M8.5 visit), particularly in younger children [12, 13]. In our phase 3 study, group i antigens that are considered and used as markers of MTI in seroepidemiological surveys [15] showed this pattern, specifically MSP1_42_ and AMA1. Notably, as early as 1 month post-vaccination (M3), there were some significant differences in antibody levels as a result of reduced *P. falciparum* BS exposure associated with vaccine efficacy. Antibodies to MSP1_42_, in particular, appear to be very fast and sensitive sensors to detect changes in parasite exposure. We predict that with time, antibody levels to most group ii antigens would also be reduced as a result of RTS,S vaccination, similar to the Campo et al. studies. Antibodies to group i and ii antigens behave as markers of exposure based on multiple criteria: (a) they are significantly higher in Kintampo (higher MTI) than in Manhiça (lower MTI), (b) increase significantly if the subject had prior malaria episodes, (c) M3 levels strongly correlate with M0 levels, and (d) point toward an increased malaria risk (AMA1). Furthermore, IgG levels for many of those antigens are elevated at baseline, higher in infants than in children, and significantly decay from M0 to M3, indicating a predominantly maternal origin. Transplacental antibodies at birth are also a reflection of MTI in the community, and higher levels at baseline are associated with prospective malaria risk. IgG to some antigens like AMA1 and VAR2CSA DBL1–2 and DBL3–4 appear to be longer-lived maternal antibodies, as they remain higher in infants than in children also at M3.

On the other hand, our data also show that vaccination with RTS,S can result in an increase in antibody levels to other antigens (group iii) with respect to comparators and baseline. Stratified by age, the effect of increased antibody levels at M3 by RTS,S (not comparator) vaccination is manifested in both infants and children but appears more pronounced in children. This could be because children have more exposure cumulatively and no maternal IgG remaining at M3 that could interfere with the build-up of their own antibodies. It could also be that children are able to produce stronger responses just because of intrinsic age-related particularities of the immune system, such as maturation of T-dependent B cell responses [25]. Interestingly, upon RTS,S vaccination, IgG levels to group iii antigens increased in both sites, Manhiça and Kintampo; however, Manhiça had a greater increase post-vaccination than Kintampo, perhaps representing complex interactions between host immune response and levels of parasite exposure. Remarkably, the M0 to M3 antibody kinetics and the analysis of factors affecting the Ig levels suggest that group iii antigens are weaker markers of exposure than group i and ii antigens and that infants are not born with high IgG to them. It could be that antibodies to these antigens might be transferred less transplacentally (due to antigen-driven polarization of subclasses or glycoprofiles affecting binding to FcRn) or that mothers have lower levels. Thus, adults could have lower IgG levels to group iii antigens than children, i.e., an age pattern such that antibodies increase in early infancy and childhood but at some point decrease and are lower in adults, contrary to group i antigens. For some antigens like EBA140, this is consistent with prior analyses of RNA expression in parasites that decreased with age [26]. The different age pattern of group iii antibodies (e.g., EBA140) points to different roles in anti-disease vs anti-parasite immunity, which may be acting at different phases of NAI acquisition. Therefore, it could be that the antibody “enhancement” seen with RTS,S for group iii antigens may be related mostly to the absence/low levels of maternally derived IgGs to these antigens such that host responses are not interfered with, in contrast to group i and ii antigens. Overall, there seems to be an inverse relationship between group i + ii (“exposure”) vs group iii antigens (see opposite patterns in Fig. 7 and Additional file 1: Table S2 and Figure S2). Interestingly, antibody levels are heterogeneous within antigen groups, as different behaviors are observed upon close examination that imply that the determinants and patterns of antibody responses to each individual antigen are more complex and diverse than these three categories. For example, when IgG to group i and ii antigens are stratified by age, IgG responses to EXP1 and MSP2 in RTS,S-vaccinated children (who do not have maternal antibodies at M3) resemble those of group iii antigens.

How could a vaccine that reduces exposure to the parasite be associated with an increase in antibody levels? This could be related to the fact that RTS,S may not result in sterile immunity, particularly in the longer term. Instead, it appears to be a partially effective or “leaky” PE vaccine. To explain the differences in duration of vaccine efficacy between two cohorts subject to diverse MTI in the Mozambican phase 2b trial, we hypothesized that partial protection afforded by RTS,S/AS0 may stimulate protective antibodies to certain asexual BS target antigens, through a reduction in merozoite release from the liver, leading to attenuated BS parasitemia. As CSP-specific highly protective immunity decays in the short term [27–30], incoming infections would be partially controlled resulting in subpatent low antigen doses that elicit enhanced antibody production to certain antigens. This would be reflected in accelerated acquisition of BS protective immunity [10, 12, 29]. As this is an exposure-dependent mechanism, the outcome will differ depending on the force of infection to which subjects are exposed, i.e., MTI- and age-dependence. Therefore, a certain MTI window (enough but not too much) may maximize benefits of RTS,S vaccination and baseline factors (e.g., frequency/intensity of parasite exposure, presence of maternal antibodies) may modulate non-RTS,S antigen responses affected by the vaccine. This would imply that the potential positive impact of immunization with RTS,S or other leaky vaccines may not be apparent in sites with too low MTI and that it might be lost in sites with too high MTI, which may overwhelm the initial required protective effect in the liver stage. It is remarkable that this antibody enhancement phenomenon can occur in the short 3-month interval of our study (during vaccination and 1 month post-vaccination). The concept of immunologically favorable impact of low-level infections is not new, as a similar explanation was proposed for the prolonged protective effect of intermittent preventive treatment in infants with sulfadoxine-pyrimethamine (IPTi-SP) in Tanzania upon interruption of treatment [31]. Thus, induction of effective and sustained immunity against malaria by IPTi (or RTS,S) could be due to the generation of low-dose blood-stage inocula and attenuated infections [32], and this could translate into higher antibody responses [33]. Additionally, insecticide-treated bednet use can also enhance antibodies [34] and ultra-low dose of BS infection can protect [11], although residual antimalarial concentration before challenge compromised these results, and protection was not thought to be antibody-mediated. Furthermore, not only BS but also PE antibodies like anti-SSP2 IgG and IgM increased. Conceivable, this could be an independent mechanism whereby RTS,S-induced CSP antibodies [7] recognizing parasites may increase phagocytocis of sporozoites which leads to presentation of other PE antigens to the immune system and consequent antibody responses. The fact that RTS,S affects non-vaccine antibody responses would suggest an association between antibody levels to CSP and group iii antigens, as shown in the PCA and correlation analyses here. At least at the level of primary structure, there is little evidence that RTS,S-induced CSP antibodies cross-react with group iii antigens with the alignment strategy used here. This does not preclude the possibility of tertiary structure or other undetected similarities between any of the antigens tested with CSP, but does suggest that the group iii antigens identified in this analysis represent targets of specific antibodies affected by RTS,S vaccination.

What is the immunological relevance of RTS,S-mediated changes in non-vaccine antibodies on malaria protective immunity? Antibodies to group i and ii antigens were generally associated with increased malaria risk, consistent with their role as markers for identifying individuals under the highest attack rates. There is little dispute that antibodies to exposure antigens such as AMA1 can protect against malaria, particularly allele-specific infection [35], but these effects are often confounded in immunoepidemiological studies by the strong effect of prior exposure on both antibodies and future risk of repeated infection [36]. In contrast, antibodies to group iii antigens seem to have a clearer association with NAI in our study. Among group iii antigens, the most consistent associations between M3 antibody levels and immunity were for MSP1 Bl2 [RO33, Well, 3D7], MSP5, EBA140, RH4.2, and SSP2 antigens. Antibody responses to MSP1 Bl2 [37], MSP5 [38, 39], EBA140 [40], and RH4.2 [41, 42] BS antigens have been previously associated with protection against malaria, and SSP2 (TRAP) is the target of other leading subunit PE vaccine candidates [43]. Functionally, antibodies against erythrocyte binding antigens, including EBA140 [44], and against the reticulocyte binding protein-like homolog family, particularly RH4 and RH5 [45], have shown in vitro inhibition of erythrocyte invasion. These parasite proteins play critical roles as ligands for host erythrocytes and the process of merozoite invasion. It follows that RTS,S enhancement of such antibodies could provide an additional layer of protective immunity on the back end of an infection. The functional relevance of MSP5 antibodies is less clear, although antibodies to MSP5 orthologues have been protective in animal models [46, 47]. Interestingly, MSP5 has high levels of expression in *P. falciparum* sporozoites (PlasmoDB.org; [48]), and MSP5 antibodies correlate with the PE sterile protection observed in humans immunized with irradiated sporozoites (Campo et al., in preparation), perhaps hinting at a multistage function of MSP5 antibodies. Under certain circumstances (e.g., in children with low baseline Ig), antibody responses to EXP1 and MSP2 and other antigens may also have a role in protection.

What is the net impact of the interaction between RTS,S immunization and NAI on overall protection against malaria? On one hand, there is a potential negative effect whereby RTS,S vaccination, by providing protection against malaria infection, might reduce NAI that would normally be induced by repeated infections. This could render vaccinated children more susceptible when the vaccine effect wanes [49] if such antibodies were mediators of NAI, and potentially lead to malaria “rebound” [50]. However, it appears that antibodies that decrease following RTS,S vaccination are mostly markers of exposure that reflect vaccine efficacy [12, 13, 24]; thus, this may not be a concern. On the other hand, there is a potential positive impact whereby NAI could improve overall vaccine efficacy in the capacity of RTS,S to protect against malaria, combining the effect of experimentally elicited and NAI-dependent immunity, and this may be age- and/or exposure-dependent. Malaria prevention in early life when the infant is at highest risk of the negative effects of infection is thought to contribute to an overall lower risk of severe morbidity and thus mortality in childhood [51]. Varying endemicity may differentially influence the long-term morbidity and mortality risk so that the benefits of RTS,S vaccination may be maximized in moderate MTI settings. In the pediatric field trials of RTS,S, there was evidence of different vaccine efficacies depending on malaria surveillance system and MTI [29], although in the phase 3 trial differences due to MTI were only significant in children when administered with a vaccine booster dose [49]. The long-term impact of the interaction between RTS,S vaccination and NAI on duration of vaccine efficacy and overall protection against malaria will be investigated in follow-up studies including the subsequent trial visits at M20, M21, and M32 till study end. We speculate that antibodies altered by RTS,S vaccination will affect the longevity of protective immunity in an age- and MTI-dependent manner.

Importantly, our study provides evidence for a positive effect of RTS,S on antibody responses to certain antigens that are associated with protection. Since multivariable logistic regression models in which IgGs to MSP1 Bl2, MSP5, and SSP2 (that are increased by vaccination) were associated with protection were adjusted by RTS,S vaccination, there is an additional protective effect of these antibodies on top of the protection afforded by the RTS,S vaccine. We postulate that these responses are contributing to the RTS,S-induced malaria protection and that these antigens could improve efficacy in a future multivalent next-generation vaccine. Such formulations raise the possibility of saturation of the immune system, having to react simultaneously to multiple antigens. However, there are vaccines given through the expanded program of immunization (EPI) that contain more than 3 antigens and are able to induce adequate immune responses. Moreover, it is envisaged that in the pilot implementation starting 2019 in three African countries, RTS,S will not be administered to infants as part of the EPI but to children age 5–17 months; thus, immune saturation may not represent a significant limitation.

The study design has some limitations that could be addressed in follow-up studies including more sites and larger sample size. In Kintampo, all children who fulfilled the inclusion criteria, most of whom had malaria, were analyzed. Whereas in Manhiça, a case-control design was needed because there were fewer cases; a cohort design would have required increasing the number of samples substantially to have power to detect association with protection. In Manhiça, there was also an age imbalance, as most cases were in infants. In addition, the phase 3 trial design did not allow for collection of genetics data from the volunteers. Therefore, host genetics-based differences in the immune responses to vaccination between the two African populations, including hemoglobinopathies such as the sickle cell trait, could not be assessed here. Despite this and having many antigens and multiple comparisons, patterns emerged that were consistent and biologically plausible.

## Conclusion

Baseline characteristics related to age and exposure appear to modulate the nature and magnitude of antibody responses to distinct sets of *P. falciparum* antigens induced by the interaction between vaccination and NAI. Importantly, antibodies to certain antigens which may be targets of protective NAI may be enhanced by RTS,S/AS01E vaccination, contributing to vaccine efficacy. These antigens could potentially be candidates of multivalent next-generation RTS,S formulations to improve vaccine efficacy as additive (or synergistic) responses with CSP may occur. We postulate that antibodies affected by RTS,S/AS01E vaccination at peak response will contribute to the duration of protection. We plan to test this hypothesis in the extended follow-up of the phase 3 trial.

## Additional file

Additional file 1: Supplementary information including figures, tables, methods, results, and references. **Figure S1.** Antibody responses to non-RTS,S *Plasmodium falciparum* antigens per visit and vaccination group. **Figure S2.** Antibody responses to non-RTS,S *Plasmodium falciparum* antigens after RTS,S/AS01E vaccination stratified by age and site. **Figure S3.** Association between IgG antibody levels and malaria risk stratified by age and site. **Table S1.** Antigens included in the multiplex quantitative suspension array panel. **Table S2.** Effect of RTS,S vaccination and other variables on antibody levels at each study visit by univariable linear regression models. **Table S4.** Multivariable analysis of the effect of RTS,S/AS01E vaccination and demographic, clinical, and epidemiological variables on antibody levels to *Plasmodium falciparum* pre-erythrocytic and blood-stage antigens. (DOCX 1736 kb)

## Data Availability

All data generated or analyzed during this study are included in this article and its supplementary information files or are available from the authors on request.

## Abbreviations

a.a.: Amino acid
AIC: Akaike information criterion
AUC: Area under the curve
Bl: Block
BS: Blood stage
BSA: Bovine serum albumin
CI: Confidence interval
CSP: Circumsporozoite protein
FC: Fold change
GAM: Generalized additive models
GST: Glutathione S*-*transferase
HAZ: Height-for-age *Z*-score
IPTi-SP: Intermittent preventive treatment in infants with sulfadoxine-pyrimethamine
IQR: Interquartile ranges
MFI: Median fluorescence intensity
MTI: Malaria transmission intensity
NAI: Naturally acquired immunity
OR: Odds ratio
PCA: Principal component analysis
PCD: Passive case detection
PE: Pre-erythrocytic
PLS-DA: Partial least squares discriminant analysis
qSAT: Quantitative suspension array technology
WAZ: Weight-for-age *Z*-score
