# Supplementary material for: RTS,S/AS01E immunization increases antibody responses to vaccine-unrelated Plasmodium falciparum antigens associated with protection against clinical malaria in African children: a case-control study
Source: BMC Med. 2019 Aug 14;17:157. doi: 10.1186/s12916-019-1378-6 (PMC6693200; doi:10.1186/s12916-019-1378-6)
Supplement: Supplementary file 1 — Supplementary information including figures, tables, methods, results, and references. Figure S1. Antibody responses to non-RTS,S Plasmodium falciparum antigens per visit and vaccination group. Figure S2. Antibody responses to non-RTS,S Plasmodium falciparum antigens after RTS,S/AS01E vaccination stratified by age and site. Figure S3. Association between IgG antibody levels and malaria risk stratified by age and site. Table S1. Antigens included in the multiplex quantitative suspension array panel. Table S2. Effect of RTS,S vaccination and other variables on antibody levels at each study visit by univariable linear regression models. Table S4. Multivariable analysis of the effect of RTS,S/AS01E vaccination and demographic, clinical, and epidemiological variables on antibody levels to Plasmodium falciparum pre-erythrocytic and blood-stage antigens. (DOCX 1736 kb) [file 12916_2019_1378_MOESM1_ESM.docx]

**SUPPLEMENTARY INFORMATION**

**SUPPLEMENTARY TABLES AND FIGURES**

**Supplementary Fig 1. Antibody responses to non-RTS,S *Plasmodium falciparum* antigens per visit and vaccination group.** Representative examples.

**a) IgG:** group ii antigens that do not change upon RTS,S vaccination


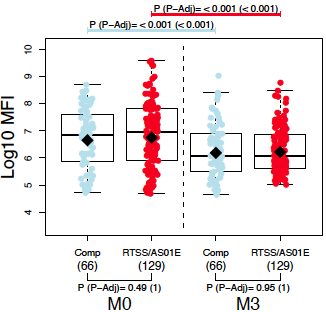

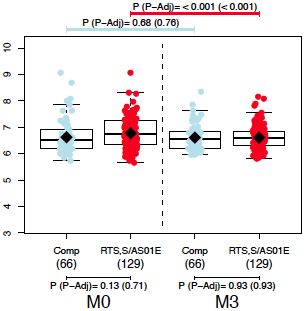

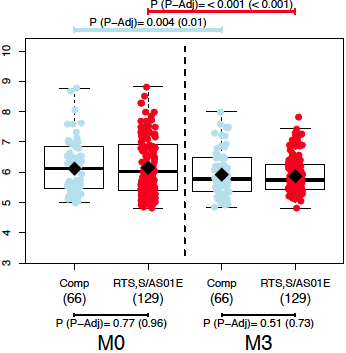


**MSP2 Dd2**

**EBA175 R2F2**

**P41**


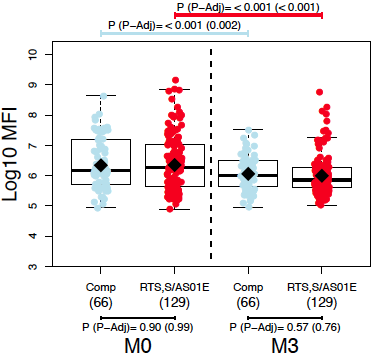

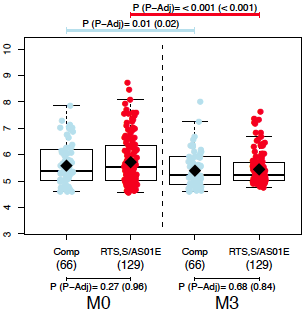

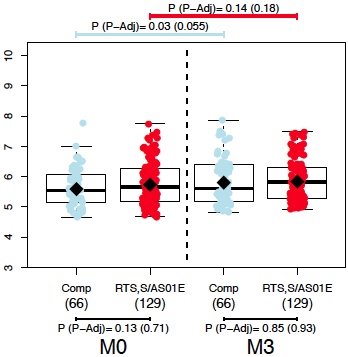


**MSP3 3D7**

**LSA1**

**MSP3 3C**


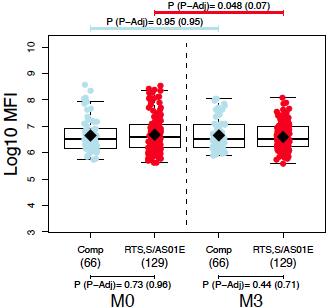

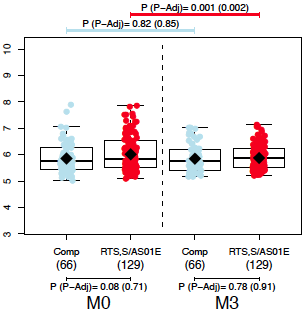

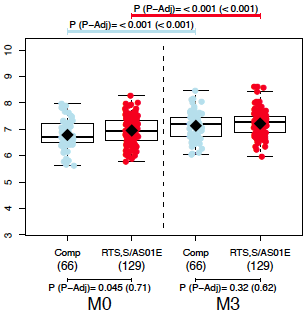


**Rh2 b240**

**Rh1**

**Rh4.9**

**b) IgM:** antibody responses mostly do not change upon RTS,S vaccination except MSP1_42_ 3D7 that follows the pattern of group i antigens (decrease)

**AMA1 3D7 MSP2 Dd2 MSP1_42_ 3D7**


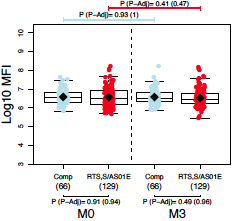

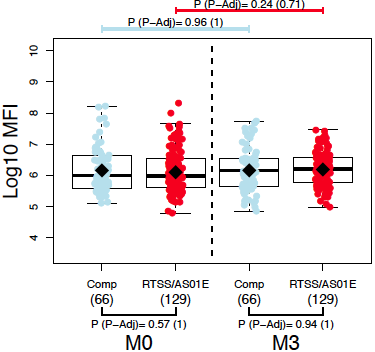

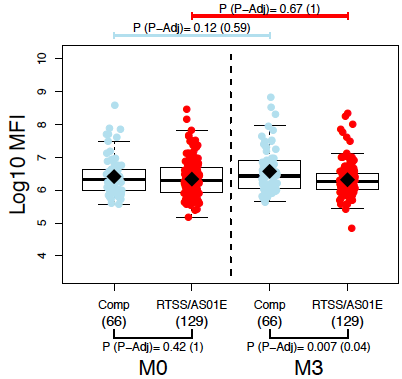


**EBA140 MSP5 MSP1 Bl2 3D7**


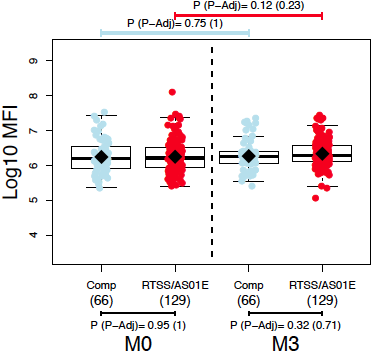

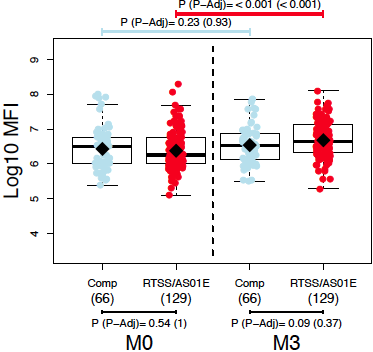

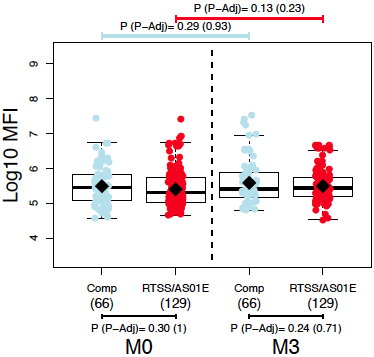


**Supplementary Fig 2. Antibody responses to non-RTS,S *Plasmodium falciparum* antigens after RTS,S/AS01E vaccination stratified by age and site.** Crude Ig levels (log_10_ MFI).

**a) By age**

**Group i**


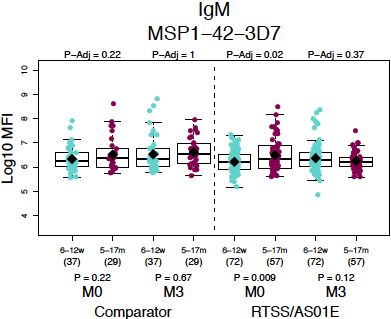

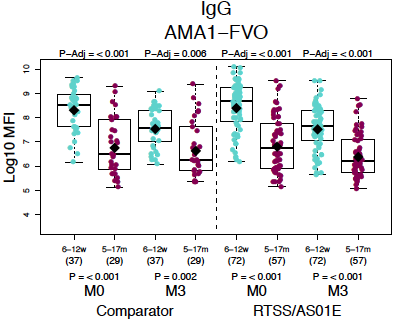

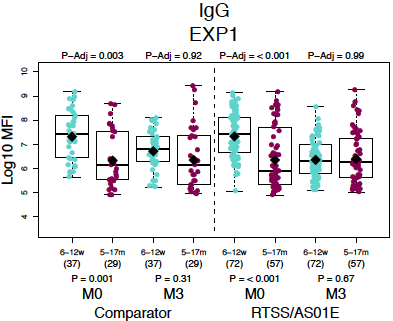


**Group iii**


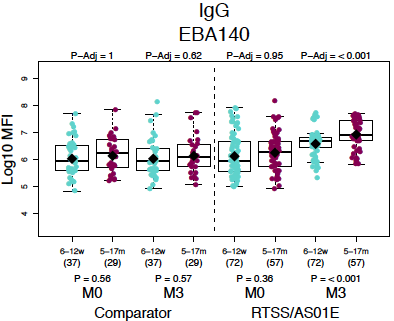


**b) By site**

**Group i**


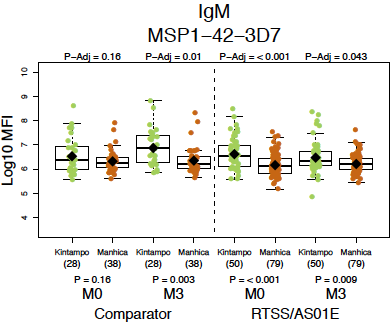

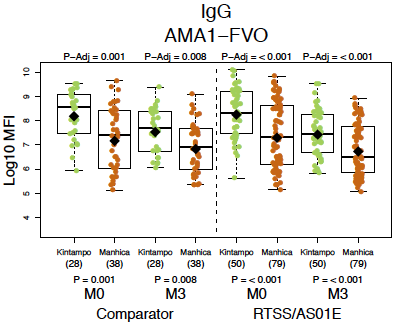


**Group iii**


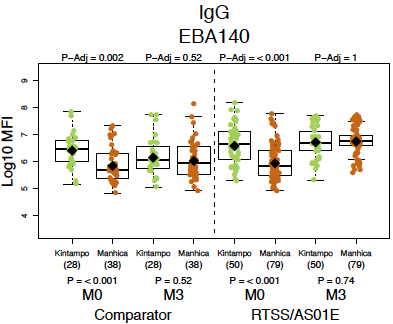


**c) By age and by site.** All are IgG except MSP1_42_ that is IgM. K = Kintampo, M = Manhiça.

**Group i**


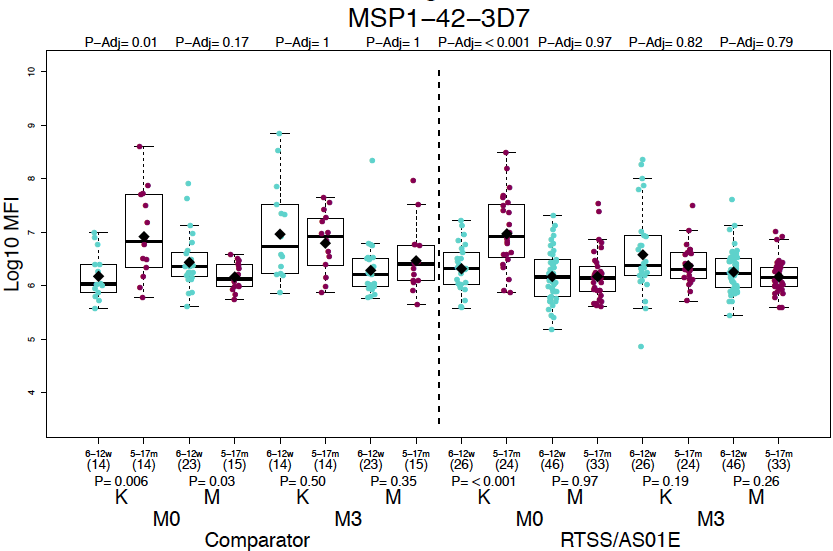

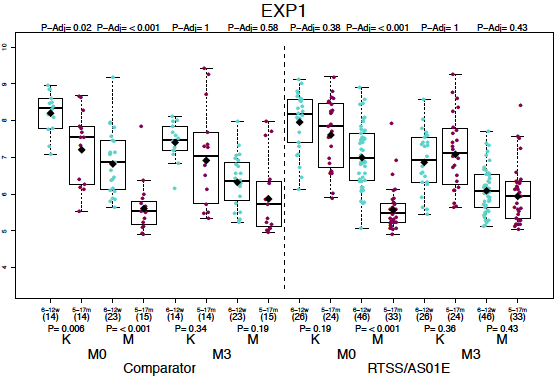


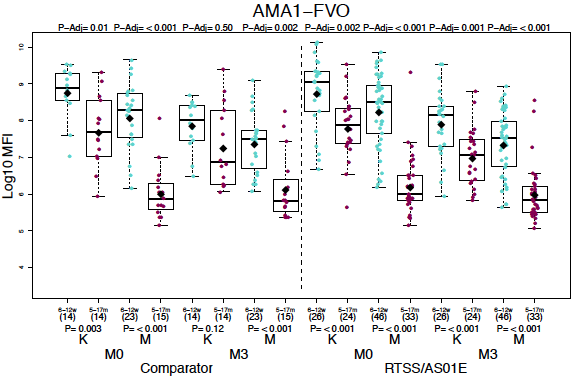


**Group ii**


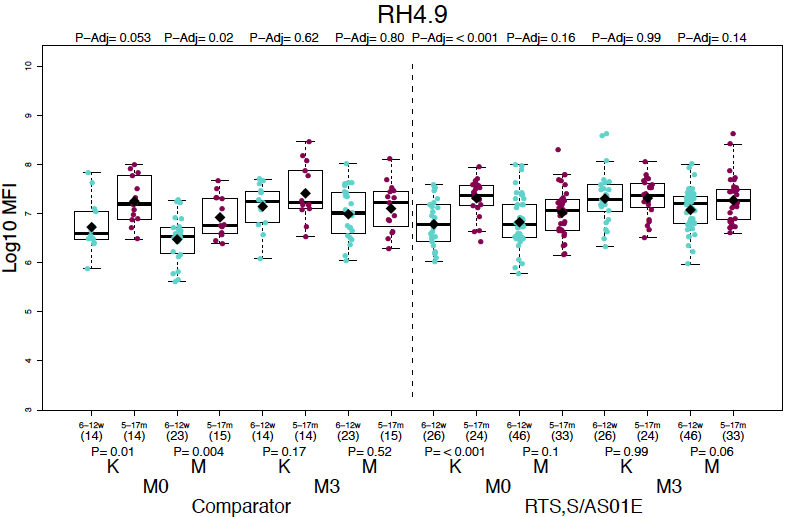


**Group iii**


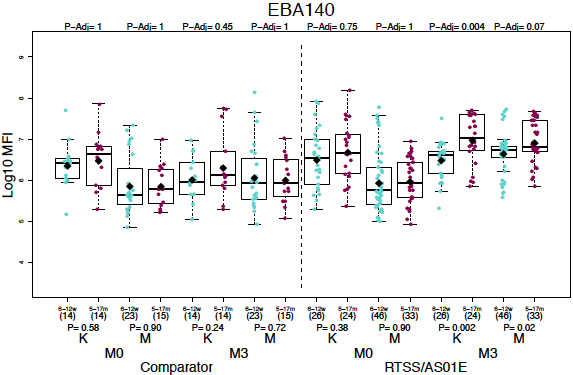

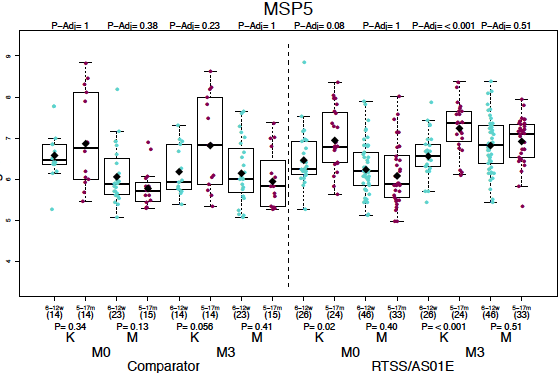


**Supplementary Fig 3. Association between IgG antibody levels and malaria risk stratified by age and site.** M = Malaria *vs* NM = No malaria. K = Kintampo *vs* M = Manhiça.

**AGE SITE**

**a) Group i antigens**

**MSP1_42_**

**
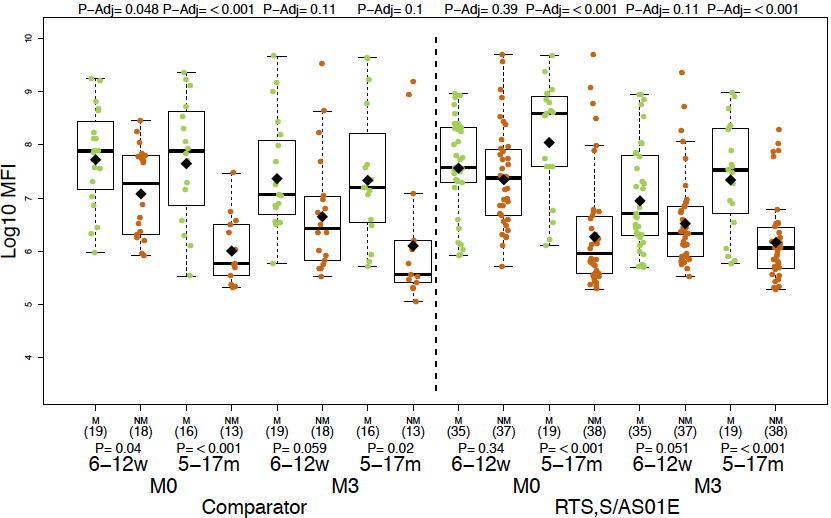
** **
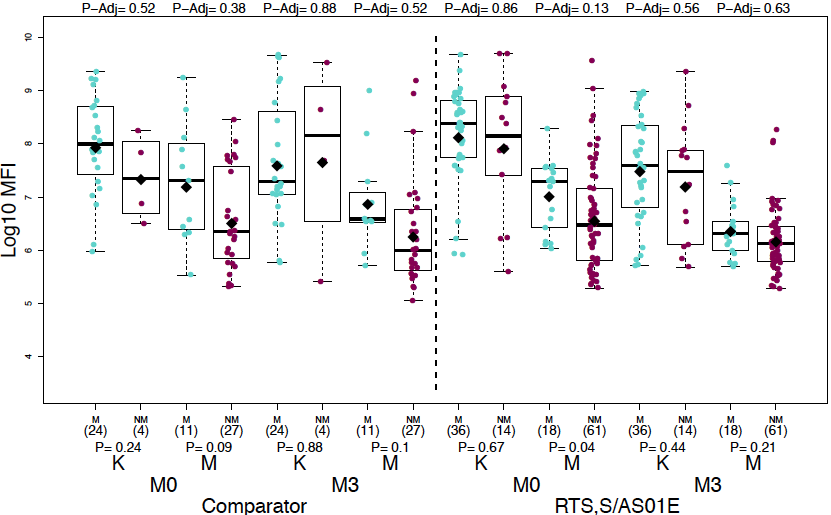
**

**AMA1**

**
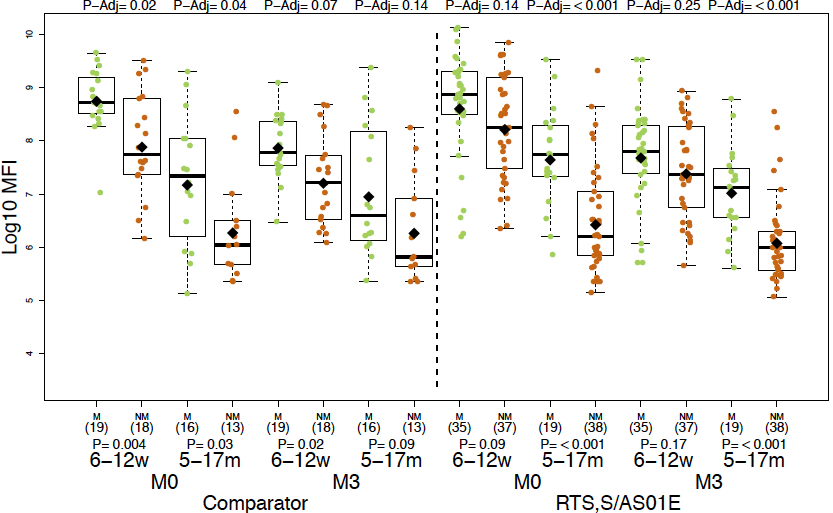

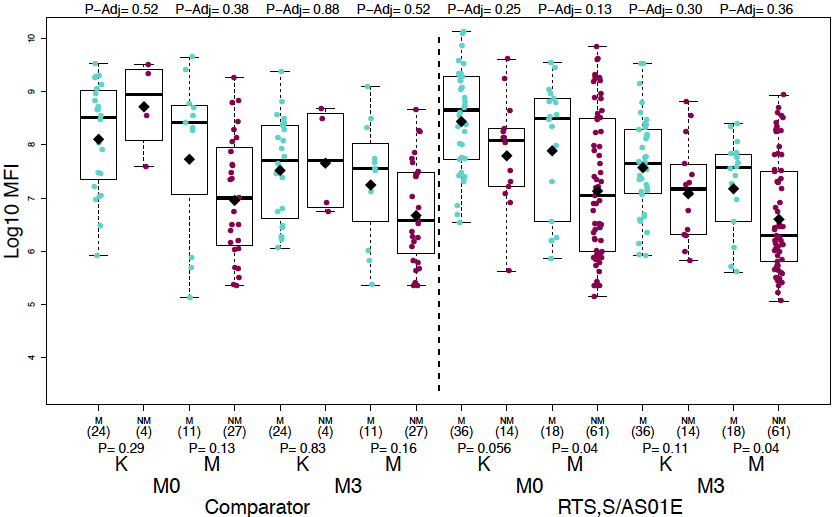
**

**EXP1**

**
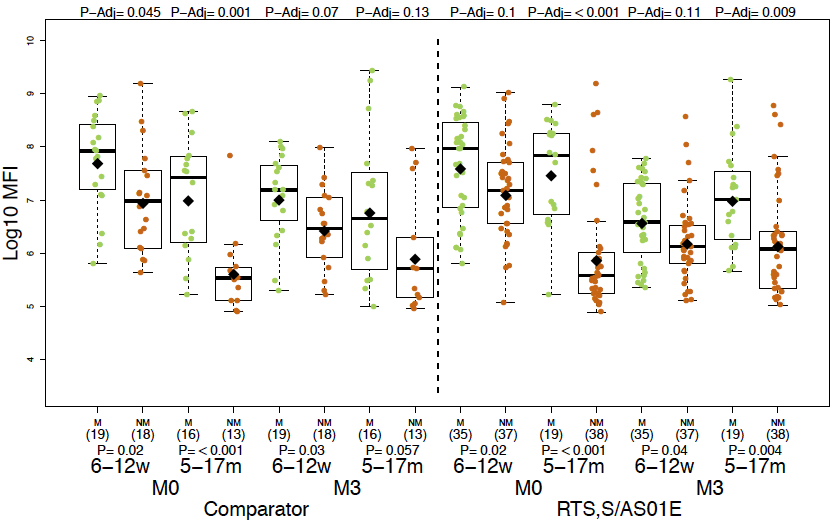

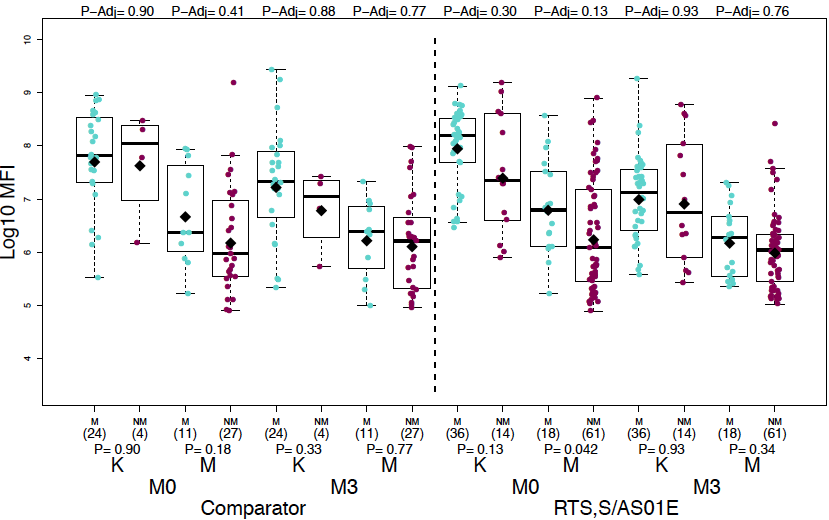
**

**AGE SITE**

**b) Group ii antigens**

**MSP2 CH150**


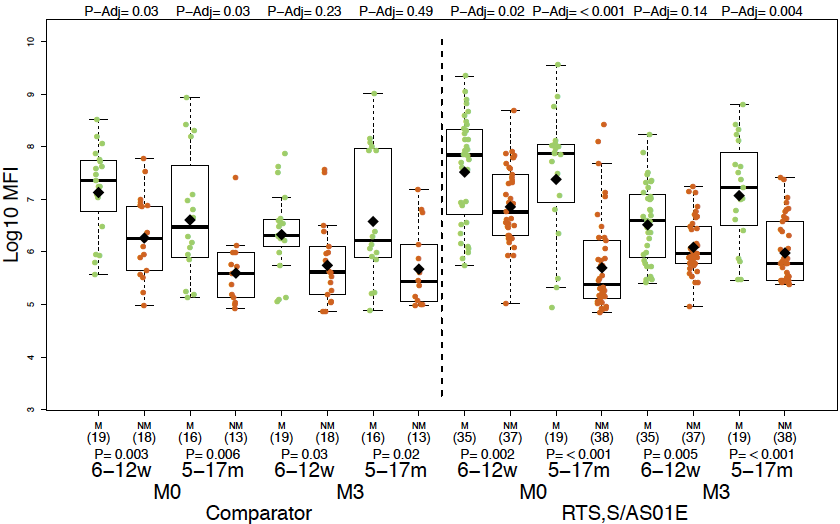

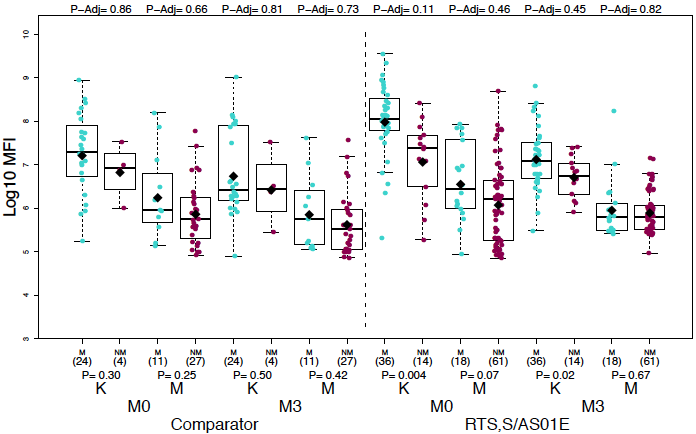


**Rh4.9**

**
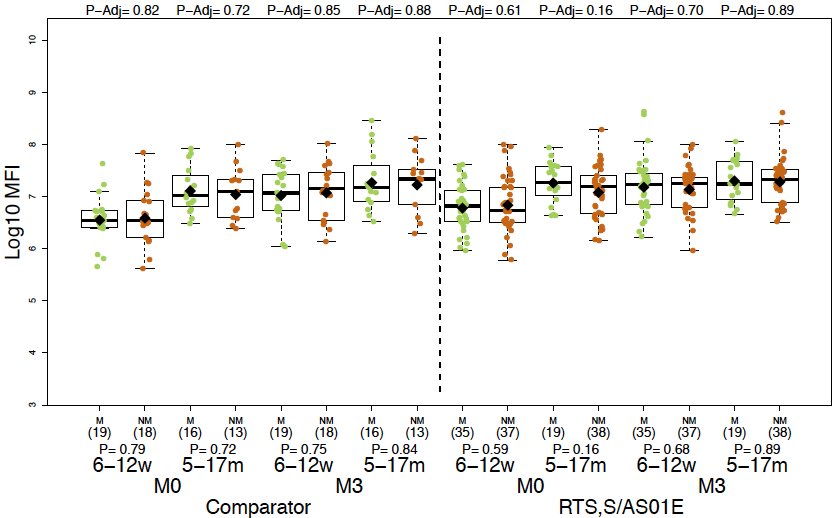

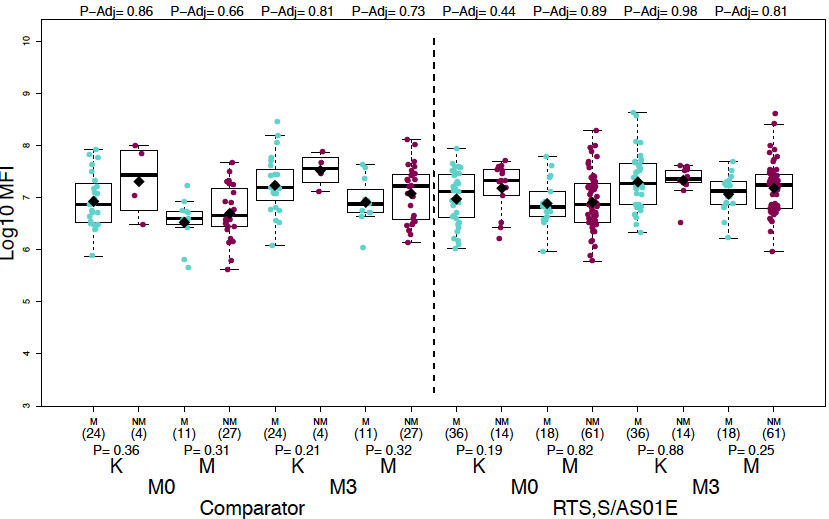
**

**c) Group iii antigens**

**EBA140**


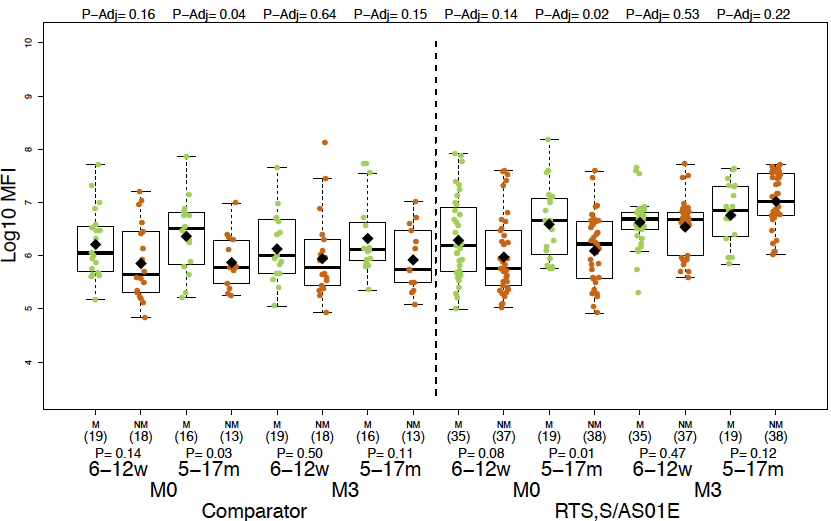

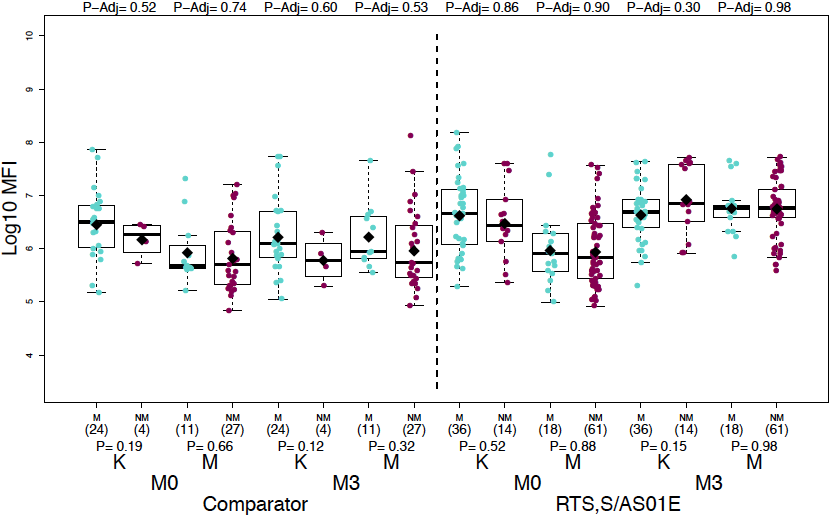


**MSP5**

**
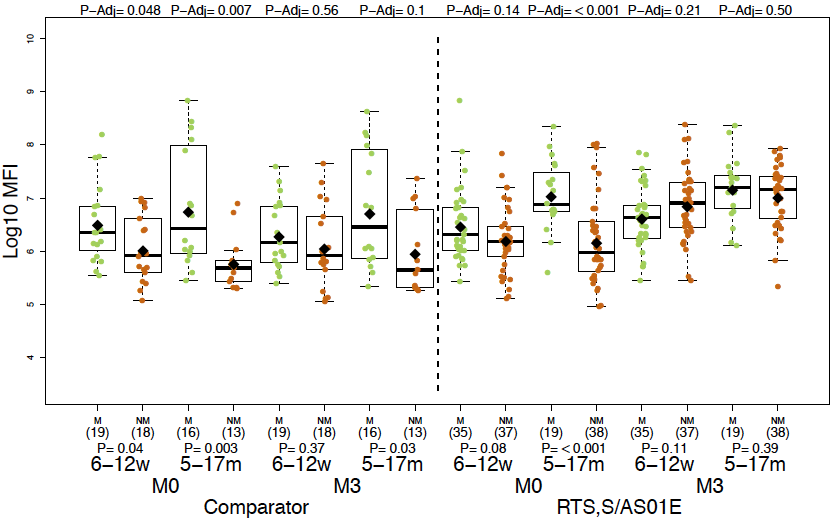
** **
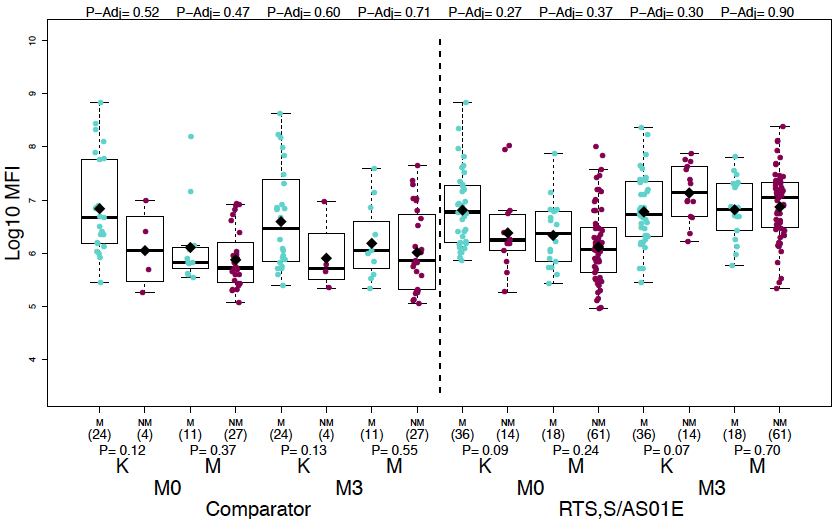
**

**Table S1. Antigens included in the multiplex quantitative suspension array panel**

| **Antigen** | **Rationale** | **Malaria life stage** | **Expression** | **Reference** |
| --- | --- | --- | --- | --- |
| CSP full length | Component of RTS,S vaccine | Sporozoite |  | [1,2] |
| CSP NANP repeat |  |  | GST fusion | [3] |
| CSP C-terminus |  |  | GST fusion | [4] |
| SSP2 or TRAP | Exposure to sporozoite infection |  |  | [5,6] |
| CelTOS |  |  |  | [7,8] |
| LSA1 |  | Liver |  | [9,10] |
| EXP1 | Exposure to asexual blood stage infection |  |  | [11] |
| AMA1 3D7 (FMP2.1) |  | Merozoite |  | [12,13] |
| AMA1 FVO (FMP009) |  |  |  | [12] |
| CyRPA full length |  |  |  | [14] |
| EBA140 region 3-5 |  |  | GST fusion | [15] |
| EBA175 region 2 PfF2 |  |  |  | [16] |
| EBA175 region 3-5 |  |  | GST fusion | [15] |
| MSP1_42_ 3D7 |  |  |  | [12,17] |
| MSP1_42_ FVO |  |  |  | [12,17] |
| MSP1 Block 2 PA17 |  |  | GST fusion | [18] |
| MSP1 Block 2 3D7 |  |  | GST fusion | [18] |
| MSP1 Block 2 MAD20 |  |  | GST fusion | [18] |
| MSP1 Block 2 RO33 |  |  | GST fusion | [18] |
| MSP1 Block 2 Wellcome |  |  | GST fusion | [18] |
| MSP1 Block 2 hybrid |  |  | GST fusion | [19] |
| MSP2 CH150 full length (5/6) Type A |  |  | GST fusion | [20] |
| MSP2 Dd2 full length (13/14) Type B |  |  | GST fusion | [20] |
| MSP3 3C |  |  |  | [20] |
| MSP3 3D7 |  |  |  | [21] |
| MSP5 |  |  |  | [22,23] |
| MSP6 |  |  | GST fusion | [24] |
| PTRAMP |  |  |  | [25] |
| P41 |  |  |  | [26] |
| RH1 |  |  |  | [27] |
| RH2 b240 |  |  |  | [28] |
| RH2 (2030) |  |  | GST fusion | [29] |
| RH4.2 |  |  | GST fusion | [30,31] |
| RH4.9 |  |  |  | [30,31] |
| RH5 |  |  |  | [14,32] |
| DBLα |  | Trophozoite |  | [33] |
| VAR2CSA DBL1-2 | Representative of maternally-transferred antibodies |  |  | [34] |
| VAR2CSA DBL3-4 |  |  |  | [35] |

**Table S2. Effect of different variables on month 3 IgG levels to *Plasmodium falciparum* antigens.**

| **Antibody** | **Vaccine (RTS,S)** | | **Age (5-17 months)** | | **Site (Manhiça)** | | **Baseline IgG levels** | | **Malaria episodes** | |
| --- | --- | --- | --- | --- | --- | --- | --- | --- | --- | --- |
| **Antigen** | **Coefficient** | **P** | **Coefficient** | **P** | **Coefficient** | **P** | **Coefficient** | **P** | **Coefficient** | **P** |
| **Group i antigens** | | | | | | | | | | |
| MSP1_42_ 3D7 | **0.58 (0.37;0.91)** | **0.02** |  |  | **0.56 (0.33;0.96)** | **0.03** | **3.42 (2.74;4.27)** | **< 0.001** | **15.66 (7.51;32.65)** | **< 0.001** |
| MSP1_42_ FVO | 0.62 (0.36;1.05) | 0.08 |  |  | **0.38 (0.2;0.72)** | **0.003** | **2.76 (2.17;3.51)** | **< 0.001** | **19.4 (8.09;46.48)** | **< 0.001** |
| EXP1 | 0.65 (0.42;1.01) | 0.054 | **3.5 (2.17;5.64)** | **< 0.001** |  |  | **4.16 (3.4;5.09)** | **< 0.001** | **3.69 (1.82;7.47)** | **< 0.001** |
| AMA1 FVO | 0.7 (0.48;1.02) | 0.06 |  |  | 1.44 (0.95;2.18) | 0.08 | **4.79 (4.15;5.54)** | **< 0.001** | **6.37 (3.45;11.75)** | **< 0.001** |
| AMA1 3D7 | **0.65 (0.45;0.93)** | **0.02** |  |  | 1.33 (0.89;1.99) | 0.16 | **4.87 (4.25;5.58)** | **< 0.001** | **4.69 (2.63;8.36)** | **< 0.001** |
| **Group ii antigens** | | | | | | | | | | |
| pTRAMP |  |  |  |  |  |  | **2.78 (2.22;3.48)** | **< 0.001** | **3.02 (1.84;4.97)** | **< 0.001** |
| EBA175 R2(F2) |  |  | **1.49 (1.12;1.99)** | **0.006** | 1.28 (0.93;1.77) | 0.13 | **3.29 (2.79;3.88)** | **< 0.001** | **3.64 (2.28;5.82)** | **< 0.001** |
| PfRH1 |  |  | **1.6 (1.2;2.13)** | **0.001** |  |  | **2.72 (2.19;3.38)** | **< 0.001** | **2.06 (1.3;3.26)** | **0.002** |
| MSP3 3D7 |  |  | **2.45 (1.76;3.42)** | **< 0.001** | **1.45 (1.02;2.05)** | **0.04** | **4.19 (3.44;5.1)** | **< 0.001** | **2.62 (1.61;4.26)** | **< 0.001** |
| Var2csa DBL3-4 |  |  | 0.83 (0.65;1.07) | 0.14 |  |  | **3.01 (2.66;3.41)** | **< 0.001** | **1.57 (1.11;2.22)** | **0.01** |
| p41 |  |  | **1.34 (1.06;1.68)** | **0.01** |  |  | **2.79 (2.32;3.37)** | **< 0.001** | **1.88 (1.31;2.71)** | **< 0.001** |
| MSP2 FL Dd2 |  |  | **3.89 (2.56;5.9)** | **< 0.001** | 1.48 (0.91;2.41) | 0.12 | **4.72 (3.79;5.88)** | **< 0.001** | **4.52 (2.56;7.98)** | **< 0.001** |
| MSP2 FL CH150 |  |  | **2.95 (2.06;4.22)** | **< 0.001** |  |  | **3.55 (3.04;4.15)** | **< 0.001** | **6.37 (3.68;11.02)** | **< 0.001** |
| MSP1 Bl2 hybrid |  |  | **1.58 (1.14;2.19)** | **0.006** | 1.33 (0.91;1.96) | 0.14 | **2.98 (2.51;3.54)** | **< 0.001** | **4.67 (2.71;8.06)** | **< 0.001** |
| MSP3 3C |  |  | 0.65 (0.41;1.03) | 0.07 | **2.06 (1.25;3.41)** | **0.005** | **2.87 (2.04;4.04)** | **< 0.001** | **2.05 (0.97;4.34)** | 0.059 |
| PfRH2 b240 |  |  | **1.5 (1.18;1.9)** | **0.001** |  |  | **2.41 (2;2.92)** | **< 0.001** | **3.16 (2.13;4.68)** | **< 0.001** |
| LSA1 |  |  | **1.6 (1.15;2.23)** | **0.006** | **1.48 (1;2.19)** | **0.05** | **2.99 (2.43;3.67)** | **< 0.001** | **2.29 (1.31;4)** | **0.004** |
| MSP1 Bl2 PA17 |  |  |  |  |  |  | **2.27 (1.88;2.74)** | **< 0.001** | **2.54 (1.52;4.25)** | **< 0.001** |
| Var2csa DBL1-2 |  |  |  |  |  |  | **2.64 (2.18;3.2)** | **< 0.001** |  |  |
| CyRPA |  |  |  |  |  |  | **3.43 (2.69;4.39)** | **< 0.001** |  |  |
| CelTOS |  |  |  |  |  |  | **2.41 (1.85;3.14)** | **< 0.001** | **1.72 (1.07;2.78)** | **0.03** |
| RH4.9 |  |  |  |  |  |  | **3.31 (2.58;4.24)** | **< 0.001** | **1.81 (1.19;2.74)** | **0.006** |
| **Group iii antigens** | | | | | | | | | | |
| DBL-α | 1.29 (0.95;1.75) | 0.1 | **1.77 (1.33;2.37)** | **< 0.001** |  |  | **2.74 (2.1;3.58)** | **< 0.001** |  |  |
| RH5 | **1.52 (1.08;2.14)** | **0.02** | **1.61 (1.16;2.23)** | **0.005** |  |  | **1.97 (1.5;2.58)** | **< 0.001** | **1.93 (1.13;3.29)** | **0.02** |
| SSP2 (TRAP) | **1.65 (1.2;2.28)** | **0.003** | **3.51 (2.55;4.83)** | **< 0.001** | **1.65 (1.16;2.34)** | **0.006** | **3.79 (2.87;5)** | **< 0.001** | **2.65 (1.56;4.5)** | **< 0.001** |
| MSP1 Bl2 Mad20 | **1.9 (1.64;2.2)** | **<0.001** | **1.3 (1.13;1.49)** | **< 0.001** | **1.19 (1.01;1.4)** | **0.03** | **2 (1.64;2.42)** | **< 0.001** | **1.44 (1.15;1.81)** | **0.002** |
| MSP6 | **1.91 (1.39;2.62)** | **<0.001** | **1.82 (1.35;2.45)** | **< 0.001** |  |  | **2.51 (2.06;3.06)** | **< 0.001** | **2.39 (1.46;3.92)** | **< 0.001** |
| RH2 (2030) | **2.18 (1.63;2.92)** | **<0.001** | **1.61 (1.14;2.27)** | **0.007** | **1.47 (1.04;2.06)** | **0.03** | **2.51 (2.01;3.13)** | **< 0.001** | **3.27 (2.01;5.33)** | **< 0.001** |
| EBA175 R3-5 | **3.67 (2.41;5.61)** | **<0.001** | **2.06 (1.35;3.15)** | **< 0.001** |  |  | **2.14 (1.69;2.7)** | **< 0.001** | 1.61 (0.83;3.1) | 0.16 |
| MSP5 | **3.52 (2.18;5.68)** | **<0.001** | 1.5 (0.95;2.38) | 0.08 | 1.6 (0.95;2.67) | 0.08 | **1.89 (1.4;2.54)** | **< 0.001** | **3.68 (1.74;7.8)** | **< 0.001** |
| EBA140 R3-5 | **4.01 (2.71;5.93)** | **<0.001** | **1.6 (1.09;2.34)** | **0.02** | **1.61 (1.03;2.52)** | **0.04** | **1.8 (1.36;2.38)** | **< 0.001** | 1.66 (0.87;3.16) | 0.12 |
| MSP1 Bl2 RO33 | **5.16 (3.66;7.28)** | **<0.001** | **1.51 (1.07;2.11)** | **0.02** |  |  | **1.72 (1.34;2.2)** | **< 0.001** | **2.45 (1.43;4.21)** | **0.001** |
| MSP1 Bl2 Well | **5.83 (4.34;7.82)** | **<0.001** | **1.78 (1.32;2.4)** | **< 0.001** | **1.53 (1.12;2.09)** | **0.008** | **2.58 (1.93;3.46)** | **< 0.001** | 1.51 (0.93;2.44) | 0.09 |
| MSP1 Bl2 3D7 | **6.84 (4.58;10.2)** | **<0.001** | **1.53 (1.04;2.26)** | **0.03** |  |  | **1.78 (1.38;2.29)** | **< 0.001** | 1.8 (0.96;3.36) | 0.07 |
| RH4.2 | **6.55 (4.51;9.51)** | **<0.001** | **2.15 (1.5;3.1)** | **< 0.001** | 1.49 (0.99;2.24) | 0.054 | **1.96 (1.45;2.66)** | **< 0.001** | **2.05 (1.11;3.78)** | **0.02** |

Each line shows multivariable linear regressions results with antibody levels for each antigen (log_10_ Median Fluorescence Intensity measured by quantitative suspension array technology) as outcome and vaccine, age, site, baseline (month 0, M0) IgG levels and malaria episodes from M0 to M3 as predictor covariables (in parenthesis the category that is compared to the reference). Coefficients (10^Beta) with 95% confidence intervals and p-values are shown for variables retained in the models according to the minimum akaike information criterion**.** The order of the antigens is based on coefficients and p-values of the predictor variable Vaccine. Month 0 data are shown in Table S4. Associations for the following variables were only significant for a few antigens and are not included in the table: season (pTRAMP, 2.65, p=0.004; MSP1 Bl2 Mad20, 1.54, p=0.007), hemoglobin (MSP1 Bl2 Well, 0.87, p=0.01), weight-for-age Z-score (MSP2 FL [full-length] CH150, 0.86, p=0.046) height-for-age Z-score (AMA1 3D7, 0.83, p=0.01; P41, 1.17, p=0.01; CyRPA, 1.13, p=0.01; RH2 2030, 0.82, p=0.02). Sex was retained in the models for MSP3 3C, CyRPA, MSP1 Bl2 PA17 and SSP2 but it was not significantly associated with antibody levels. Bl2: block 2.

**Table S3.** Effect of RTS,S vaccination and other variables (columns) on antibody levels (log_10_MFI) at each study visit by univariable linear regression models (in parenthesis the category that is compared to the reference). Coefficients (10^Beta), 95% confidence intervals and p values (significant values in bold) are shown. Antigens are ordered according to the RTS,S vaccination coefficient values (lower to higher) of their IgG responses, and this is used to define the three pattern groups (i, ii, iii) presented in this study.

**a)** Antibody levels at month 3 for IgG and IgM.

| **IgG** | | | | | | | | | | | | |
| --- | --- | --- | --- | --- | --- | --- | --- | --- | --- | --- | --- | --- |
| **Antigen** | **Vaccine (RTS,S)** | | **Age (5-17 month)** | | **Site (Manhiça)** | | **Season (Low)** | | **Baseline IgG levels** | | **Previous malaria** | |
|  | **Coefficient** | **P** | **Coefficient** | **P** | **Coefficient** | **P** | **Coefficient** | **P** | **Coefficient** | **P** | **Coefficient** | **P** |
| **Group i antigens** | | | | | | | | | | | | |
| MSP1_42_ 3D7 | 0.51 (0.24;1.08) | 0.21 | 0.65 (0.32;1.34) | 0.37 | **0.06 (0.03;0.11)** | **<0.001** | 0.7 (0.15;3.34) | 0.72 | **4.87 (3.93;6.03)** | **< 0.001** | **89.36 (33.92;235.44)** | **< 0.001** |
| MSP1_42_ FVO | 0.53 (0.24;1.18) | 0.3 | 0.97 (0.45;2.09) | 0.93 | **0.06 (0.03;0.11)** | **<0.001** | 0.55 (0.1;2.88) | 0.69 | **4.15 (3.27;5.26)** | **< 0.001** | **104.33 (36.57;297.66)** | **< 0.001** |
| EXP1 | 0.63 (0.33;1.24) | 0.4 | 0.89 (0.47;1.69) | 0.82 | **0.1 (0.06;0.18)** | **<0.001** | 0.4 (0.1;1.57) | 0.41 | **3.72 (3.07;4.51)** | **< 0.001** | **20.67 (8.14;52.48)** | **< 0.001** |
| AMA1 FVO | 0.74 (0.36;1.55) | 0.62 | **0.09 (0.05;0.17)** | **< 0.001** | **0.21 (0.1;0.4)** | **<0.001** | 1.61 (0.35;7.27) | 0.71 | **4.92 (4.25;5.69)** | **< 0.001** | **10.83 (3.7;31.67)** | **< 0.001** |
| AMA1 3D7 | 0.77 (0.37;1.59) | 0.64 | **0.1 (0.05;0.18)** | **< 0.001** | **0.2 (0.1;0.39)** | **<0.001** | 1.37 (0.31;6.08) | 0.72 | **4.89 (4.27;5.61)** | **< 0.001** | **7.51 (2.57;21.96)** | **< 0.001** |
| **Group ii antigens** | | | | | | | | | | | | |
| pTRAMP | 0.78 (0.52;1.17) | 0.46 | 1.22 (0.83;1.8) | 0.43 | **0.53 (0.36;0.79)** | **0.003** | 1.86 (0.81;4.3) | 0.36 | **3.05 (2.44;3.82)** | **< 0.001** | **4.52 (2.51;8.14)** | **< 0.001** |
| EBA175 R2 F2 | 0.85 (0.54;1.34) | 0.64 | 0.94 (0.61;1.45) | 0.82 | **0.38 (0.25;0.58)** | **<0.001** | 0.58 (0.23;1.45) | 0.45 | **3.24 (2.77;3.79)** | **< 0.001** | **6.69 (3.54;12.63)** | **< 0.001** |
| RH1 | 0.86 (0.59;1.25) | 0.62 | **2.11 (1.5;2.98)** | **< 0.001** | **0.39 (0.28;0.55)** | **<0.001** | 0.8 (0.37;1.73) | 0.71 | **3.16 (2.57;3.89)** | **< 0.001** | **4.11 (2.39;7.06)** | **< 0.001** |
| MSP3 3D7 | 0.88 (0.56;1.39) | 0.74 | 0.79 (0.51;1.21) | 0.4 | **0.4 (0.26;0.61)** | **<0.001** | 0.84 (0.33;2.13) | 0.73 | **3.2 (2.69;3.8)** | **< 0.001** | **3.26 (1.67;6.37)** | **0.001** |
| VAR2CSA DBL3-4 | 0.96 (0.63;1.46) | 0.94 | **0.27 (0.19;0.38)** | **< 0.001** | **0.34 (0.24;0.5)** | **<0.001** | 0.73 (0.31;1.71) | 0.69 | **3.23 (2.92;3.59)** | **< 0.001** | **3.01 (1.62;5.59)** | **< 0.001** |
| p41 | 0.99 (0.72;1.35) | 0.95 | **1.53 (1.14;2.06)** | **0.01** | **0.59 (0.44;0.8)** | **0.001** | 0.67 (0.35;1.29) | 0.45 | **2.97 (2.46;3.58)** | **< 0.001** | **3.29 (2.09;5.17)** | **< 0.001** |
| MSP2 FL Dd2 | 1.02 (0.55;1.89) | 0.95 | 0.91 (0.51;1.65) | 0.82 | **0.1 (0.06;0.16)** | **<0.001** | 0.39 (0.11;1.37) | 0.36 | **3.8 (3.22;4.48)** | **< 0.001** | **22.32 (9.71;51.3)** | **< 0.001** |
| MSP1 Bl2 hybrid | 1.03 (0.63;1.69) | 0.95 | 1.08 (0.67;1.73) | 0.82 | **0.33 (0.21;0.52)** | **<0.001** | 0.45 (0.16;1.23) | 0.36 | **3.02 (2.56;3.55)** | **< 0.001** | **7.71 (3.84;15.49)** | **< 0.001** |
| MSP3 3C | 1.05 (0.64;1.73) | 0.94 | 1.06 (0.66;1.7) | 0.84 | 1.06 (0.65;1.71) | 0.85 | 0.76 (0.28;2.12) | 0.71 | **2.4 (1.74;3.3)** | **< 0.001** | 1.97 (0.93;4.19) | 0.08 |
| RH2 b240 | 1.05 (0.75;1.48) | 0.89 | **1.51 (1.1;2.08)** | **0.02** | **0.46 (0.34;0.63)** | **<0.001** | 0.62 (0.31;1.24) | 0.41 | **2.76 (2.26;3.38)** | **< 0.001** | **5.23 (3.3;8.27)** | **< 0.001** |
| LSA1 | 1.1 (0.7;1.73) | 0.81 | 1.2 (0.78;1.84) | 0.53 | **0.46 (0.3;0.71)** | **<0.001** | 0.65 (0.26;1.63) | 0.57 | **2.83 (2.35;3.4)** | **< 0.001** | **3.65 (1.89;7.08)** | **< 0.001** |
| MSP1 Bl2 PA17 | 1.13 (0.75;1.7) | 0.71 | 1.11 (0.75;1.64) | 0.72 | **0.61 (0.41;0.9)** | **0.02** | 0.64 (0.28;1.47) | 0.5 | **2.44 (2.02;2.96)** | **< 0.001** | **4.07 (2.26;7.35)** | **< 0.001** |
| VAR2CSA DBL1-2 | 1.15 (0.84;1.58) | 0.58 | **0.59 (0.44;0.78)** | **0.001** | **0.61 (0.45;0.82)** | **0.002** | 0.87 (0.46;1.66) | 0.72 | **2.57 (2.13;3.1)** | **< 0.001** | 1.61 (1.01;2.59) | 0.055 |
| CYRPA | 1.16 (0.88;1.55) | 0.53 | **1.71 (1.32;2.21)** | **< 0.001** | 0.85 (0.65;1.12) | 0.28 | 1.01 (0.56;1.8) | 0.99 | **3.38 (2.64;4.33)** | **< 0.001** | 1.56 (1.02;2.4) | 0.05 |
| CelTOS | 1.18 (0.83;1.67) | 0.58 | **1.64 (1.19;2.27)** | **0.008** | **0.69 (0.5;0.96)** | **0.045** | 0.51 (0.25;1.03) | 0.26 | **2.56 (1.97;3.32)** | **< 0.001** | **2.35 (1.4;3.94)** | **0.002** |
| RH4.9 | 1.19 (0.85;1.67) | 0.53 | 1.43 (1.04;1.96) | 0.051 | **0.66 (0.48;0.9)** | **0.02** | 0.81 (0.41;1.62) | 0.71 | **3.46 (2.7;4.43)** | **< 0.001** | **2.38 (1.44;3.91)** | **0.001** |
| **Group iii antigens** | | | | | | | | | | | | |
| DBL-α | 1.23 (0.86;1.75) | 0.48 | **1.85 (1.34;2.56)** | **< 0.001** | 0.73 (0.52;1.02) | 0.09 | 0.79 (0.39;1.64) | 0.71 | **2.77 (2.11;3.64)** | **< 0.001** | **1.79 (1.05;3.05)** | **0.04** |
| MSP2 FL CH150 | 1.55 (0.85;2.82) | 0.35 | 1.24 (0.7;2.21) | 0.57 | **0.08 (0.05;0.14)** | **<0.001** | 0.29 (0.09;1) | 0.25 | **3.44 (2.89;4.08)** | **< 0.001** | **20.66 (9.17;46.53)** | **< 0.001** |
| RH5 | 1.57 (1.08;2.29) | 0.057 | **1.92 (1.35;2.73)** | **0.001** | **0.56 (0.39;0.81)** | **0.003** | 0.4 (0.18;0.86) | 0.17 | **2.37 (1.81;3.09)** | **< 0.001** | **2.76 (1.57;4.86)** | **< 0.001** |
| SSP2 (TRAP) | 1.68 (1.09;2.59) | 0.057 | **3.11 (2.12;4.57)** | **< 0.001** | 0.77 (0.5;1.17) | 0.26 | 0.57 (0.23;1.39) | 0.44 | **3.49 (2.55;4.76)** | **< 0.001** | **3.14 (1.64;6)** | **0.001** |
| MSP1 Bl2 MAD20 | **1.75 (1.49;2.06)** | **< 0.001** | 1.17 (0.98;1.38) | 0.14 | 0.91 (0.76;1.08) | 0.31 | 0.91 (0.63;1.31) | 0.71 | **1.75 (1.42;2.16)** | **< 0.001** | **1.44 (1.1;1.88)** | **0.01** |
| MSP6 | **2.01 (1.35;3.01)** | **0.003** | **1.7 (1.15;2.51)** | **0.02** | **0.43 (0.3;0.64)** | **<0.001** | 0.48 (0.2;1.11) | 0.3 | **2.78 (2.26;3.42)** | **< 0.001** | **4.2 (2.3;7.66)** | **< 0.001** |
| EBA175 R3-5 | **2.14 (1.47;3.1)** | **<0.001** | 0.82 (0.57;1.18) | 0.4 | **0.55 (0.39;0.8)** | **0.003** | 0.49 (0.22;1.08) | 0.29 | **2.33 (1.92;2.82)** | **< 0.001** | **4.15 (2.39;7.23)** | **< 0.001** |
| RH2 2030 | **3.85 (2.41;6.15)** | **< 0.001** | 1.38 (0.85;2.23) | 0.31 | 0.66 (0.41;1.08) | 0.14 | 0.45 (0.16;1.25) | 0.36 | **2.01 (1.58;2.56)** | **< 0.001** | **2.38 (1.11;5.1)** | **0.03** |
| MSP5 | **3.95 (2.4;6.5)** | **<0.001** | **1.96 (1.19;3.24)** | **0.02** | 0.71 (0.43;1.19) | 0.25 | **0.17 (0.06;0.5)** | **0.047** | **2.04 (1.51;2.76)** | **< 0.001** | **3.4 (1.53;7.54)** | **0.004** |
| EBA140 R3-5 | **4.32 (2.86;6.54)** | **< 0.001** | **1.87 (1.21;2.87)** | **0.01** | 1.01 (0.65;1.58) | 0.97 | 0.61 (0.23;1.56) | 0.5 | **1.84 (1.38;2.46)** | **< 0.001** | 1.64 (0.81;3.31) | 0.17 |
| MSP1 Bl2 RO33 | **5.17 (3.54;7.56)** | **< 0.001** | **1.72 (1.13;2.61)** | **0.02** | 0.7 (0.46;1.08) | 0.14 | 0.39 (0.16;0.96) | 0.23 | **2.12 (1.59;2.82)** | **< 0.001** | **2.9 (1.49;5.63)** | **0.003** |
| MSP1 Bl2 Well | **5.71 (4.03;8.09)** | **< 0.001** | **2.52 (1.71;3.7)** | **< 0.001** | 1.1 (0.73;1.66) | 0.69 | 0.29 (0.12;0.68) | 0.08 | **2.94 (2.02;4.26)** | **< 0.001** | 1.12 (0.58;2.15) | 0.74 |
| MSP1 Bl2 3D7 | **6.78 (4.45;10.34)** | **< 0.001** | 1.34 (0.83;2.15) | 0.37 | 0.68 (0.42;1.1) | 0.15 | 0.31 (0.11;0.85) | 0.17 | **1.82 (1.36;2.45)** | **< 0.001** | 2.14 (1;4.57) | 0.056 |
| RH4.2 | **7.19 (4.74;10.9)** | **<0.001** | **2.55 (1.61;4.03)** | **< 0.001** | 0.89 (0.55;1.45) | 0.69 | 0.27 (0.1;0.74) | 0.13 | **2.34 (1.64;3.33)** | **< 0.001** | 1.98 (0.93;4.24) | 0.08 |

No statistical significant differences were detected for sex, weight for age Z-score, height for age Z-score or hemoglobin (data not shown).

| **IgM** | | | | | | | | | | | | |
| --- | --- | --- | --- | --- | --- | --- | --- | --- | --- | --- | --- | --- |
| **Antigen** | **Age (5-17 month)** | | **Site (Manhiça)** | | **HAZ** | | **Season (Low)** | | **Baseline IgM levels** | | **Previous malaria** | |
|  | **Coefficient** | **P** | **Coefficient** | **P** | **Coefficient** | **P** | **Coefficient** | **P** | **Coefficient** | **P** | **Coefficient** | **P** |
| **Group i antigens** | | | | | | | | | | | | |
| MSP1_42_ 3D7 | 0.86 (0.58;1.28) | 0.56 | **0.42 (0.29;0.61)** | **<0.001** | 0.87 (0.73;1.03) | 0.19 | 0.54 (0.23;1.26) | 0.22 | 1.3 (0.94;1.8) | 0.11 | **4.74 (2.62;8.61)** | **<0.001** |
| MSP1_42_ FVO | 1.53 (1.03;2.28) | 0.09 | **0.42 (0.29;0.62)** | **< 0.001** | 0.83 (0.7;0.98) | 0.1 | **0.35 (0.15;0.81)** | **0.041** | **2.02 (1.46;2.81)** | **<0.001** | **4.36 (2.37;8.01)** | **<0.001** |
| EXP1 | **2.76 (1.68;4.51)** | **<0.001** | **0.39 (0.24;0.65)** | **< 0.001** | 0.96 (0.77;1.2) | 0.71 | **0.26 (0.09;0.77)** | **0.041** | **2.31 (1.64;3.24)** | **<0.001** | **9.91 (4.66;21.1)** | **<0.001** |
| AMA1 FVO | 0.85 (0.64;1.14) | 0.4 | **0.62 (0.46;0.82)** | **0.002** | 0.89 (0.78;1) | 0.13 | 0.69 (0.37;1.28) | 0.32 | **1.73 (1.27;2.34)** | **<0.001** | **2.2 (1.4;3.46)** | **0.001** |
| AMA1 3D7 | 0.88 (0.65;1.19) | 0.52 | **0.55 (0.41;0.74)** | **< 0.001** | 0.91 (0.8;1.04) | 0.24 | 0.89 (0.46;1.72) | 0.73 | **1.68 (1.23;2.29)** | **0.001** | **2.59 (1.62;4.14)** | **<0.001** |
| **Group ii antigens** | | | | | | | | | | | | |
| pTRAMP | 0.9 (0.71;1.14) | 0.51 | **0.64 (0.5;0.81)** | **<0.001** | **0.82 (0.74;0.91)** | **0.006** | 0.87 (0.52;1.46) | 0.62 | **2.11 (1.51;2.93)** | **< 0.001** | **1.79 (1.23;2.62)** | **0.004** |
| EBA175 R2 F2 | 1.37 (0.98;1.9) | 0.14 | **0.5 (0.36;0.69)** | **< 0.001** | 0.9 (0.78;1.03) | 0.21 | 0.52 (0.25;1.05) | 0.12 | **2.07 (1.51;2.84)** | **<0.001** | **2.52 (1.51;4.22)** | **0.001** |
| RH1 | 1.21 (0.93;1.56) | 0.28 | **0.64 (0.5;0.82)** | **0.001** | 0.92 (0.83;1.03) | 0.24 | 0.62 (0.35;1.07) | 0.14 | **2.38 (1.81;3.15)** | **<0.001** | **2.14 (1.43;3.19)** | **<0.001** |
| MSP3 3D7 | 1.06 (0.78;1.46) | 0.77 | **0.7 (0.51;0.96)** | **0.03** | 0.89 (0.78;1.02) | 0.19 | 0.55 (0.28;1.08) | 0.14 | **1.9 (1.43;2.51)** | **<0.001** | 1.51 (0.91;2.49) | 0.11 |
| VAR2CSA DBL3-4 | 0.98 (0.76;1.25) | 0.87 | **0.63 (0.5;0.81)** | **< 0.001** | **0.84 (0.76;0.94)** | **0.02** | 0.74 (0.43;1.26) | 0.35 | **1.98 (1.46;2.67)** | **<0.001** | **1.7 (1.14;2.52)** | **0.01** |
| p41 | 0.99 (0.75;1.31) | 0.93 | **0.63 (0.48;0.83)** | **0.002** | 0.88 (0.78;1) | 0.12 | 0.86 (0.47;1.57) | 0.63 | **2.1 (1.54;2.87)** | **<0.001** | **2.09 (1.35;3.23)** | **0.002** |
| MSP2 FL Dd2 | **2.1 (1.43;3.09)** | **0.001** | **0.46 (0.31;0.69)** | **< 0.001** | 0.83 (0.7;0.99) | 0.1 | **0.18 (0.08;0.41)** | **< 0.001** | **2.04 (1.57;2.65)** | **<0.001** | **3.54 (1.91;6.56)** | **<0.001** |
| MSP1 Bl2 hybrid | **1.77 (1.2;2.63)** | **0.02** | 0.74 (0.49;1.11) | 0.14 | 0.93 (0.78;1.1) | 0.44 | **0.33 (0.14;0.77)** | **0.04** | **1.8 (1.36;2.39)** | **<0.001** | **4.36 (2.37;8.01)** | **<0.001** |
| MSP3 3C | 0.91 (0.61;1.37) | 0.75 | **0.64 (0.42;0.96)** | **0.03** | 0.89 (0.74;1.06) | 0.24 | 0.66 (0.28;1.59) | 0.41 | **2.22 (1.68;2.95)** | **<0.001** | **2.48 (1.31;4.71)** | **0.008** |
| RH2 b240 | 1.36 (0.99;1.86) | 0.14 | **0.61 (0.44;0.83)** | **0.004** | **0.83 (0.72;0.95)** | **0.042** | **0.41 (0.21;0.79)** | **0.04** | **2.15 (1.61;2.89)** | **<0.001** | **2.28 (1.39;3.74)** | **0.002** |
| LSA1 | 1.66 (1.06;2.6) | 0.08 | **0.27 (0.17;0.4)** | **< 0.001** | 0.89 (0.73;1.08) | 0.26 | 0.61 (0.23;1.61) | 0.38 | **2.05 (1.55;2.7)** | **<0.001** | **4.5 (2.25;8.99)** | **<0.001** |
| MSP1 Bl2 PA17 | 1.5 (0.96;2.33) | 0.15 | **0.53 (0.34;0.82)** | **0.007** | 0.88 (0.73;1.07) | 0.24 | 0.35 (0.14;0.91) | 0.07 | **1.82 (1.3;2.53)** | **<0.001** | **5.73 (2.92;11.23)** | **<0.001** |
| VAR2CSA DBL1-2 | 1.09 (0.77;1.53) | 0.73 | **0.53 (0.38;0.75)** | **< 0.001** | 0.84 (0.73;0.97) | 0.08 | 0.44 (0.21;0.91) | 0.06 | **1.76 (1.27;2.42)** | **<0.001** | 1.62 (0.94;2.8) | 0.08 |
| CYRPA | 0.8 (0.56;1.14) | 0.33 | **0.39 (0.28;0.55)** | **< 0.001** | **0.77 (0.66;0.89)** | **0.009** | 0.76 (0.35;1.63) | 0.52 | **2.58 (1.9;3.51)** | **<0.001** | **2.32 (1.33;4.05)** | **0.005** |
| CelTOS | **1.57 (1.15;2.15)** | **0.02** | **0.63 (0.46;0.87)** | **0.007** | 0.92 (0.8;1.05) | 0.26 | 0.64 (0.32;1.27) | 0.28 | **2.49 (1.82;3.42)** | **<0.001** | **1.95 (1.18;3.23)** | **0.01** |
| RH4.9 | **2.15 (1.6;2.89)** | **<0.001** | **0.65 (0.48;0.9)** | **0.01** | 0.88 (0.77;1.01) | 0.13 | **0.36 (0.19;0.7)** | **0.02** | **3.06 (2.26;4.14)** | **<0.001** | **1.93 (1.18;3.18)** | **0.01** |
| **Group iii antigens** | | | | | | | | | | | | |
| DBL-α | 0.82 (0.61;1.1) | 0.3 | **0.67 (0.5;0.9)** | **0.01** | **0.83 (0.74;0.94)** | **0.03** | 0.79 (0.42;1.49) | 0.52 | **1.5 (1.1;2.04)** | **0.01** | **1.66 (1.04;2.63)** | **0.03** |
| MSP2 FL CH150 | **1.98 (1.34;2.93)** | **0.004** | **0.4 (0.27;0.59)** | **<0.001** | 0.88 (0.74;1.04) | 0.22 | **0.23 (0.1;0.53)** | **0.005** | **1.85 (1.36;2.51)** | **<0.001** | **3.34 (1.79;6.23)** | **<0.001** |
| RH5 | 1.13 (0.81;1.56) | 0.56 | **0.54 (0.39;0.74)** | **<0.001** | 0.84 (0.73;0.97) | 0.06 | 0.56 (0.28;1.12) | 0.16 | **2.02 (1.5;2.73)** | **<0.001** | **2.58 (1.56;4.27)** | **<0.001** |
| SSP2 (TRAP) | **2.03 (1.46;2.84)** | **<0.001** | **0.62 (0.44;0.88)** | **0.01** | 0.85 (0.73;0.98) | 0.1 | **0.41 (0.2;0.86)** | **0.045** | **2.15 (1.58;2.93)** | **<0.001** | **1.83 (1.06;3.18)** | **0.03** |
| MSP1 Bl2 MAD20 | **2.03 (1.56;2.64)** | **<0.001** | **0.6 (0.46;0.79)** | **<0.001** | 0.93 (0.82;1.05) | 0.26 | **0.44 (0.24;0.79)** | **0.03** | **2.4 (1.87;3.07)** | **< 0.001** | **2.14 (1.38;3.32)** | **0.002** |
| MSP6 | 1.32 (0.89;1.96) | 0.3 | **0.49 (0.33;0.72)** | **<0.001** | 0.81 (0.68;0.95) | 0.06 | 0.53 (0.22;1.24) | 0.22 | **1.92 (1.41;2.6)** | **<0.001** | **3.9 (2.12;7.17)** | **< 0.001** |
| EBA175 R3-5 | 1.2 (0.85;1.68) | 0.41 | **0.54 (0.39;0.75)** | **< 0.001** | 0.87 (0.76;1.01) | 0.14 | 0.46 (0.22;0.95) | 0.07 | **1.39 (1.03;1.88)** | **0.03** | **2.3 (1.35;3.92)** | **0.004** |
| RH2 2030 | 1.04 (0.8;1.33) | 0.83 | **0.69 (0.54;0.89)** | **0.007** | 0.87 (0.78;0.97) | 0.06 | 0.59 (0.34;1.01) | 0.1 | **1.82 (1.35;2.45)** | **<0.001** | **1.89 (1.27;2.8)** | **0.003** |
| MSP5 | 1.47 (1.04;2.07) | 0.08 | **0.68 (0.48;0.97)** | **0.04** | 0.86 (0.74;1) | 0.13 | **0.24 (0.11;0.49)** | **0.001** | **1.63 (1.22;2.18)** | **0.001** | **1.83 (1.05;3.19)** | **0.03** |
| EBA140 R3-5 | **1.43 (1.09;1.89)** | **0.04** | 0.78 (0.59;1.04) | 0.09 | 0.9 (0.8;1.02) | 0.19 | **0.45 (0.25;0.82)** | **0.04** | **1.55 (1.17;2.05)** | **0.002** | **1.85 (1.19;2.87)** | **0.009** |
| MSP1 Bl2 RO33 | 1.26 (0.89;1.78) | 0.3 | **0.63 (0.44;0.88)** | **0.01** | 0.89 (0.77;1.04) | 0.22 | 0.67 (0.32;1.4) | 0.35 | **1.59 (1.19;2.13)** | **0.002** | **1.86 (1.08;3.22)** | **0.03** |
| MSP1 Bl2 Well | **2.87 (2.11;3.89)** | **<0.001** | 0.77 (0.54;1.08) | 0.13 | 0.95 (0.82;1.1) | 0.54 | **0.24 (0.12;0.48)** | **< 0.001** | **2.33 (1.82;2.98)** | **<0.001** | **2.1 (1.23;3.58)** | **0.009** |
| MSP1 Bl2 3D7 | 1.52 (1.07;2.16) | 0.06 | **0.58 (0.41;0.83)** | **0.005** | 0.97 (0.83;1.13) | 0.71 | **0.39 (0.18;0.82)** | **0.041** | **1.85 (1.37;2.5)** | **<0.001** | **3.75 (2.18;6.43)** | **<0.001** |
| RH4.2 | 1.32 (0.97;1.8) | 0.15 | **0.62 (0.45;0.85)** | **0.004** | 0.91 (0.8;1.05) | 0.24 | **0.25 (0.13;0.48)** | **< 0.001** | **1.65 (1.26;2.17)** | **<0.001** | **2.44 (1.5;3.96)** | **<0.001** |

HAZ: height for age Z-score. No statistical significant differences were detected for vaccination, sex, weight for age Z-score or hemoglobin (data not shown).

**b)** Antibody levels at month 0 for IgG and IgM.

| **IgG** | | | | | | |
| --- | --- | --- | --- | --- | --- | --- |
| **Antigen** | **Age (5-17 month)** | | **Site (Manhiça)** | | **Hemoglobin** | |
|  | **Coefficient** | **P** | **Coefficient** | **P** | **Coefficient** | **P** |
| **Group i antigens** | | | | | | |
| MSP1_42_ 3D7 | **0.29 (0.14;0.61)** | **0.002** | **0.05 (0.03;0.09)** | **< 0.001** | 0.94 (0.72;1.23) | 0.8 |
| MSP1_42_ FVO | 0.55 (0.24;1.22) | 0.2 | **0.05 (0.03;0.11)** | **< 0.001** | 0.86 (0.64;1.15) | 0.53 |
| EXP1 | **0.12 (0.06;0.24)** | **< 0.001** | **0.04 (0.02;0.08)** | **< 0.001** | 0.99 (0.75;1.3) | 0.93 |
| AMA1 FVO | **0.03 (0.01;0.06)** | **< 0.001** | **0.12 (0.05;0.28)** | **< 0.001** | 1.08 (0.8;1.47) | 0.79 |
| AMA1 3D7 | **0.03 (0.02;0.06)** | **< 0.001** | **0.13 (0.06;0.3)** | **< 0.001** | 1.05 (0.77;1.42) | 0.83 |
| **Group ii antigens** | | | | | | |
| pTRAMP | 1.55 (0.98;2.46) | 0.09 | **0.26 (0.17;0.4)** | **< 0.001** | 0.79 (0.67;0.93) | 0.051 |
| EBA175 R2 F2 | **0.38 (0.21;0.69)** | **0.003** | **0.18 (0.1;0.31)** | **< 0.001** | 0.99 (0.79;1.23) | 0.93 |
| RH1 | **2.06 (1.33;3.18)** | **0.003** | **0.14 (0.1;0.2)** | **< 0.001** | **0.76 (0.65;0.89)** | **0.02** |
| MSP3 3D7 | **0.16 (0.09;0.27)** | **< 0.001** | **0.2 (0.11;0.35)** | **< 0.001** | 1.16 (0.94;1.43) | 0.4 |
| VAR2CSA DBL3-4 | **0.09 (0.05;0.16)** | **< 0.001** | **0.12 (0.06;0.21)** | **< 0.001** | 1.19 (0.94;1.5) | 0.4 |
| p41 | 1.12 (0.75;1.68) | 0.6 | **0.31 (0.21;0.45)** | **< 0.001** | 0.85 (0.73;0.98) | 0.16 |
| MSP2 FL Dd2 | **0.12 (0.06;0.24)** | **< 0.001** | **0.03 (0.02;0.06)** | **< 0.001** | 0.91 (0.69;1.19) | 0.69 |
| MSP1 Bl2 hybrid | **0.44 (0.22;0.86)** | **0.03** | **0.14 (0.07;0.25)** | **< 0.001** | 0.88 (0.69;1.12) | 0.53 |
| MSP3 3C | **2.18 (1.4;3.38)** | **0.001** | **0.32 (0.21;0.49)** | **< 0.001** | 0.89 (0.76;1.05) | 0.4 |
| RH2 b240 | 0.93 (0.61;1.43) | 0.75 | **0.26 (0.17;0.38)** | **< 0.001** | 0.88 (0.76;1.03) | 0.33 |
| LSA1 | 0.56 (0.31;1.01) | 0.09 | **0.15 (0.09;0.25)** | **< 0.001** | 0.83 (0.67;1.02) | 0.33 |
| MSP1 Bl2 PA17 | 0.74 (0.43;1.29) | 0.38 | **0.3 (0.17;0.51)** | **< 0.001** | 0.9 (0.74;1.1) | 0.53 |
| VAR2CSA DBL1-2 | **0.34 (0.23;0.51)** | **< 0.001** | **0.25 (0.17;0.36)** | **< 0.001** | 1.07 (0.92;1.24) | 0.63 |
| CyRPA | **2.5 (1.93;3.26)** | **< 0.001** | **0.66 (0.5;0.89)** | **0.007** | 0.94 (0.85;1.05) | 0.53 |
| CelTOS | **2.07 (1.45;2.96)** | **< 0.001** | **0.37 (0.26;0.53)** | **< 0.001** | 0.9 (0.78;1.02) | 0.33 |
| RH4.9 | **2.45 (1.78;3.37)** | **< 0.001** | **0.64 (0.45;0.9)** | **0.01** | 0.97 (0.86;1.1) | 0.8 |
| **Group iii antigens** | | | | | | |
| DBL-α | 1.11 (0.78;1.58) | 0.6 | **0.36 (0.26;0.49)** | **< 0.001** | 0.95 (0.84;1.08) | 0.64 |
| MSP2 FL CH150 | **0.17 (0.08;0.35)** | **< 0.001** | **0.04 (0.02;0.07)** | **< 0.001** | 0.83 (0.63;1.09) | 0.4 |
| RH5 | **1.71 (1.14;2.55)** | **0.02** | **0.23 (0.16;0.32)** | **< 0.001** | 0.88 (0.77;1.02) | 0.33 |
| SSP2 (TRAP) | 0.9 (0.61;1.31) | 0.6 | **0.46 (0.32;0.66)** | **< 0.001** | 0.88 (0.77;1.01) | 0.33 |
| MSP1 Bl2 MAD20 | 0.81 (0.63;1.04) | 0.14 | **0.47 (0.37;0.59)** | **< 0.001** | 1.02 (0.93;1.11) | 0.83 |
| MSP6 | 0.8 (0.48;1.33) | 0.43 | **0.16 (0.1;0.25)** | **< 0.001** | 0.82 (0.68;0.98) | 0.17 |
| EBA175 R3-5 | **0.27 (0.15;0.48)** | **< 0.001** | **0.2 (0.11;0.36)** | **< 0.001** | 1.06 (0.85;1.31) | 0.79 |
| RH2 2030 | **0.19 (0.12;0.3)** | **< 0.001** | **0.21 (0.13;0.35)** | **< 0.001** | 1.03 (0.85;1.24) | 0.83 |
| MSP5 | 1.3 (0.77;2.19) | 0.39 | **0.24 (0.15;0.4)** | **< 0.001** | 0.88 (0.73;1.06) | 0.4 |
| EBA140 R3-5 | 1.3 (0.81;2.09) | 0.38 | **0.25 (0.16;0.39)** | **< 0.001** | 0.98 (0.82;1.16) | 0.83 |
| MSP1 Bl2 RO33 | 1.27 (0.8;2) | 0.38 | **0.25 (0.16;0.37)** | **< 0.001** | 0.8 (0.68;0.93) | 0.051 |
| MSP1 Bl2 Well | **2 (1.46;2.74)** | **< 0.001** | **0.65 (0.47;0.91)** | **0.01** | 0.96 (0.85;1.08) | 0.69 |
| MSP1 Bl2 3D7 | **0.52 (0.32;0.87)** | **0.02** | **0.26 (0.16;0.42)** | **< 0.001** | 0.93 (0.78;1.11) | 0.65 |
| RH4.2 | 1.21 (0.8;1.83) | 0.43 | **0.32 (0.22;0.48)** | **< 0.001** | 0.81 (0.7;0.94) | 0.051 |

No statistically significant differences were detected for vaccination group, sex, weight-for-age Z score, height-for-age Z score or season (data not shown).

| **IgM** | | | | | | | | |
| --- | --- | --- | --- | --- | --- | --- | --- | --- |
| **Antigen** | **Age (5-17 month)** | | **Site (Manhiça)** | | **WAZ** | | **Hemoglobin** | |
|  | **Coefficient** | **P** | **Coefficient** | **P** | **Coefficient** | **P** | **Coefficient** | **P** |
| **Group i antigens** | | | | | | | | |
| MSP1_42_ 3D7 | **1.81 (1.23;2.66)** | **0.004** | **0.41 (0.28;0.6)** | **< 0.001** | **0.72 (0.61;0.86)** | **< 0.001** | **0.77 (0.67;0.88)** | **< 0.001** |
| MSP1_42_ FVO | **2.36 (1.64;3.4)** | **< 0.001** | **0.4 (0.27;0.57)** | **< 0.001** | **0.73 (0.62;0.86)** | **< 0.001** | **0.73 (0.65;0.84)** | **< 0.001** |
| EXP1 | **3.23 (2.09;4.98)** | **< 0.001** | **0.22 (0.15;0.34)** | **< 0.001** | **0.78 (0.63;0.96)** | **0.02** | **0.73 (0.62;0.85)** | **< 0.001** |
| AMA1 FVO | 1.22 (0.91;1.65) | 0.2 | **0.53 (0.4;0.71)** | **< 0.001** | **0.76 (0.67;0.87)** | **< 0.001** | **0.88 (0.79;0.98)** | **0.02** |
| AMA1 3D7 | 1.16 (0.85;1.58) | 0.35 | **0.52 (0.38;0.7)** | **< 0.001** | **0.82 (0.71;0.94)** | **0.007** | 0.94 (0.84;1.05) | 0.29 |
| **Group ii antigens** | | | | | | | | |
| pTRAMP | **1.28 (1.02;1.61)** | **0.03** | **0.69 (0.55;0.87)** | **0.002** | **0.81 (0.73;0.89)** | **< 0.001** | **0.89 (0.82;0.96)** | **0.003** |
| EBA175 R2 F2 | **1.75 (1.27;2.4)** | **0.001** | **0.49 (0.36;0.67)** | **< 0.001** | **0.82 (0.71;0.94)** | **0.009** | **0.79 (0.7;0.88)** | **< 0.001** |
| RH1 | **1.84 (1.41;2.4)** | **< 0.001** | **0.66 (0.5;0.87)** | **0.004** | **0.82 (0.72;0.92)** | **0.002** | **0.84 (0.76;0.93)** | **< 0.001** |
| MSP3 3D7 | 1.24 (0.88;1.76) | 0.23 | **0.61 (0.43;0.87)** | **0.008** | **0.77 (0.66;0.89)** | **0.002** | **0.77 (0.68;0.87)** | **< 0.001** |
| VAR2CSA DBL3-4 | **1.65 (1.28;2.11)** | **< 0.001** | **0.61 (0.48;0.79)** | **< 0.001** | **0.81 (0.72;0.9)** | **< 0.001** | **0.87 (0.79;0.95)** | **0.003** |
| p41 | **1.54 (1.17;2.02)** | **0.003** | **0.57 (0.44;0.75)** | **< 0.001** | **0.82 (0.73;0.93)** | **0.003** | **0.8 (0.73;0.88)** | **< 0.001** |
| MSP2 FL Dd2 | **3.52 (2.29;5.43)** | **< 0.001** | **0.32 (0.2;0.5)** | **< 0.001** | **0.76 (0.62;0.93)** | **0.01** | **0.7 (0.59;0.82)** | **< 0.001** |
| MSP1 Bl2 hybrid | **2.48 (1.62;3.81)** | **< 0.001** | **0.57 (0.37;0.89)** | **0.02** | **0.74 (0.61;0.9)** | **0.004** | **0.8 (0.68;0.93)** | **0.006** |
| MSP3 3C | **2.09 (1.36;3.19)** | **0.001** | 0.69 (0.45;1.08) | 0.11 | **0.83 (0.69;1.02)** | **0.07** | **0.81 (0.69;0.94)** | **0.006** |
| RH2 b240 | **2.21 (1.61;3.01)** | **< 0.001** | **0.57 (0.41;0.79)** | **0.002** | **0.77 (0.66;0.88)** | **< 0.001** | **0.8 (0.71;0.9)** | **< 0.001** |
| LSA1 | **4.31 (2.72;6.81)** | **< 0.001** | **0.21 (0.13;0.32)** | **< 0.001** | **0.81 (0.65;1.02)** | **0.07** | **0.63 (0.53;0.75)** | **< 0.001** |
| MSP1 Bl2 PA17 | **2.17 (1.43;3.28)** | **< 0.001** | **0.48 (0.31;0.73)** | **0.001** | **0.7 (0.58;0.84)** | **< 0.001** | **0.81 (0.7;0.94)** | **0.006** |
| Var2csa DBL1-2 | **2.31 (1.68;3.18)** | **< 0.001** | **0.56 (0.4;0.78)** | **0.001** | **0.76 (0.66;0.88)** | **< 0.001** | **0.79 (0.7;0.88)** | **< 0.001** |
| CYRPA | **1.47 (1.05;2.07)** | **0.03** | **0.42 (0.3;0.59)** | **< 0.001** | **0.83 (0.71;0.97)** | **0.02** | **0.77 (0.68;0.87)** | **< 0.001** |
| CelTOS | **2.16 (1.63;2.87)** | **< 0.001** | 0.81 (0.59;1.1) | 0.17 | **0.81 (0.7;0.92)** | **0.003** | **0.82 (0.74;0.91)** | **< 0.001** |
| RH4.9 | **3.06 (2.37;3.96)** | **< 0.001** | **0.67 (0.49;0.9)** | **0.01** | **0.79 (0.69;0.9)** | **0.001** | **0.85 (0.76;0.94)** | **0.003** |
| **Group iii antigens** | | | | | | | | |
| DBL-α | **1.46 (1.08;1.97)** | **0.02** | **0.6 (0.45;0.81)** | **0.002** | **0.81 (0.71;0.92)** | **0.003** | **0.82 (0.74;0.91)** | **< 0.001** |
| MSP2 FL CH150 | **3.14 (2.15;4.59)** | **< 0.001** | **0.32 (0.22;0.47)** | **< 0.001** | **0.73 (0.61;0.87)** | **0.001** | **0.78 (0.67;0.9)** | **0.001** |
| RH5 | **1.79 (1.29;2.49)** | **0.001** | **0.43 (0.31;0.59)** | **< 0.001** | **0.83 (0.72;0.97)** | **0.02** | **0.82 (0.73;0.92)** | **0.001** |
| SSP2 (TRAP) | **2.63 (1.91;3.62)** | **< 0.001** | **0.68 (0.48;0.96)** | **0.03** | **0.85 (0.73;0.99)** | **0.04** | **0.8 (0.71;0.9)** | **< 0.001** |
| MSP1 Bl2 MAD20 | **2.56 (1.89;3.48)** | **< 0.001** | **0.51 (0.37;0.71)** | **< 0.001** | **0.73 (0.63;0.84)** | **< 0.001** | **0.82 (0.73;0.92)** | **0.001** |
| MSP6 | **2.49 (1.69;3.67)** | **< 0.001** | **0.43 (0.29;0.64)** | **< 0.001** | **0.72 (0.6;0.86)** | **< 0.001** | **0.76 (0.66;0.87)** | **< 0.001** |
| EBA175 R3-5 | **1.82 (1.27;2.6)** | **0.002** | **0.52 (0.36;0.74)** | **< 0.001** | **0.78 (0.66;0.91)** | **0.004** | **0.81 (0.71;0.92)** | **0.002** |
| RH2 2030 | **1.39 (1.07;1.82)** | **0.02** | 0.77 (0.59;1.01) | 0.06 | **0.87 (0.77;0.98)** | **0.02** | **0.89 (0.81;0.98)** | **0.02** |
| MSP5 | **2.19 (1.52;3.17)** | **< 0.001** | 0.69 (0.47;1.02) | 0.07 | **0.79 (0.67;0.94)** | **0.01** | **0.76 (0.67;0.87)** | **< 0.001** |
| EBA140 R3-5 | **1.72 (1.26;2.35)** | **0.001** | **0.62 (0.45;0.85)** | **0.004** | **0.75 (0.65;0.86)** | **< 0.001** | **0.77 (0.69;0.86)** | **< 0.001** |
| MSP1 Bl2 RO33 | **1.75 (1.21;2.54)** | **0.004** | **0.43 (0.3;0.62)** | **< 0.001** | **0.84 (0.71;0.99)** | **0.04** | **0.77 (0.67;0.87)** | **< 0.001** |
| MSP1 Bl2 Well | **3.02 (2.08;4.39)** | **< 0.001** | **0.62 (0.42;0.93)** | **0.03** | **0.71 (0.59;0.84)** | **< 0.001** | **0.82 (0.71;0.94)** | **0.006** |
| MSP1 Bl2 3D7 | **2.18 (1.52;3.11)** | **< 0.001** | **0.6 (0.41;0.86)** | **0.008** | **0.74 (0.63;0.87)** | **< 0.001** | **0.81 (0.71;0.93)** | **0.003** |
| RH4.2 | **2.71 (1.94;3.79)** | **< 0.001** | **0.55 (0.38;0.79)** | **0.002** | **0.74 (0.63;0.87)** | **< 0.001** | **0.75 (0.67;0.85)** | **< 0.001** |

WAZ: weight-for-age Z score. No statistically significant differences were detected for vaccination group, sex, height-for-age Z score or season (data not shown).

**Table S4. Multivariable analysis of the effect of RTS,S/AS01E vaccination and demographic, clinical and epidemiological variables on antibody levels to *Plasmodium falciparum* pre-erythrocytic and blood stage antigens.** Linear regression with antibody levels (log_10_MFI) as outcome and other covariates adjusted (in parenthesis the category that is compared to the reference). Coefficients (10^Beta) with 95% confidence intervals and p values are shown (significant in bold).

**a)** Month 3 IgM

| **Antigen** | **Vaccine (RTS,S)** | | **Age (5-17 month)** | | **Site (Manhiça)** | | **Baseline IgM levels** | | **Malaria episodes** | | **Season (Low)** | | **HAZ** | | **Sex (Male)** | |
| --- | --- | --- | --- | --- | --- | --- | --- | --- | --- | --- | --- | --- | --- | --- | --- | --- |
|  | **Coefficient** | **P** | **Coefficient** | **P** | **Coefficient** | **P** | **Coefficient** | **P** | **Coefficient** | **P** | **Coefficient** | **P** | **Coefficient** | **P** | **Coefficient** | **P** |
| **Group i antigens** | | | | | | | | | | | | | | | | |
| MSP1_42_ 3D7 | **0.49 (0.33;0.73)** | **<0.001** | 0.73 (0.51;1.05) | 0.09 | **0.57 (0.38;0.84)** | **0.005** |  |  | **3.23 (1.75;5.95)** | **< 0.001** | 0.47 (0.21;1.07) | 0.07 |  |  | 1.39 (0.97;1.98) | 0.07 |
| MSP1_42_ FVO | 0.75 (0.5;1.12) | 0.15 |  |  | 0.7 (0.45;1.07) | 0.1 | **1.49 (1.06;2.09)** | **0.02** | **2.75 (1.44;5.23)** | **0.002** | 0.44 (0.19;1.02) | 0.055 | 0.87 (0.74;1.03) | 0.1 |  |  |
| EXP1 |  |  | **1.93 (1.17;3.16)** | **0.01** |  |  | **1.45 (1.01;2.08)** | **0.042** | **6.66 (3.1;14.31)** | **<0.001** | 0.48 (0.18;1.3) | 0.15 |  |  |  |  |
| AMA1 FVO |  |  | 0.79 (0.6;1.04) | 0.09 | 0.75 (0.55;1.01) | 0.06 | **1.49 (1.08;2.04)** | **0.01** | **1.68 (1.05;2.71)** | **0.03** |  |  |  |  | **1.34 (1.02;1.76)** | **0.04** |
| AMA1 3D7 |  |  | 0.81 (0.61;1.09) | 0.16 | **0.68 (0.49;0.94)** | **0.02** | 1.36 (0.99;1.87) | 0.057 | **1.89 (1.15;3.11)** | **0.01** |  |  |  |  |  |  |
| **Group ii antigens** | | | | | | | | | | | | | | | | |
| pTRAMP |  |  | 0.8 (0.64;1.01) | 0.06 | 0.79 (0.62;1.02) | 0.07 | **1.73 (1.22;2.44)** | **0.002** | 1.42 (0.96;2.12) | 0.08 |  |  | **0.88 (0.79;0.97)** | **0.01** | 1.21 (0.97;1.52) | 0.09 |
| EBA175 R2(F2) |  |  |  |  | **0.63 (0.44;0.89)** | **0.009** | **1.66 (1.19;2.31)** | **0.003** | 1.53 (0.89;2.61) | 0.12 |  |  |  |  |  |  |
| PfRH1 |  |  |  |  | **0.77 (0.6;1)** | **0.05** | **2.07 (1.55;2.76)** | **<0.001** | 1.4 (0.93;2.11) | 0.11 |  |  |  |  |  |  |
| MSP3 3D7 |  |  |  |  |  |  | **1.87 (1.41;2.47)** | **<0.001** |  |  | 0.57 (0.3;1.08) | 0.09 |  |  |  |  |
| VAR2CSA DBL3-4 |  |  | 0.84 (0.65;1.07) | 0.15 | **0.73 (0.57;0.93)** | **0.01** | **1.82 (1.32;2.51)** | **<0.001** |  |  |  |  | 0.9 (0.81;1) | 0.054 |  |  |
| p41 |  |  |  |  | 0.78 (0.59;1.05) | 0.1 | **1.77 (1.28;2.45)** | **<0.001** | 1.53 (0.97;2.41) | 0.07 |  |  |  |  | **1.3 (1;1.69)** | **0.048** |
| MSP2 FL Dd2 |  |  | 1.4 (0.94;2.07) | 0.1 |  |  | **1.58 (1.19;2.09)** | **0.002** | **2.48 (1.38;4.47)** | **0.003** | **0.27 (0.12;0.6)** | **0.001** | 0.86 (0.74;1) | 0.057 |  |  |
| MSP1 Bl2 hybrid |  |  | 1.34 (0.9;1.99) | 0.15 |  |  | **1.43 (1.07;1.93)** | **0.02** | **3.15 (1.7;5.86)** | **< 0.001** | **0.44 (0.19;0.99)** | **0.047** |  |  |  |  |
| MSP3 3C |  |  | **0.64 (0.44;0.94)** | **0.02** |  |  | **2.2 (1.65;2.93)** | **<0.001** | **2.02 (1.11;3.67)** | **0.02** |  |  |  |  | 0.71 (0.49;1.03) | 0.07 |
| PfRH2 b240 |  |  |  |  |  |  | **1.96 (1.46;2.62)** | **<0.001** | **1.85 (1.15;2.97)** | **0.01** | **0.49 (0.27;0.92)** | **0.03** | **0.87 (0.77;0.99)** | **0.03** | 1.29 (0.97;1.72) | 0.08 |
| LSA1 | 0.73 (0.48;1.11) | 0.14 |  |  | **0.38 (0.24;0.61)** | **< 0.001** | **1.43 (1.07;1.92)** | **0.02** | 1.95 (0.97;3.9) | 0.06 |  |  |  |  |  |  |
| MSP1 Bl2 PA17 |  |  |  |  |  |  | **1.37 (0.98;1.92)** | **0.07** | **4.66 (2.34;9.27)** | **< 0.001** | 0.48 (0.2;1.19) | 0.11 |  |  |  |  |
| VAR2CSA DBL1-2 |  |  | 0.76 (0.54;1.08) | 0.12 | **0.59 (0.42;0.83)** | **0.002** | **1.74 (1.24;2.43)** | **0.001** |  |  | 0.53 (0.26;1.07) | 0.08 |  |  |  |  |
| CYRPA |  |  | **0.68 (0.5;0.93)** | **0.02** | **0.52 (0.37;0.73)** | **< 0.001** | **2.14 (1.57;2.92)** | **<0.001** |  |  |  |  | **0.87 (0.76;0.99)** | **0.042** | 1.31 (0.97;1.78) | 0.08 |
| CelTOS |  |  |  |  | **0.67 (0.5;0.9)** | **0.009** | **2.37 (1.73;3.26)** | **< 0.001** |  |  |  |  |  |  |  |  |
| RH4.9 |  |  | 1.35 (0.97;1.88) | 0.07 |  |  | **2.38 (1.67;3.39)** | **< 0.001** | 1.51 (0.97;2.36) | 0.07 | 0.57 (0.31;1.04) | 0.07 | 0.91 (0.81;1.03) | 0.13 | **1.38 (1.05;1.81)** | **0.02** |
| **Group iii antigens** | | | | | | | | | | | | | | | | |
| DBL-α |  |  | 0.77 (0.58;1.02) | 0.07 | 0.79 (0.58;1.09) | 0.15 | **1.37 (1;1.89)** | **0.05** | 1.43 (0.88;2.35) | 0.15 |  |  | **0.87 (0.77;0.99)** | **0.04** | 1.24 (0.93;1.64) | 0.14 |
| MSP2 FL CH150 |  |  | **1.64 (1.12;2.39)** | **0.01** | **0.51 (0.34;0.77)** | **0.001** |  |  | **2.04 (1.09;3.82)** | **0.03** | **0.41 (0.18;0.93)** | **0.03** |  |  |  |  |
| PfRH5 |  |  |  |  | 0.75 (0.53;1.07) | 0.11 | **1.64 (1.2;2.24)** | **0.002** | **1.79 (1.05;3.04)** | **0.03** |  |  | 0.88 (0.77;1) | 0.054 |  |  |
| SSP2 (TRAP) | **1.41 (1.01;1.98)** | **0.044** | **1.63 (1.15;2.31)** | **0.006** | 0.72 (0.52;1.01) | 0.058 | **1.63 (1.17;2.27)** | **0.004** |  |  |  |  | 0.89 (0.77;1.02) | 0.1 |  |  |
| MSP1 Bl2 MAD20 |  |  | **1.47 (1.12;1.92)** | **0.006** |  |  | **1.92 (1.47;2.51)** | **<0.001** | **1.55 (1.04;2.32)** | **0.03** | 0.64 (0.37;1.09) | 0.1 |  |  |  |  |
| MSP6 |  |  |  |  |  |  | **1.57 (1.15;2.13)** | **0.005** | **3.26 (1.77;6)** | **< 0.001** |  |  | **0.81 (0.69;0.95)** | **0.01** |  |  |
| RH2 (2030) |  |  |  |  |  |  | **1.65 (1.22;2.22)** | **0.001** | **1.67 (1.13;2.47)** | **0.01** | 0.63 (0.37;1.05) | 0.08 | **0.89 (0.8;0.98)** | **0.02** |  |  |
| EBA175 R3-5 |  |  |  |  | **0.62 (0.43;0.89)** | **0.01** |  |  | **1.8 (1.03;3.15)** | **0.04** |  |  |  |  | 1.26 (0.91;1.74) | 0.16 |
| MSP5 |  |  |  |  |  |  | **1.45 (1.09;1.93)** | **0.01** | 1.51 (0.88;2.59) | 0.13 | **0.28 (0.14;0.58)** | **< 0.001** | 0.9 (0.78;1.04) | 0.14 |  |  |
| EBA140 R3-5 |  |  | 1.25 (0.94;1.66) | 0.13 |  |  | 1.28 (0.95;1.73) | 0.11 | **1.59 (1.02;2.5)** | **0.042** | 0.56 (0.31;1.02) | 0.058 | 0.92 (0.81;1.03) | 0.15 |  |  |
| MSP1 Bl2 RO33 |  |  |  |  | 0.71 (0.5;1.03) | 0.07 | **1.45 (1.07;1.98)** | **0.02** |  |  |  |  |  |  |  |  |
| MSP1 Bl2 Well |  |  | **1.94 (1.42;2.65)** | **< 0.001** |  |  | **1.74 (1.36;2.24)** | **<0.001** | 1.5 (0.95;2.39) | 0.08 | **0.39 (0.21;0.72)** | **0.003** |  |  |  |  |
| MSP1 Bl2 3D7 | 0.72 (0.5;1.04) | 0.08 |  |  |  |  | **1.53 (1.14;2.06)** | **0.005** | **2.79 (1.62;4.79)** | **< 0.001** | **0.39 (0.19;0.81)** | **0.01** |  |  |  |  |
| RH4.2 |  |  |  |  |  |  | **1.44 (1.1;1.88)** | **0.008** | **1.89 (1.18;3.03)** | **0.009** | **0.29 (0.16;0.54)** | **< 0.001** |  |  |  |  |

HAZ: height-for-age Z score. Hemoglobin and weight-for-age Z score were retained in some antigen models but were not statistically significant (data not shown).

**b)** Month 0

| **IgM** | | | | | | | | | | | | | | |
| --- | --- | --- | --- | --- | --- | --- | --- | --- | --- | --- | --- | --- | --- | --- |
| **Antigen** | **Age (5-17 month)** | | **Site (Manhiça)** | | **Season (Low)** | | **Hemoglobin** | | **WAZ** | | **HAZ** | | **Sex (Male)** | |
|  | **Coefficient** | **P** | **Coefficient** | **P** | **Coefficient** | **P** | **Coefficient** | **P** | **Coefficient** | **P** | **Coefficient** | **P** | **Coefficient** | **P** |
| **Group i antigens** | | | | | | | | | | | | | | |
| MSP1_42_ 3D7 | 1.44 (0.99;2.1) | 0.058 | **0.52 (0.35;0.78)** | **0.001** | **3.32 (1.53;7.19)** | **0.003** | **0.82 (0.72;0.94)** | **0.004** | **0.72 (0.58;0.89)** | **0.002** | 1.17 (0.96;1.43) | 0.12 |  |  |
| MSP1_42_ FVO | **1.8 (1.28;2.53)** | **<0.001** | **0.59 (0.41;0.85)** | **0.005** |  |  | **0.81 (0.71;0.91)** | **<0.001** | **0.83 (0.71;0.97)** | **0.02** |  |  |  |  |
| EXP1 | **2.56 (1.73;3.79)** | **<0.001** | **0.34 (0.22;0.53)** | **<0.001** |  |  | **0.84 (0.73;0.96)** | **0.01** |  |  | 0.87 (0.73;1.03) | 0.1 |  |  |
| AMA1 FVO |  |  | **0.71 (0.52;0.97)** | **0.03** |  |  | 0.92 (0.83;1.02) | 0.1 | **0.8 (0.71;0.91)** | **0.001** |  |  |  |  |
| AMA1 3D7 |  |  | **0.65 (0.46;0.9)** | **0.01** |  |  |  |  |  |  | **0.86 (0.75;0.98)** | **0.02** |  |  |
| **Group ii antigens** | | | | | | | | | | | | | | |
| pTRAMP |  |  |  |  | 1.4 (0.9;2.17) | 0.13 | **0.89 (0.83;0.96)** | **0.003** | **0.82 (0.75;0.9)** | **<0.001** |  |  | 0.86 (0.7;1.05) | 0.14 |
| EBA175 R2(F2) | **1.54 (1.13;2.1)** | **0.007** | **0.65 (0.47;0.91)** | **0.01** | 1.62 (0.84;3.12) | 0.15 | **0.84 (0.75;0.93)** | **0.001** |  |  | 0.89 (0.78;1.02) | 0.09 |  |  |
| PfRH1 | **1.56 (1.2;2.02)** | **<0.001** |  |  |  |  | **0.89 (0.81;0.98)** | **0.01** | **0.86 (0.76;0.96)** | **0.008** |  |  |  |  |
| MSP3 3D7 |  |  | **0.7 (0.5;0.99)** | **0.043** |  |  | **0.8 (0.71;0.91)** | **<0.001** | **0.84 (0.72;0.97)** | **0.02** |  |  |  |  |
| VAR2CSA DBL3-4 | **1.44 (1.13;1.83)** | **0.004** | 0.81 (0.62;1.05) | 0.12 |  |  | 0.92 (0.84;1.01) | 0.07 | **0.85 (0.76;0.95)** | **0.004** |  |  |  |  |
| p41 | 1.24 (0.96;1.61) | 0.1 | 0.77 (0.58;1.01) | 0.06 |  |  | **0.83 (0.76;0.91)** | **<0.001** | **0.88 (0.78;0.99)** | **0.03** |  |  |  |  |
| MSP2 FL Dd2 | **2.65 (1.76;4)** | **0.001** | **0.44 (0.29;0.69)** | **< 0.001** |  |  | **0.79 (0.68;0.91)** | **0.002** |  |  |  |  |  |  |
| MSP1 Bl2 hybrid | **2.23 (1.46;3.39)** | **<0.001** |  |  |  |  | 0.87 (0.75;1.02) | 0.08 |  |  | **0.78 (0.65;0.93)** | **0.006** |  |  |
| MSP3 3C | **1.8 (1.16;2.79)** | **0.009** |  |  |  |  | **0.85 (0.72;0.99)** | **0.04** |  |  |  |  |  |  |
| PfRH2 b240 | **1.94 (1.42;2.65)** | **<0.001** | 0.77 (0.55;1.08) | 0.13 | 1.63 (0.84;3.13) | 0.14 | **0.87 (0.78;0.97)** | **0.01** | **0.84 (0.73;0.96)** | **0.01** |  |  |  |  |
| LSA1 | **3 (2;4.5)** | **<0.001** | **0.29 (0.19;0.44)** | **< 0.001** |  |  | **0.74 (0.64;0.85)** | **<0.001** |  |  |  |  |  |  |
| MSP1 Bl2 PA17 | **1.74 (1.16;2.61)** | **0.008** |  |  |  |  | 0.88 (0.76;1.01) | 0.08 | **0.74 (0.61;0.88)** | **<0.001** |  |  |  |  |
| VAR2CSA DBL1-2 | **1.92 (1.41;2.62)** | **<0.001** |  |  |  |  | **0.85 (0.76;0.95)** | **0.004** | **0.83 (0.72;0.95)** | **0.007** |  |  |  |  |
| CyRPA |  |  | **0.55 (0.39;0.78)** | **< 0.001** |  |  | **0.78 (0.7;0.88)** | **<0.001** |  |  |  |  |  |  |
| CelTOS | **1.98 (1.48;2.64)** | **<0.001** |  |  | **1.9 (1.04;3.48)** | **0.04** | **0.88 (0.8;0.98)** | **0.02** | **0.86 (0.76;0.98)** | **0.02** |  |  |  |  |
| RH4.9 | **2.86 (2.22;3.68)** | **<0.001** |  |  |  |  |  |  | **0.83 (0.74;0.93)** | **0.002** |  |  |  |  |
| **Group iii antigens** | | | | | | | | | | | | | | |
| DBL-α |  |  |  |  |  |  | **0.83 (0.75;0.92)** | **<0.001** | **0.84 (0.73;0.96)** | **0.008** |  |  |  |  |
| MSP2 FL CH150 | **2.94 (2.04;4.24)** | **<0.001** | **0.45 (0.3;0.66)** | **<0.001** | 2 (0.9;4.43) | 0.09 | 0.9 (0.79;1.02) | 0.1 |  |  | **0.83 (0.71;0.97)** | **0.02** |  |  |
| PfRH5 | **1.68 (1.21;2.32)** | **0.002** | **0.56 (0.4;0.79)** | **0.001** | **2.04 (1.03;4.01)** | **0.04** | **0.87 (0.78;0.98)** | **0.02** |  |  | 0.9 (0.78;1.03) | 0.12 |  |  |
| SSP2 (TRAP) | **2.36 (1.71;3.25)** | **<0.001** |  |  |  |  | **0.88 (0.78;0.99)** | **0.03** |  |  | **0.87 (0.76;0.99)** | **0.04** |  |  |
| MSP1 Bl2 Mad20 | **2.11 (1.57;2.83)** | **<0.001** | **0.7 (0.51;0.96)** | **0.03** |  |  | 0.92 (0.83;1.02) | 0.12 | **0.8 (0.7;0.91)** | **<0.001** |  |  |  |  |
| MSP6 | **1.92 (1.32;2.79)** | **<0.001** | **0.64 (0.43;0.97)** | **0.03** |  |  | **0.84 (0.73;0.96)** | **0.01** | **0.81 (0.68;0.96)** | **0.02** |  |  |  |  |
| RH2 (2030) | **1.32 (1;1.73)** | **0.048** |  |  | **1.93 (1.1;3.41)** | **0.02** | 0.92 (0.84;1.01) | 0.09 | **0.88 (0.78;0.99)** | **0.04** |  |  |  |  |
| EBA175 R3-5 | **1.59 (1.11;2.27)** | **0.01** | 0.71 (0.48;1.04) | 0.08 | 1.81 (0.85;3.84) | 0.12 | **0.86 (0.76;0.98)** | **0.02** | **0.84 (0.72;0.99)** | **0.04** |  |  |  |  |
| MSP5 | **1.76 (1.21;2.55)** | **0.003** |  |  |  |  | **0.82 (0.72;0.93)** | **0.003** | 0.86 (0.73;1.01) | 0.07 |  |  |  |  |
| EBA140 R3-5 | 1.32 (0.99;1.77) | 0.06 |  |  |  |  | **0.81 (0.73;0.9)** | **<0.001** | **0.8 (0.7;0.91)** | **<0.001** |  |  |  |  |
| MSP1 Bl2 RO33 | 1.36 (0.96;1.93) | 0.08 | **0.6 (0.41;0.87)** | **0.008** |  |  | **0.8 (0.7;0.91)** | **<0.001** |  |  |  |  |  |  |
| MSP1 Bl2 Well | **2.65 (1.84;3.82)** | **<0.001** |  |  |  |  |  |  | **0.74 (0.63;0.87)** | **<0.001** |  |  |  |  |
| MSP1 Bl2 3D7 | **1.77 (1.25;2.52)** | **0.002** |  |  |  |  | **0.88 (0.78;1)** | **0.051** | **0.79 (0.67;0.92)** | **0.003** |  |  |  |  |
| RH4.2 | **2.13 (1.54;2.94)** | **<0.001** |  |  |  |  | **0.83 (0.74;0.93)** | **0.002** | **0.81 (0.7;0.94)** | **0.004** |  |  |  |  |

| **IgG** | | | | | | | | | | | | | | |
| --- | --- | --- | --- | --- | --- | --- | --- | --- | --- | --- | --- | --- | --- | --- |
| **Antigen** | **Age (5-17 month)** | | **Site (Manhiça)** | | **Season (Low)** | | **Hemoglobin** | | **WAZ** | | **HAZ** | | **Sex (Male)** | |
|  | **Coefficient** | **P** | **Coefficient** | **P** | **Coefficient** | **P** | **Coefficient** | **P** | **Coefficient** | **P** | **Coefficient** | **P** | **Coefficient** | **P** |
| **Group i antigens** | | | | | | | | | | | | | | |
| MSP1_42_ 3D7 | **0.21 (0.12;0.38)** | **<0.001** | **0.05 (0.03;0.09)** | **<0.001** |  |  |  |  |  |  |  |  |  |  |
| MSP1_42_ FVO | **0.4 (0.2;0.8)** | **0.01** | **0.04 (0.02;0.09)** | **<0.001** |  |  |  |  |  |  |  |  |  |  |
| EXP1 | **0.09 (0.05;0.15)** | **<0.001** | **0.04 (0.02;0.07)** | **<0.001** |  |  |  |  |  |  |  |  |  |  |
| AMA1 FVO | **0.02 (0.01;0.04)** | **<0.001** | **0.1 (0.05;0.18)** | **<0.001** |  |  | 0.84 (0.67;1.04) | 0.11 |  |  |  |  |  |  |
| AMA1 3D7 | **0.02 (0.01;0.04)** | **<0.001** | **0.1 (0.05;0.2)** | **<0.001** |  |  | 0.81 (0.64;1.02) | 0.07 |  |  |  |  |  |  |
| **Group ii antigens** | | | | | | | | | | | | | | |
| pTRAMP |  |  | **0.35 (0.22;0.55)** | **<0.001** |  |  | **0.81 (0.7;0.94)** | **0.005** |  |  |  |  |  |  |
| EBA175 R2(F2) | **0.32 (0.18;0.55)** | **<0.001** | **0.16 (0.09;0.27)** | **<0.001** |  |  |  |  |  |  |  |  |  |  |
| PfRH1 | **1.56 (1.09;2.22)** | **0.01** | **0.18 (0.12;0.26)** | **<0.001** |  |  | **0.84 (0.74;0.96)** | **0.009** |  |  |  |  |  |  |
| MSP3 3D7 | **0.14 (0.09;0.22)** | **<0.001** | **0.17 (0.11;0.28)** | **<0.001** |  |  |  |  |  |  |  |  |  |  |
| VAR2CSA DBL3-4 | **0.07 (0.04;0.11)** | **<0.001** | **0.11 (0.07;0.17)** | **<0.001** | **0.25 (0.09;0.7**) | **0.008** |  |  |  |  |  |  |  |  |
| p41 |  |  | **0.38 (0.25;0.57)** | **<0.001** |  |  | **0.88 (0.77;1)** | **0.054** |  |  |  |  |  |  |
| MSP2 FL Dd2 | **0.08 (0.05;0.12)** | **<0.001** | **0.03 (0.02;0.06)** | **<0.001** |  |  | **0.82 (0.68;0.98)** | **0.03** |  |  |  |  |  |  |
| MSP1 Bl2 hybrid | **0.35 (0.19;0.63)** | **<0.001** | **0.11 (0.06;0.21)** | **<0.001** |  |  |  |  |  |  |  |  |  |  |
| MSP3 3C | **2.01 (1.32;3.05)** | **0.001** | **0.33 (0.22;0.51)** | **<0.001** |  |  |  |  |  |  |  |  |  |  |
| PfRH2 b240 |  |  | **0.28 (0.18;0.42)** | **<0.001** |  |  |  |  |  |  |  |  |  |  |
| LSA1 | **0.47 (0.27;0.79)** | **0.005** | **0.14 (0.08;0.24)** | **<0.001** |  |  | **0.8 (0.66;0.97)** | **0.02** | 1.32 (1.04;1.68) | 0.02 |  |  |  |  |
| MSP1 Bl2 PA17 | 0.65 (0.38;1.1) | 0.11 | **0.27 (0.16;0.46)** | **<0.001** |  |  |  |  |  |  |  |  |  |  |
| VAR2CSA DBL1-2 | **0.29 (0.21;0.41)** | **<0.001** | **0.24 (0.17;0.33)** | **<0.001** | 0.52 (0.25;1.1) | 0.09 |  |  |  |  |  |  | 1.27 (0.91;1.77) | 0.15 |
| CyRPA | **2.41 (1.86;3.14)** | **<0.001** | **0.7 (0.54;0.92)** | **0.009** |  |  |  |  |  |  |  |  |  |  |
| CelTOS | **1.87 (1.34;2.61)** | **<0.001** | **0.38 (0.27;0.53)** | **<0.001** |  |  |  |  |  |  |  |  |  |  |
| RH4.9 | **2.34 (1.71;3.2)** | **<0.001** | **0.64 (0.46;0.88)** | **0.007** |  |  |  |  |  |  | 1.17 (1.02;1.34) | **0.02** |  |  |
| **Group iii antigens** | | | | | | | | | | | | | | |
| DBL-α |  |  | **0.42 (0.29;0.6)** | **<0.001** |  |  |  |  |  |  | 0.9 (0.78;1.03) | 0.12 |  |  |
| MSP2 FL CH150 | **0.11 (0.06;0.19)** | **<0.001** | **0.04 (0.02;0.06)** | **<0.001** |  |  | **0.78 (0.64;0.95)** | **0.01** |  |  |  |  |  |  |
| PfRH5 | **1.5 (1.06;2.13)** | **0.02** | **0.25 (0.17;0.37)** | **<0.001** |  |  |  |  |  |  |  |  |  |  |
| SSP2 (TRAP) |  |  | **0.47 (0.32;0.69)** | **<0.001** |  |  |  |  |  |  | 0.88 (0.75;1.03) | 0.1 |  |  |
| MSP1 Bl2 Mad20 | **0.75 (0.6;0.94)** | **0.01** | **0.46 (0.37;0.59)** | **<0.001** |  |  |  |  |  |  | 0.92 (0.83;1.02) | 0.11 | 0.82 (0.65;1.03) | 0.08 |
| MSP6 | **0.63 (0.4;0.99)** | **0.045** | **0.19 (0.12;0.31)** | **<0.001** |  |  | **0.84 (0.71;0.99)** | **0.03** |  |  |  |  | 1.47 (0.95;2.27) | 0.09 |
| RH2 (2030) | **0.17 (0.11;0.26)** | **<0.001** | **0.24 (0.15;0.38)** | **<0.001** |  |  |  |  |  |  | **0.83 (0.69;1)** | **0.045** |  |  |
| EBA175 R3-5 | **0.24 (0.14;0.4)** | **<0.001** | **0.16 (0.09;0.27)** | **<0.001** |  |  |  |  | 1.23 (0.96;1.56) | 0.1 |  |  |  |  |
| MSP5 |  |  | **0.27 (0.16;0.45)** | **< 0.001** |  |  |  |  |  |  | 0.84 (0.68;1.04) | 0.11 |  |  |
| EBA140 R3-5 |  |  | **0.23 (0.15;0.37)** | **< 0.001** |  |  |  |  |  |  |  |  |  |  |
| MSP1 Bl2 RO33 |  |  | **0.3 (0.19;0.47)** | **< 0.001** |  |  | **0.82 (0.71;0.95)** | **0.009** |  |  |  |  | 0.74 (0.49;1.11) | 0.15 |
| MSP1 Bl2 Well | **1.92 (1.4;2.63)** | **< 0.001** | **0.68 (0.49;0.93)** | **0.02** |  |  |  |  |  |  |  |  |  |  |
| MSP1 Bl2 3D7 | **0.45 (0.28;0.71)** | **< 0.001** | **0.27 (0.16;0.44)** | **< 0.001** |  |  |  |  |  |  |  |  |  |  |
| RH4.2 |  |  | **0.39 (0.26;0.6)** | **< 0.001** |  |  | **0.84 (0.73;0.96)** | **0.01** |  |  |  |  |  |  |

HAZ: height-for-age Z score; WAZ: weight-for-age Z score.

**SUPPLEMENTARY MATERIALS AND METHODS**

**Antibody measurements.** Antigen-coupled beads were added to a 96-well μClear® flat bottom plate (Greiner Bio-One) in multiplex (1,000 microspheres/analyte/well) resuspended in 50µL of PBS, 1% BSA, 0.05% Azide pH 7.4 (PBS-BN). Fifty µL of sample, negative or positive control were added to multiplex wells and incubated overnight at 4ºC in a shaker protected from light. Plates were washed three times with 200µL/well of wash buffer (PBS-Tween 20 0.05%) using a manual magnetic washer. Then, 100µL of biotinylated secondary antibody were added diluted in PBS-BN: anti-human IgG (Sigma) and anti-human IgM (Sigma) were added. All antibody incubations were performed for 45 min, at room temperature, with agitation and protected from light. Next, streptavidin-R-phycoerythrin (Sigma) in PBS-BN was added to all wells and incubated 30 min, at room temperature, with agitation and protected from light. Plates were washed as before and resuspended in 100 μL/well of PBS-BN. Plates were stored at 4°C overnight protected from light and read the next day using the Luminex xMAP® 100/200 analyser; at least 50 microspheres per analyte were acquired per sample.

Test samples were assayed at 4 dilutions for IgG (500, 5000, 50,000 and 500,000) and IgM (100, 1000, 10,000 and 50,000) to ensure that at least one dilution lie in the linear range of the respective standard curve, i.e. close to the highest slope between two dilution points. For IgG assays, 18 to 22 serial dilutions 1:2 of a positive control were used to perform antigen-isotype/subclass specific standard curves. The positive control consisted of a WHO Reference Reagent for anti-malaria *P. falciparum* human serum (NIBSC code: 10/198) at 1:50 plus a pool of plasmas from RTS,S/AS02 vaccinated children with high IgG titers against CSP at 1:100. For the IgM assay, 18 serial dilutions 1:2 of a pool of samples from ISGlobal repository with high IgM levels against *P. falciparum* antigens were used. A total of 69 different negative control samples from malaria-naïve adult donors were assayed along the study to calculate the cutoffs of seropositivity (mean + 3 standard deviations [SD]). Blanks were added to each plate in triplicates for quality control purposes. Sample distribution across plates was designed to ensure a balanced distribution of vaccination groups, age cohorts and time-points. Data were captured using xPonent software.

**Statistical analysis**

**Data pre-processing.** The assay quality control for each antigen and plate was based on the estimation of the % coefficient of variation (CV) of the 3 blank controls and the performance of the standard curves. The standard curve for each antigen-isotype/subclass-plate was estimated using the *drLumi* R package flow [36], fitted in a 5-parameter logistic (5-PL) regression model, and data points logarithmically transformed. If the model did not converge, 4-PL or exponential regressions were fitted. The standard curves were visually inspected, and the percentage of plates within an analyte-isotype/subclass with CV Emax SE/Beta <15%, CV Emin SE/Beta <15%, R^2^ >0.95 and Model fit p value >0.05, were calculated.

To select the sample dilution in the linear part of the sigmoidal curve (antigen, isotype/subclass and plate specific), an algorithm that detects the two points with the highest slope between them was used. The slope was computed as: m = (log_10_ MFI_i_ – log_10_ MFI_i+1_) / (dilution_factor_i_ – dilution_factor_i+1_). The mean log_10_ MFI value of the two points was computed, and the nearest log_10_MFI of the test sample and the corresponding dilution was selected. The MFI measurement of the selected dilution was corrected multiplying by its corresponding dilution factor and transformed to log_10_ scale to stabilize the variance.

Blank and GST background signals were not subtracted. GST subtraction distorted and increased the variability of the data due to the lack of correlation between GST-fusion proteins and GST alone[37].

**SUPPLEMENTARY RESULTS**

**Additional factors affecting antibody responses**. In adjusted multivariable models, season was not generally associated with antibody levels except for significantly lower IgM levels to about half of the antigens if the post-vaccination visit (M3) occurred during the low compared to the high transmission season (**Table S4**). Sex was not significantly associated with antibody levels at baseline (M0) or M3, except for AMA1 FVO, P41 and RH4.9 for which M3 IgM levels were higher in males than females (**Table S4**).

**Local alignment of antigens to CSP.** The default BLAST E-value threshold of 10 returned hits for two full length sequences in addition to SSP2 (TRAP): MSP2 (a.a. 173-210, % identity = 34.2, E-value = 1.1) and VAR2CSA (a.a. 2003-2022, % identity = 45, E-value = 9.9). These areas of local alignment mapped to CSP a.a. 247-283 and a.a. 349-367, respectively. The region of VAR2CSA, a duffy-binding-like region, maps to the thrombospondin type 1 domain of CSP (PlasmoDB.org). At the peptide level, four 25mer sequences aligned to CSP in addition to the two SSP2 sequences reported in the main text: 1) VAR2CSA a.a. 1999-2023 (% identity = 45, E-value = 2.6) mapped to CSP a.a. 349-367; 2) MSP1 a.a. 379-403 (% identity = 45.5, E-value = 5.3) mapped to CSP a.a. 51-61; 3) VAR2CSA a.a. 361-385 (% identity = 35.3, E-value = 6.2) mapped to CSP a.a. 39-55; and 4) LSA1 a.a. 1099-1123 (% identity = 46.7, E-value = 7.2) mapped to CSP a.a. 307-321.

**REFERENCES**

1. Kolodny N, Kitov S, Vassell MA, Miller VL, Ware LA, Fegeding K, et al. Two-step chromatographic purification of recombinant Plasmodium falciparum circumsporozoite protein from *Escherichia coli*. J Chromatogr B Biomed Sci Appl. 2001;762: 77–86.

2. Yilmaz B, Portugal S, Tran TM, Gozzelino R, Ramos S, Gomes J, et al. Gut microbiota elicits a protective immune response against malaria transmission. Cell. 2014;159: 1277–1289. doi:10.1016/j.cell.2014.10.053

3. Kastenmuller K, Espinosa DA, Trager L, Stoyanov C, Salazar AM, Pokalwar S, et al. Full-length *Plasmodium falciparum* circumsporozoite protein administered with long-chain poly(I.C) or the Toll-like receptor 4 agonist glucopyranosyl lipid adjuvant-stable emulsion elicits potent antibody and CD4+ T cell immunity and protection in mice. Infect Immun. 2013;81: 789–800. doi:10.1128/IAI.01108-12

4. Chaudhury S, Ockenhouse CF, Regules JA, Dutta S, Wallqvist A, Jongert E, et al. The biological function of antibodies induced by the RTS,S/AS01 malaria vaccine candidate is determined by their fine specificity. Malar J. 2016;15: 301. doi:10.1186/s12936-016-1348-9

5. Khusmith S, Charoenvit Y, Kumar S, Sedegah M, Beaudoin RL, Hoffman SL. Protection against malaria by vaccination with sporozoite surface protein 2 plus CS protein. Science. 1991;252: 715–718.

6. Robson KJ, Hall JR, Jennings MW, Harris TJ, Marsh K, Newbold CI, et al. A highly conserved amino-acid sequence in thrombospondin, properdin and in proteins from sporozoites and blood stages of a human malaria parasite. Nature. 1988;335: 79–82. doi:10.1038/335079a0

7. Kusi KA, Bosomprah S, Dodoo D, Kyei-Baafour E, Dickson EK, Mensah D, et al. Anti-sporozoite antibodies as alternative markers for malaria transmission intensity estimation. Malar J. 2014;13: 103. doi:10.1186/1475-2875-13-103

8. Bergmann-Leitner ES, Hosie H, Trichilo J, Deriso E, Ranallo RT, Alefantis T, et al. Self-adjuvanting bacterial vectors expressing pre-erythrocytic antigens induce sterile protection against malaria. Front Immunol. 2013;4: 176. doi:10.3389/fimmu.2013.00176

9. Guerin-Marchand C, Druilhe P, Galey B, Londono A, Patarapotikul J, Beaudoin RL, et al. A liver-stage-specific antigen of *Plasmodium falciparum* characterized by gene cloning. Nature. 1987;329: 164–167. doi:10.1038/329164a0

10. Zhu J, Hollingdale MR. Structure of *Plasmodium falciparum* liver stage antigen-1. Mol Biochem Parasitol. 1991;48: 223–226.

11. Doolan DL, Hedstrom RC, Rogers WO, Charoenvit Y, Rogers M, de la Vega P, et al. Identification and characterization of the protective hepatocyte erythrocyte protein 17 kDa gene of *Plasmodium yoelii*, homolog of *Plasmodium falciparum* exported protein 1. J Biol Chem. 1996;271: 17861–17868.

12. Angov E, Hillier CJ, Kincaid RL, Lyon JA. Heterologous protein expression is enhanced by harmonizing the codon usage frequencies of the target gene with those of the expression host. PLoS One. 2008;3: e2189. doi:10.1371/journal.pone.0002189

13. Kocken CHM, Withers-Martinez C, Dubbeld MA, van der Wel A, Hackett F, Valderrama A, et al. High-level expression of the malaria blood-stage vaccine candidate *Plasmodium falciparum* apical membrane antigen 1 and induction of antibodies that inhibit erythrocyte invasion. Infect Immun. 2002;70: 4471–4476.

14. Reddy KS, Amlabu E, Pandey AK, Mitra P, Chauhan VS, Gaur D. Multiprotein complex between the GPI-anchored CyRPA with PfRH5 and PfRipr is crucial for *Plasmodium falciparum* erythrocyte invasion. Proc Natl Acad Sci U S A. 2015;112: 1179–1184. doi:10.1073/pnas.1415466112

15. Persson KEM, Fowkes FJI, McCallum FJ, Gicheru N, Reiling L, Richards JS, et al. Erythrocyte-binding antigens of *Plasmodium falciparum* are targets of human inhibitory antibodies and function to evade naturally acquired immunity. J Immunol. 2013;191: 785–794. doi:10.4049/jimmunol.1300444

16. Pandey KC, Singh S, Pattnaik P, Pillai CR, Pillai U, Lynn A, et al. Bacterially expressed and refolded receptor binding domain of *Plasmodium falciparum* EBA-175 elicits invasion inhibitory antibodies. Mol Biochem Parasitol. 2002;123: 23–33.

17. Angov E, Aufiero BM, Turgeon AM, Van Handenhove M, Ockenhouse CF, Kester KE, et al. Development and pre-clinical analysis of a *Plasmodium falciparum* Merozoite Surface Protein-1(42) malaria vaccine. Mol Biochem Parasitol. 2003;128: 195–204.

18. Cavanagh DR, McBride JS. Antigenicity of recombinant proteins derived from *Plasmodium falciparum* merozoite surface protein 1. Mol Biochem Parasitol. 1997;85: 197–211.

19. Cowan GJM, Creasey AM, Dhanasarnsombut K, Thomas AW, Remarque EJ, Cavanagh DR. A malaria vaccine based on the polymorphic block 2 region of MSP-1 that elicits a broad serotype-spanning immune response. PLoS One. 2011;6: e26616. doi:10.1371/journal.pone.0026616

20. Metzger WG, Okenu DMN, Cavanagh DR, Robinson J V, Bojang KA, Weiss HA, et al. Serum IgG3 to the *Plasmodium falciparum* merozoite surface protein 2 is strongly associated with a reduced prospective risk of malaria. Parasite Immunol. 2003;25: 307–312.

21. Imam M, Singh S, Kaushik NK, Chauhan VS. *Plasmodium falciparum* merozoite surface protein 3: oligomerization, self-assembly, and heme complex formation. J Biol Chem. 2014;289: 3856–3868. doi:10.1074/jbc.M113.520239

22. Black CG, Wang L, Hibbs AR, Werner E, Coppel RL. Identification of the *Plasmodium chabaudi* homologue of merozoite surface proteins 4 and 5 of *Plasmodium falciparum*. Infect Immun. 1999;67: 2075–2081. Available: https://www.ncbi.nlm.nih.gov/pubmed/10225857

23. Black CG, Barnwell JW, Huber CS, Galinski MR, Coppel RL. The *Plasmodium vivax* homologues of merozoite surface proteins 4 and 5 from *Plasmodium falciparum* are expressed at different locations in the merozoite. Mol Biochem Parasitol. 2002;120: 215–224. doi:https://doi.org/10.1016/S0166-6851(01)00458-3

24. Woehlbier U, Epp C, Hackett F, Blackman MJ, Bujard H. Antibodies against multiple merozoite surface antigens of the human malaria parasite *Plasmodium falciparum* inhibit parasite maturation and red blood cell invasion. Malar J. 2010;9: 77. doi:10.1186/1475-2875-9-77

25. Siddiqui FA, Dhawan S, Singh S, Singh B, Gupta P, Pandey A, et al. A thrombospondin structural repeat containing rhoptry protein from *Plasmodium falciparum* mediates erythrocyte invasion. Cell Microbiol. 2013;15: 1341–1356. doi:10.1111/cmi.12118

26. Taechalertpaisarn T, Crosnier C, Bartholdson SJ, Hodder AN, Thompson J, Bustamante LY, et al. Biochemical and functional analysis of two *Plasmodium falciparum* blood-stage 6-Cys proteins: P12 and P41. PLoS One. 2012;7: e41937. doi: 10.1371/journal.pone.0041937

27. Gaur D, Mayer DCG, Miller LH. Parasite ligand-host receptor interactions during invasion of erythrocytes by *Plasmodium* merozoites. Int J Parasitol. 2004;34: 1413–1429. doi:10.1016/j.ijpara.2004.10.010

28. Sahar T, Reddy KS, Bharadwaj M, Pandey AK, Singh S, Chitnis CE, et al. *Plasmodium falciparum* reticulocyte binding-like homologue protein 2 (PfRH2) is a key adhesive molecule involved in erythrocyte invasion. PLoS One. 2011;6: e17102. doi:10.1371/journal.pone.0017102

29. Reiling L, Richards JS, Fowkes FJI, Barry AE, Triglia T, Chokejindachai W, et al. Evidence that the erythrocyte invasion ligand PfRh2 is a target of protective immunity against *Plasmodium falciparum* malaria. J Immunol. 2010;185: 6157–6167. doi:10.4049/jimmunol.1001555

30. Tham W-H, Wilson DW, Reiling L, Chen L, Beeson JG, Cowman AF. Antibodies to reticulocyte binding protein-like homologue 4 inhibit invasion of *Plasmodium falciparum* into human erythrocytes. Infect Immun. 2009;77: 2427–2435. doi:10.1128/IAI.00048-09

31. Reiling L, Richards JS, Fowkes FJI, Wilson DW, Chokejindachai W, Barry AE, et al. The *Plasmodium falciparum* erythrocyte invasion ligand Pfrh4 as a target of functional and protective human antibodies against malaria. PLoS One. 2012;7: e45253. doi:10.1371/journal.pone.0045253.

32. Reddy KS, Pandey AK, Singh H, Sahar T, Emmanuel A, Chitnis CE, et al. Bacterially expressed full-length recombinant *Plasmodium falciparum* RH5 protein binds erythrocytes and elicits potent strain-transcending parasite-neutralizing antibodies. Infect Immun. 2014;82: 152–164. doi:10.1128/IAI.00970-13

33. Mayor A, Rovira-Vallbona E, Srivastava A, Sharma SK, Pati SS, Puyol L, et al. Functional and immunological characterization of a Duffy binding-like alpha domain from *Plasmodium falciparum* erythrocyte membrane protein 1 that mediates rosetting. Infect Immun. 2009;77: 3857–3863. doi:10.1128/IAI.00049-09

34. Chene A, Gangnard S, Dechavanne C, Dechavanne S, Srivastava A, Tetard M, et al. Down-selection of the VAR2CSA DBL1-2 expressed in *E. coli* as a lead antigen for placental malaria vaccine development. NPJ vaccines. 2018;3: 28. doi:10.1038/s41541-018-0064-6

35. Gangnard S, Lewit-Bentley A, Dechavanne S, Srivastava A, Amirat F, Bentley GA, et al. Structure of the DBL3X-DBL4epsilon region of the VAR2CSA placental malaria vaccine candidate: insight into DBL domain interactions. Sci Rep. 2015;5: 14868. doi:10.1038/srep14868

36. Sanz H, Aponte JJ, Harezlak J, Dong Y, Ayestaran A, Nhabomba A, et al. drLumi: An open-source package to manage data, calibrate, and conduct quality control of multiplex bead-based immunoassays data analysis. PLoS One. 2017;12: 1–18. doi:10.1371/journal.pone.0187901

37. Ubillos I, Jiménez A, Vidal M, Bowyer PW, Gaur D, Dutta S, et al. Optimization of incubation conditions of *Plasmodium falciparum* antibody multiplex assays to measure IgG, IgG1-4, IgM and IgE using standard and customized reference pools for sero-epidemiological and vaccine studies. Malar J. 2018;17: 1–15. doi:10.1186/s12936-018-2369-3
